# *Plasmodium falciparum* dipeptidyl aminopeptidase 3 activity is important for efficient erythrocyte invasion by the malaria parasite

**DOI:** 10.1371/journal.ppat.1007031

**Published:** 2018-05-16

**Authors:** Christine Lehmann, Michele Ser Ying Tan, Laura E. de Vries, Ilaria Russo, Mateo I. Sanchez, Daniel E. Goldberg, Edgar Deu

**Affiliations:** 1 Chemical Biology Approaches to Malaria Laboratory, The Francis Crick Institute, London, United Kingdom; 2 Department of Medical Microbiology, Radboud University Medical Center, Nijmegen, Netherlands; 3 Faculty of Life Sciences, University of Manchester, Manchester, United Kingdom; 4 Department of Genetics, Stanford School of Medicine, Stanford, California, United States of America; 5 Departments of Molecular Microbiology and Medicine, Washington University School of Medicine, St Louis, Missouri, United States of America; University of Geneva, SWITZERLAND

## Abstract

Parasite egress from infected erythrocytes and invasion of new red blood cells are essential processes for the exponential asexual replication of the malaria parasite. These two tightly coordinated events take place in less than a minute and are in part regulated and mediated by proteases. Dipeptidyl aminopeptidases (DPAPs) are papain-fold cysteine proteases that cleave dipeptides from the N-terminus of protein substrates. DPAP3 was previously suggested to play an essential role in parasite egress. However, little is known about its enzymatic activity, intracellular localization, or biological function. In this study, we recombinantly expressed DPAP3 and demonstrate that it has indeed dipeptidyl aminopeptidase activity, but contrary to previously studied DPAPs, removal of its internal prodomain is not required for activation. By combining super resolution microscopy, time-lapse fluorescence microscopy, and immunoelectron microscopy, we show that *Plasmodium falciparum* DPAP3 localizes to apical organelles that are closely associated with the neck of the rhoptries, and from which DPAP3 is secreted immediately before parasite egress. Using a conditional knockout approach coupled to complementation studies with wild type or mutant DPAP3, we show that DPAP3 activity is important for parasite proliferation and critical for efficient red blood cell invasion. We also demonstrate that DPAP3 does not play a role in parasite egress, and that the block in egress phenotype previously reported for DPAP3 inhibitors is due to off target or toxicity effects. Finally, using a flow cytometry assay to differentiate intracellular parasites from extracellular parasites attached to the erythrocyte surface, we show that DPAP3 is involved in the initial attachment of parasites to the red blood cell surface. Overall, this study establishes the presence of a DPAP3-dependent invasion pathway in malaria parasites.

## Author summary

Malaria remains one of the most devastating infectious diseases and its clinical manifestation is caused by the exponential multiplication of parasites in patients. This asexual replication cycle consists of red blood cell invasion, intracellular parasite multiplication, and release (also known as egress) of daughter parasites for further red blood cell invasion. Host cell invasion is therefore essential for parasite replication and the only moment in this cycle when parasites are exposed to the immune system. Understanding the molecular mechanisms that control red blood cell invasion might not only lead to the identification of novel antimalarial targets but also to the development of better invasion blocking vaccines. DPAP3 is a druggable cysteine protease that was previously believed to be essential for parasite egress. In this study, we show that parasites lacking DPAP3 activity are unable to efficiently invade red blood cells but escape the confines of the host cell normally. Overall, this study increases our understanding of the proteolytic pathways that govern host cell invasion by the malaria parasite.

## Introduction

Malaria is a devastating infectious disease caused by Apicomplexan parasites of the *Plasmodium* genus and is transmitted by Anopheles mosquitoes during a blood meal. After an initial asymptomatic liver infection, parasites are released into the blood stream where they replicate within red blood cells (RBCs). This asexual exponential growth is responsible for all the pathology associated with malaria, causing close to half a million deaths every year[1]. Over the last 15 years, the world has seen a significant decrease in malaria incidence mainly due to the distribution of insecticide-impregnated bed nets and the introduction of ACT (artemisinin-based combination therapy) as the standard of care for uncomplicated malaria[2]. However, the recent emergence of artemisinin resistance[3] has made the identification of viable therapeutic targets extremely important[4,5].

*P*. *falciparum* is the most virulent *Plasmodium* species accounting for most of malaria mortality. Its 48 h asexual erythrocytic cycle consists of RBC invasion, intraerythrocytic parasite growth and division into 16–32 daughter merozoites, followed by parasite egress for further RBC invasion. Parasite egress and RBC invasion are key for parasite replication and blocking either one of these processes would lead to a quick drop in parasitemia and malaria pathology. Proteases have been shown to play essential roles in both processes and might therefore be viable therapeutic targets[6].

RBC invasion is a multistep process involving initial recognition of RBC receptors by adhesin proteins on the surface of the merozoite (invasive extracellular parasite form), tight attachment to the RBC membrane (RBCM), reorientation of the merozoite apical end towards the RBCM, active invasion driven by an actin-myosin motor with invagination of the RBCM and formation of the parasitophorous vacuole (PV), and finally, sealing of the RBCM and PV membrane (PVM)[7,8]. The PV is a membrane-bound vacuole within which the parasite grows and replicates isolated from the RBC cytosol. Rupture of the PV and RBC membranes is required for parasite egress and is mediated by proteases. In particular, subtilisin-like protease 1 (SUB1), an essential serine protease residing in apical secretory organelles known as exonemes, is released into the PV right before egress where it processes several proteins important for egress and invasion[9–13]. These include cleavage and likely activation of serine repeat antigen 6 (SERA6)[14], an essential papain-fold cysteine protease[15–17].

In a forward chemical genetic approach, *P*. *falciparum* dipeptidyl aminopeptidase 3 (DPAP3) was identified as a potential regulator of parasite egress acting upstream of SUB1[18]. DPAPs are papain-like cysteine proteases that cleave dipeptides off the N-terminus of protein substrates[19]. In that study [18], the vinyl sulfone inhibitor SAK1 was shown to preferentially inhibit DPAP3 over other cysteine proteases. This compound arrests parasite egress at mid-micromolar concentrations, blocks processing of SUB1 substrates, and prevents proper expression and maturation of SUB1 and apical membrane antigen 1 (AMA1), a micronemal protein secreted onto the merozoite surface that is essential for RBC invasion[9,20,21]. These results led to the hypothesis that DPAP3 might act as a general maturase of secretory proteins involved in egress and invasion. However, while the function (or essentiality) of *P*. *falciparum* DPAP3 has not been validated genetically, in the rodent parasite *P*. *berghei*, DPAP3 knock out (KO) parasites are viable but replicate significantly slower[22–24].

Here, we combine chemical, biochemical and conditional genetic approaches to show that DPAP3 is an active protease that resides in apical secretory organelles, and that its activity is critical for efficient RBC invasion. We also provide very strong evidence showing that DPAP3 does not play a significant function in parasite egress.

## Results

### DPAP3 activity is important for parasite viability

**Fig 1:**
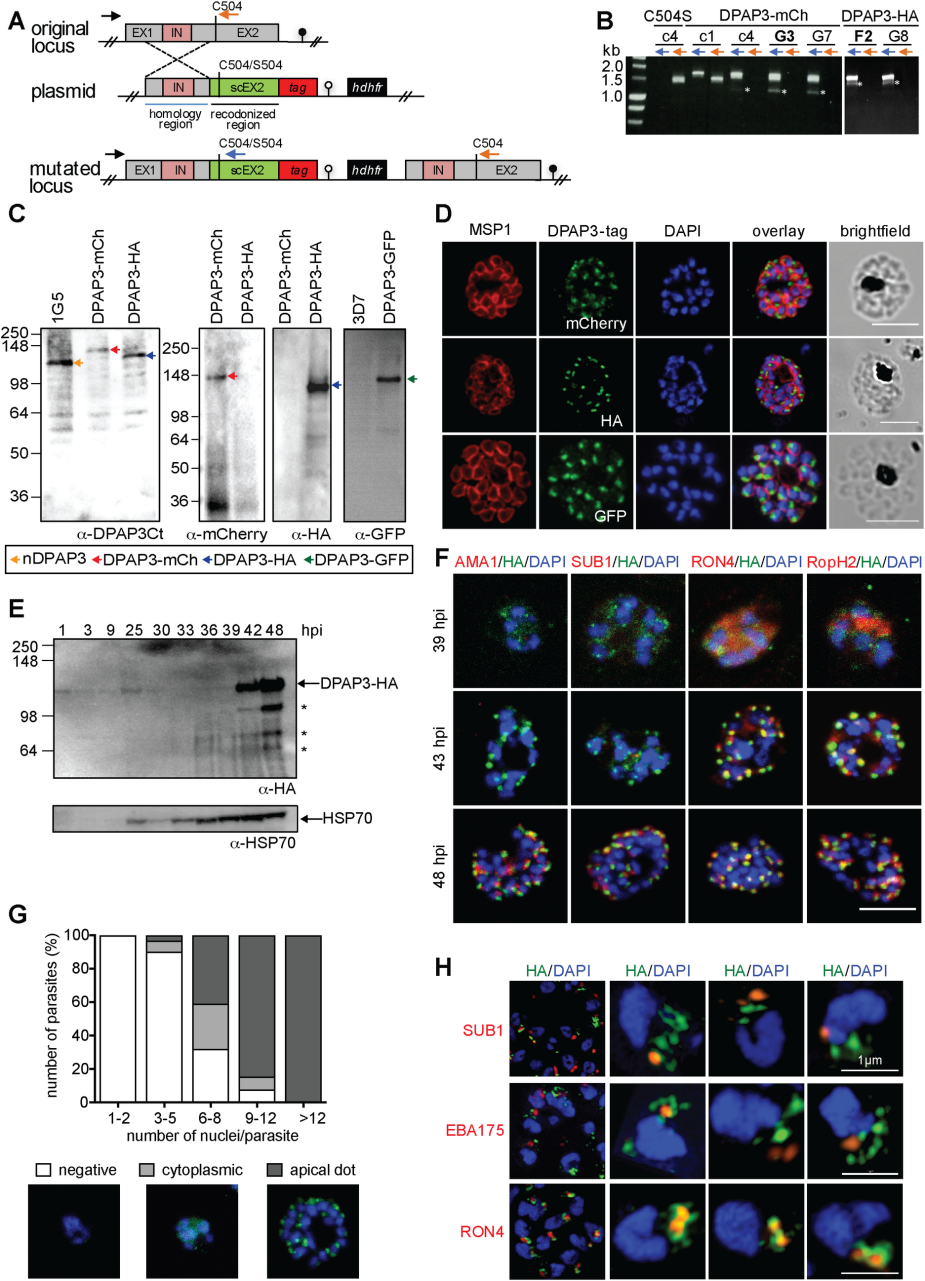
DPAP3 is expressed during schizogony and localizes at the apical pole. (**A**) Scheme showing the *dpap3* endogenous locus (exons, grey; intron, pink) targeted by single homologous recombination with plasmids containing a homology region (blue line), a recodonized C-terminal region (sc) tagged with HA_3_ or mCherry and containing either WT Cys504 or the C504S mutation, and the *hdhfr* drug resistant cassette. (**B**) Integration assessed by PCR on genomic DNA using the P21 (black arrow) and P22 (orange arrows) or P23 (blue arrow) primers for the endogenous or modified locus, respectively. Primer binding sites are indicated in A. PCR obtained after one (c1) or four (c4) drug cycles, and for the DPAP3-mCh and DPAP3-HA clones are shown. Asterisks indicate non-specific PCR products. (**C**) WB analysis of schizont stage parasites from the DPAP3-mCh, -HA, and -GFP clones and the 1G5 and 3D7 control lines using anti-DPAP3-Ct, -HA, -mCherry, or -GFP antibodies. Bands corresponding to native or tagged DPAP3 are indicated with arrows. (**D**) IFAs of DPAP3-mCh, DPAP3-HA, and DPAP3-GFP mature schizonts stained with anti-MSP1 (red), and anti-mCherry, -HA, or -GFP (green) antibodies (confocal microscopy). (**E**) WB of DPAP3-HA parasite lysates harvested at different h.p.i. DPAP3-HA was detected using anti-HA antibody. HSP70 was used as a loading control. Asterisks indicate DPAP3 degradation products. (**F**) IFA of DPAP3-HA schizonts collected 39–48 h.p.i. and stained with anti-HA (green) and anti-AMA1 (microneme), anti-SUB1 (exoneme), anti-RON4 (rhoptry neck), or anti-RopH2 (rhoptry bulb) antibodies (all red). Obtained by confocal microscopy; single staining images are shown in S2 Fig. (**G**) Quantification of DPAP3-HA parasites showing negative, cytoplasmic or apical DPAP3 staining during schizogony. Samples were fixed from 36–48 h.p.i. and stained with anti-HA (green). Schizont maturity was assigned based on the number of nuclei. A representative image for each staining phenotype is shown. (**H**) IFA of C2-arrested DPAP3-HA schizonts analyzed by SIM. Parasites were stained with anti-HA (green) and anti-SUB1, anti-EBA175, or anti-RON4 antibodies (all red). Representative 3D-reconstruction images of merozoites are shown. In all IFAs DNA was stained with DAPI (blue); scale bars = 5 μm except for merozoite images in H (1 μm).

Using single homologous recombination we were able to replace the endogenous catalytic domain of *dpap3* with a C-terminally tagged (GFP, mCherry or HA) version. However, our multiple attempts to replace the DPAP3 catalytic Cys with a Ser failed despite using the same homology region upstream of the catalytic domain (Fig 1A and 1B and S1A and S1B Fig). Our attempts to KO *dpap3* by double homologous recombination also failed. These results strongly suggest that DPAP3 activity is important for parasite development. Parasites containing differently tagged *dpap3* were cloned by limited dilution, and the clones selected for this study will be referred to as DPAP3-GFP, DPAP3-mCh, and DPAP3-HA. Analysis of parasite extracts by western blot (WB) using a polyclonal antibody that targets the C-terminal half of DPAP3 showed a shift in migration pattern in accordance with the tag molecular weight (Fig 1C).

### PfDPAP3 localizes to an apical secretory organelle

In all our tagged lines, DPAP3 consistently localizes to the apical pole of merozoites (Fig 1D). To determine when it is expressed, tightly synchronized DPAP3-HA and DPAP3-mCh parasites were collected every 3 h throughout the erythrocytic cycle and were either lysed and analyzed by WB (Fig 1E), or fixed for immunofluorescence analysis (IFA, Fig 1F and S1C Fig). Consistent with its transcription profile[25], DPAP3 is most abundant in late schizonts and merozoites, but could also be detected in rings and trophozoites by WB. To confirm that the small amount of DPAP3 observed by WB at ring stage is not due to schizonts contamination in our cultures, we analyzed 50 fields of five Giemsa-stained thin blood smears at 1 hour post invasion (h.p.i.), i.e. around 75,000 RBCs. We did not observe any schizonts in these slides, only ring stage parasites at around 5% parasitemia. Also, WB analysis using an MSP1 (merozoite surface protein 1) antibody as a marker of schizogony showed no significant staining at ring or trophozoite stages (S1D Fig). Although we could detect multiple processed forms of DPAP3 by WB, these processed forms are rarely observed in live parasites (Fig 1C) and are likely an artefact of parasite lysis (see below).

By IFA, DPAP3 was first detected in young schizonts (6–8 nuclei) and seems to be expressed at the same time as rhoptry (rhoptry neck protein 4, RON4, and high molecular weight rhoptry protein 2, RopH2) and inner membrane complex (glideosome-associated protein 45, GAP45) proteins (Fig 1F and S1 and S2 Figs). During schizont maturation, DPAP3 and rhoptry proteins localization changes from a diffuse and granular cytosolic staining to a clear punctuated apical staining in daughter merozoites (Fig 1F and 1G and S1E and S2 Figs), probably reflecting protein trafficking and organelle biogenesis. By contrast, exonemal (SUB1) and micronemal proteins (AMA1) are expressed and localize to their apical organelles at a later stage (Fig 1F and S2 Fig). Note that the diffuse staining observed at 39 h.p.i. for DPAP3, RON4 and RopH2 is not background fluorescence signal given that no staining was observed in these same slides for the few parasites that were lagging behind in development, i.e. infected RBCs (iRBCs) with a single nucleus (S2A Fig).

Using standard confocal microscopy, we could not observe consistent colocalization of DPAP3 with any apical organelle marker tested: AMA1 and EBA175 (erythrocyte binding antigen 175) for micronemes, SUB1 for exonemes, RON4 and RopH2 for rhoptries, and Exp2 (exported protein 2) for dense granules (Fig 1F and S2B Fig). We therefore decided to increase the resolution of our images by using structured illumination microscopy (SIM). By SIM we could clearly observe DPAP3 staining in small but well-defined dot-like structures at the apical pole of each merozoite in the DPAP3-HA (Fig 1H and S3A Fig) and DPAP3-GFP (S3B Fig) lines. Although we did not observe colocalization with exoneme (SUB1), microneme (EBA175) or rhoptry (RON4, RopH2) protein markers (Fig 1H and S3A and S3B Fig), DPAP3 seems to be closely associated with RON4, suggesting that it resides in apical organelles that surround the neck of the rhoptries (Fig 1H).

### PfDPAP3 is secreted after PVM breakdown

**Fig 2:**
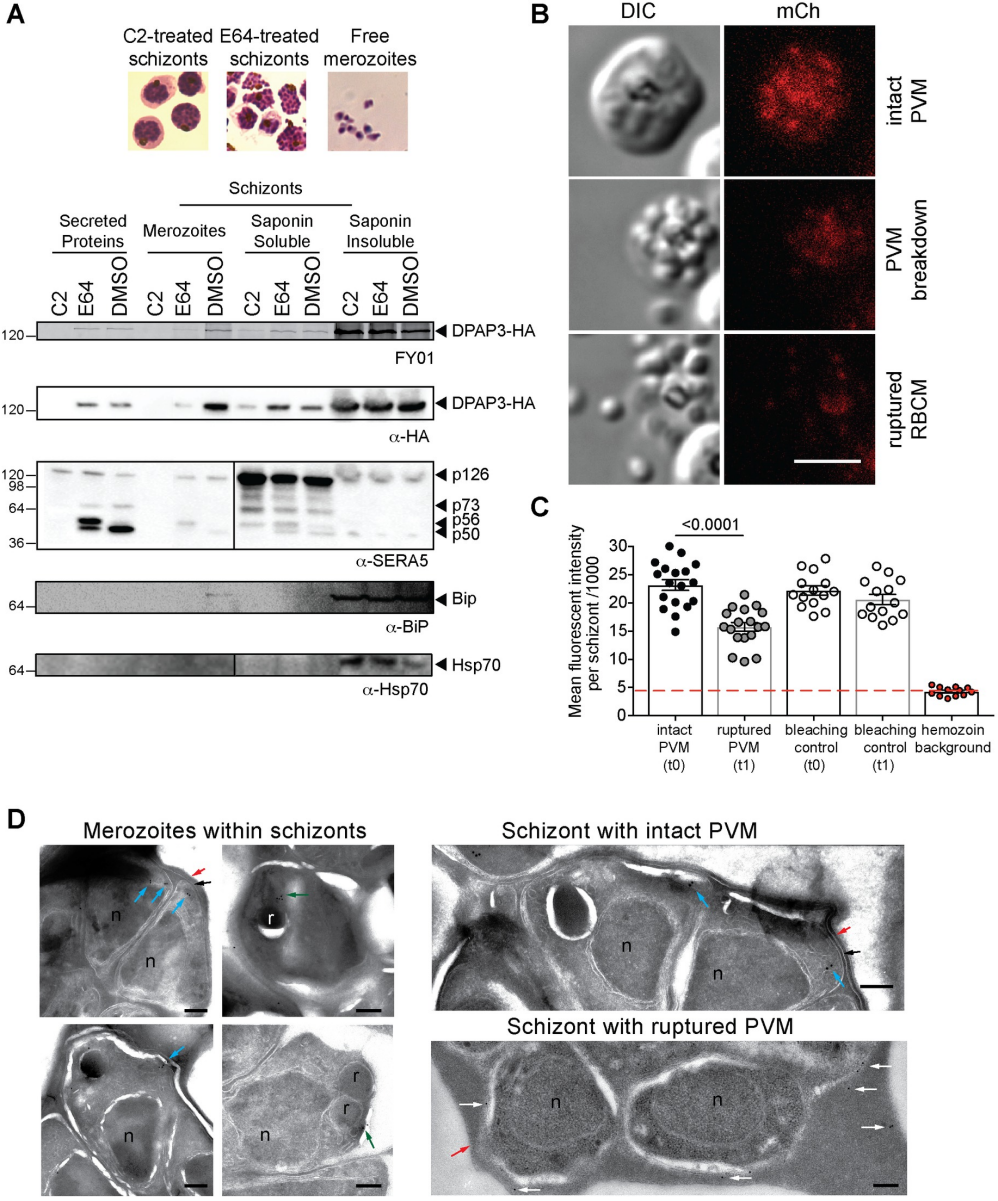
DPAP3 is secreted at the time of egress. (**A**) C2-arrested schizonts (DPAP3-HA) were either left on C2, treated with E64 after C2 wash out, or allowed to egress for 1 h in the presence of FY01. Parasite pellets from free merozoites and schizonts (insoluble fraction obtained after saponin lysis), proteins precipitated from the culture supernatant, and PV and RBC cytosol components (soluble saponin fraction), were run on a SDS-PAGE. The presence of DPAP3-HA in each fraction was visualized as a fluorescent band at around 130kDa, which correspond to the band identified by WB using an anti-HA antibody (See also S4A Fig). Hsp70 and BiP antibodies were used as WB markers of intracellular proteins (cytosol and ER, respectively), and SERA5 as a PV marker. (**B**) Mature DPAP3-mCh schizonts were arrested with C2 for 3 h, and egress observed by live video microscopy after C2 wash out (S1–S3 Videos). The representative still-frame pictures show DIC and mCherry signal (red) before or after PVM breakdown, and after RBCM rupture. (**C**) Quantification of mCherry signal measured on consecutive frames before and after PVM breakdown. Around 20% of the signal originates from the hemozoin autofluorescence (red line). As a bleaching control, the mCherry signal of schizonts that did not egress was quantified at the corresponding time frames. (**D**) IEM section obtained from DPAP3-GFP parasites. Close-up images of individual intracellular merozoites on the left show immunogold staining of DPAP3-GFP in close proximity to the rhoptries (green arrows) and at the apical end of merozoites (blue arrows). Images on the right show representative sections of schizonts with an intact (black arrows) or rupture PVM. Staining of extracellular DPAP3-GFP (white arrows) was only observed in schizonts lacking a PVM. Rhoptries (r), nuclei (n), and the RBCM (red arrows) are indicated. Rabbit anti-GFP and colloidal gold-conjugated anti-rabbit antibodies were used. Bar graph = 200 nm. IEM images obtained on the 3D7 control line are shown in S3D Fig, and the uncropped IEM images for the DPAP3-GFP line in S3E Fig.

Timely discharge of proteins from the different apical organelles is crucial to regulate parasite egress and RBC invasion[26]. Secretion of exonemal and micronemal proteins is mediated through activation of cGMP-dependent protein kinase G (PKG), which takes places 15–20 min before egress[9]. Release of SUB1 into the PV leads to parasite egress. Secretion of micronemal proteins, such as AMA1 or EBA175, onto the parasite surface is essential for merozoite attachment to the RBCM and invasion. To determine whether DPAP3 is secreted, we used the DPAP3-HA line to compare the level of DPAP3 present within parasites, in the PV and RBC cytosol, and in the culture supernatant at three different stages of egress: schizonts arrested before exoneme/microneme secretion with the PKG reversible inhibitor compound 2 (C2), schizonts arrested between PVM and RBCM breakdown using the general cysteine protease inhibitor E64, and free merozoites collected after egress. In E64-arrested schizonts, the erythrocyte is still intact but the RBCM is highly porated allowing leakage of RBC and PV proteins into the culture supernatant[17,27]. Parasite pellets, PV and RBC cytosol proteins, and proteins secreted in the culture supernatant under these three conditions were collected after treating the cultures with the fluorescent activity-based probe FY01[28]. FY01 is a cell-permeable probe that covalently modifies the catalytic Cys of cysteine proteases including DPAP3[18]. Fluorescently labelled DPAP3-HA can then be visualized by in-gel fluorescence in a SDS-PAGE gel as a band running around 130 kDa that matches the WB band observed with a HA antibody (Fig 2A and S4A Fig). DPAP3 was mainly detected in the culture supernatant and saponin soluble fraction of E64-arrested or rupturing schizonts, but not in C2-arrested schizonts, indicating that it is secreted downstream of PKG activation but before merozoites become extracellular. The presence of DPAP3 in E64-arrested schizont pellets and free merozoites suggests only partial secretion before egress. To confirm proper fractionation of our samples, we used SERA5, BiP (binding immunoglobin protein) and Hsp70 (heat shock protein 70) as PV, ER, and cytosol protein markers, respectively, in WB analysis (Fig 2A). Processing of SERA5 was also used to confirm that C2 or E64 treatments arrested parasite development at the expected stage. Upon exoneme secretion, SUB1 sequentially cleaves SERA5 into a 73 and 56 kDa forms. This processing is blocked by C2 since exoneme secretion is regulated by PKG. SERA5 is further processed into a 50 kDa form by an unknown cysteine protease that is inhibited by E64. Two additional biological replicates showing secretion of DPAP3 are shown in S4B and S4C Fig.

To determine the timing of DPAP3 secretion, DPAP3-mCh schizonts were arrested with C2 and parasite egress monitored by live microscopy after C2 wash out. Using this assay, PVM breakdown is clearly observable on the DIC channel when merozoites become more spread out within the RBC and their shape is better defined[12,27]. This is followed by RBCM rupture and merozoites dispersal (Fig 2B and S1–S3 Videos). Quantification of fluorescence signal in these egress videos shows a 40% decrease in mCherry signal right after PVM breakdown but before RBCM rupture (Fig 2C). Interestingly, conditional KO (cKO) of SUB1 has recently been shown to prevent breakdown of the PVM but not AMA1 secretion, suggesting that microneme secretion takes place before PVM breakdown [17]. This implies that DPAP3 secretion probably takes place downstream of microneme secretion and coincides with PVM breakdown.

To confirm that DPAP3 is secreted into the RBC cytosol after PVM breakdown, we performed immunoelectron microscopy (IEM) on DPAP3-GFP schizonts collected at the time of egress (Fig 2D and S3D and S3E Fig). In schizonts containing an intact PVM, DPAP3-GFP staining was observed at the apical end of merozoites, and in some sections, in close proximity to the rhoptries or enclosed within membrane bound vesicles, thus supporting our IFA results. In IEM images collected on schizonts after PVM breakdown, immunostaining was mainly observed in the RBC cytosol. Almost no DPAP3 staining was detected in the PV of schizonts with an intact PVM.

### Full-length DPAP3 has proteolytic activity

DPAPs are generally processed from a zymogen form (full-length protein after removal of the signal peptide) into an active form through removal of an internal prodomain and cleavage of the catalytic domain into two polypeptides[29,30] (S5A Fig). Three different isoforms of DPAP3 (p120, p95, and p42) consistent with the canonical processing of DPAPs were previously shown to be labelled by FY01 in merozoite lysates[18]. However, we have now shown that this processing is an artefact of parasite lysis (S5B–S5D Fig and S1 Text). In addition, our WB and FY01-labelling experiments show that full length DPAP3 (p120) is the predominant form found in live parasites (Figs 1 and 2 and S5 Fig).

**Fig 3:**
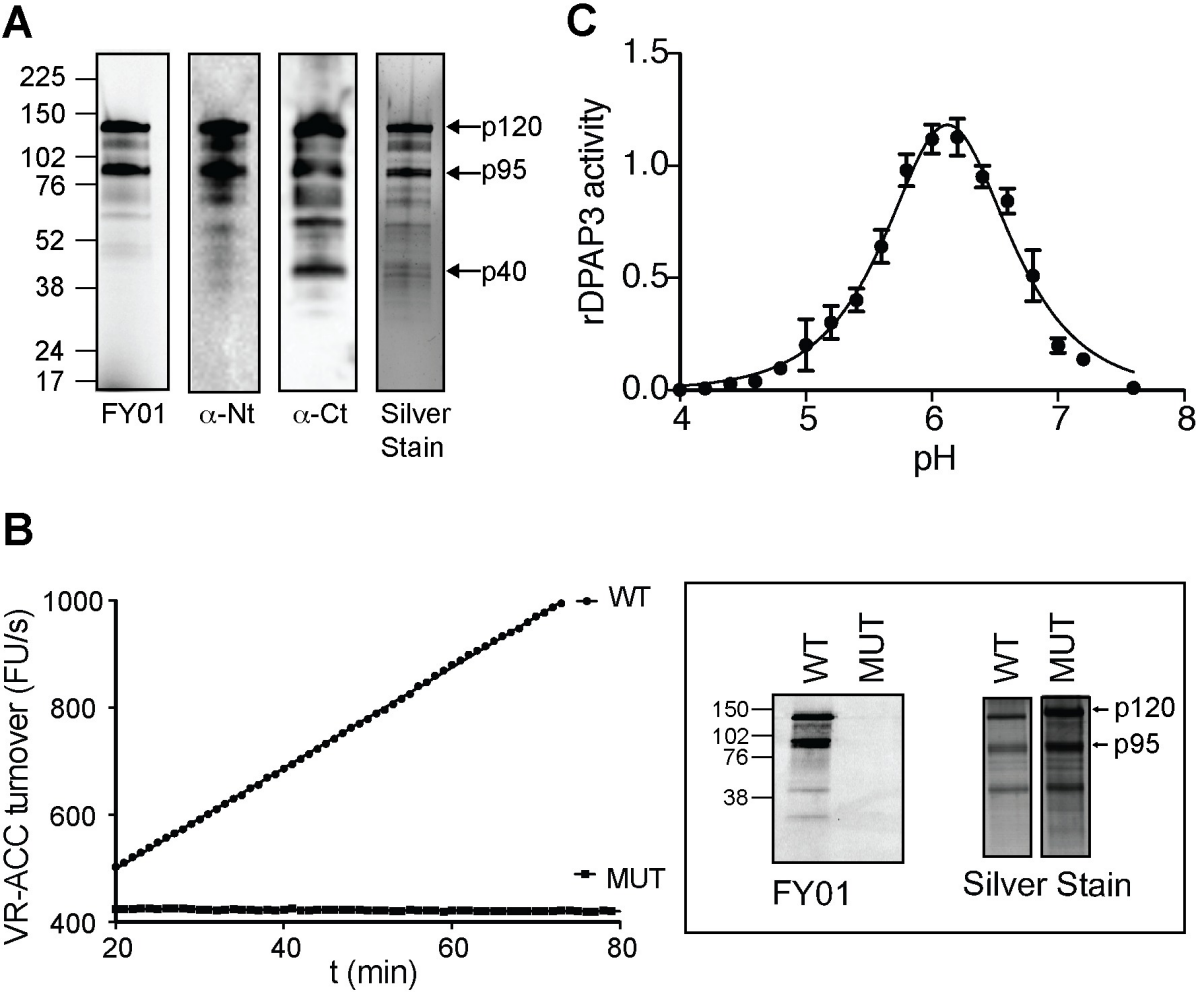
DPAP3 has proteolytic activity. (**A**) Analysis of purified rDPAP3. Two main bands are detected by silver stain, both of which are strongly labelled by FY01 and recognized by the anti-Nt-DPAP3 and anti-Ct-DPAP3 antibodies. All other minor bands in the silver stain are also recognized by DPAP3 antibodies and represent degradation products that could not be separated during purification. (**B**) Measurement of VR-ACC turnover and FY01 labelling for WT and C504S MUT rDPAP3. Silver stain analysis shows equivalent amounts of protein were obtained from the purification of WT and MUT rDPAP3. (**C**) pH dependence of rDPAP3 activity measured at 10 μM VR-ACC (n = 3).

To determine whether this full-length p120 form is active, we recombinantly expressed wild type (WT) and mutant (MUT, replacement of the catalytic Cys504 to Ser) DPAP3 in insect cells using the baculovirus system[31]. Expression and purification of recombinant DPAP3 (rDPAP3) from insect cells culture supernatant yielded predominantly the p120 and p95 forms (Fig 3A and S5B Fig). While WT DPAP3 is able to efficiently cleave the VR-ACC fluorogenic DPAP substrate[32], no activity was observed with MUT DPAP3 (Fig 3B). We also show that rDPAP3 is active under mild acidic conditions with an optimal pH of 6 (Fig 3C). Importantly, in one of our purifications we were able to separate the p120 form from a fraction containing a mixture of the p95 and p120 forms. We used these fractions to show that the p120 form is fully active (S5E Fig). This result strongly suggests that the predominant DPAP3 form present in live parasites (p120) is the one performing a biological function.

### Generation of DPAP3 cKO and complementation lines

Since we were unable to directly KO DPAP3, we generated DPAP3 cKO lines on the 1G5 parasite line background that endogenously expressed DiCre[33]. In the DiCre system, Cre recombinase is split into two domains fused to rapamycin (RAP) binding domains. Addition of RAP triggers dimerization and activation of DiCre, leading to rapid recombination of specific DNA sequences known as *loxP* sites[34,35]. We used this system to conditionally truncate the catalytic domain of DPAP3 rather than excising the full gene to prevent potential episomal expression of DPAP3 after excision.

**Fig 4:**
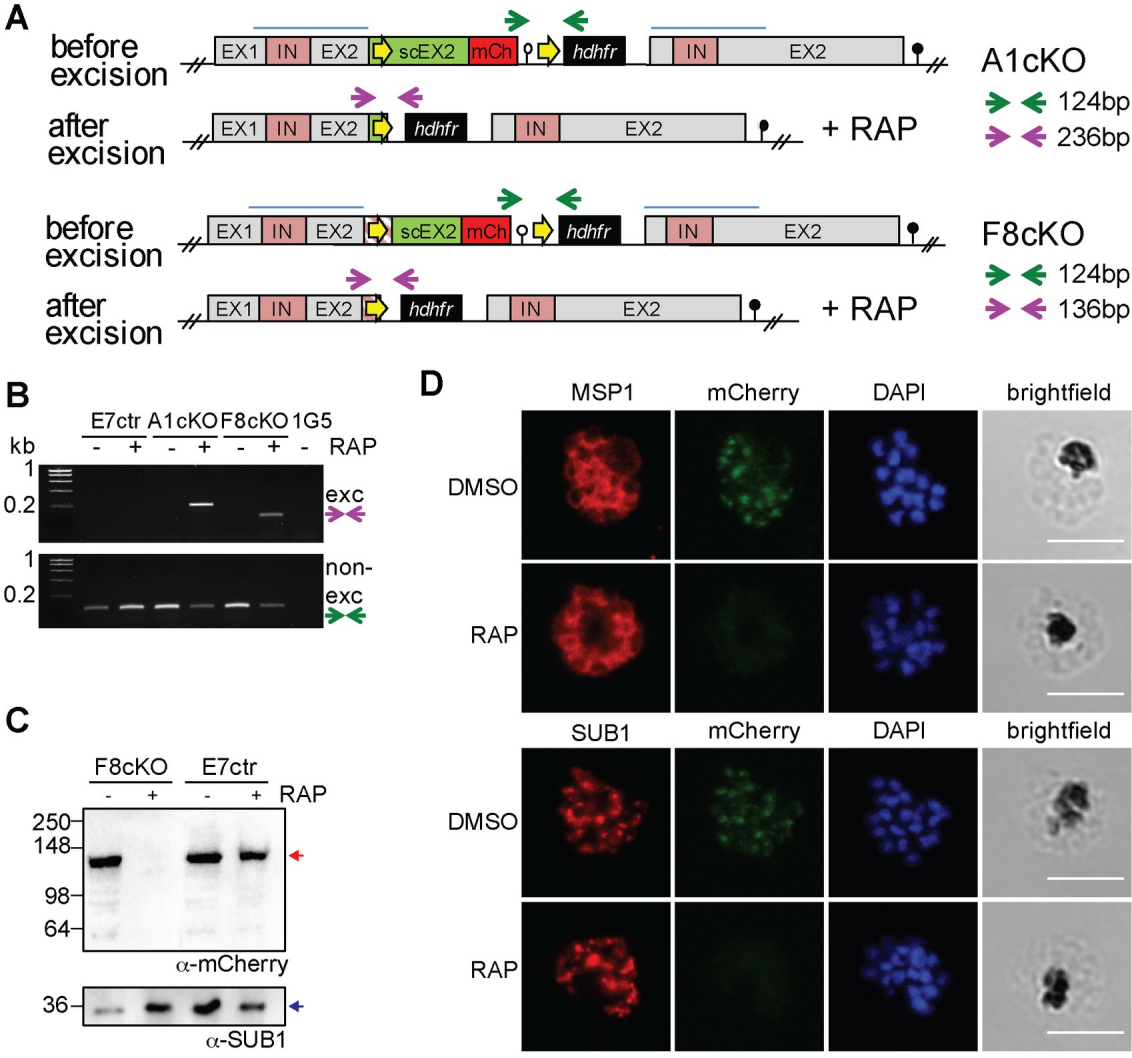
Generation of DPAP3 cKO lines. (**A**) Schematic representation and assessment of the *dpap3* recombinant genetic locus before and after RAP-mediated excision for the F8cKO and A1cKO lines. Wild type exons (EX) and intron (IN) sequences are depicted with grey and pink boxes, respectively. The homology regions used for single crossover recombination are indicated with blue lines, and the recodonized 3’ end of the second exon is shown in green (scEX2). *loxP* sites (yellow arrows) were introduced downstream of the *P*. *berghei* 3’UTR (white circle) and either within the ORF of scEX2 (A1cKO) or upstream of scEX2 (F8cKO) as a *loxPint* artificial intron (pink striped box). The *mCherry* coding sequence (red box), the *hdhfr* resistance cassette (black box), and the displaced endogenous *dpap3* locus with its 3’UTR (black circle) are also shown. Arrows indicate primers annealing sites used for diagnostic PCR of excised (purple) and non-excised (green) loci. (**B**) Diagnostic PCR showing excision at the *dpap3*-locus. PCR was performed on genomic DNA collected from the E7ctr, A1cKO and F8cKO lines 24 h after DMSO or RAP treatment. Genomic DNA from the parental 1G5 line was used as a negative control. Excision and non-excision PCR products are indicated with purple and green arrows, respectively. Excision product was only observed after RAP treatment of the cKO lines. The presence of a non-excised PCR product after RAP treatment indicate that excision is not 100% efficient. (**C**) WB analysis showing highly efficient loss of DPAP3 upon RAP treatment. Schizonts collected 45 h after DMSO or RAP treatment of E7ctr and F8cKO parasites were saponin lysed, and the parasite pellet analyzed by WB using an anti-mCherry antibody (red arrow). SUB1 was used as a loading control (blue arrow). (**D**) IFA analysis of mature schizonts showing the loss of DPAP3 signal after RAP treatment. Ring-stage F8cKO parasites were treated with DMSO or RAP for 3 h and fixed for IFA analysis at 48 h.p.i. Slides were stained with anti-mCherry (green) and anti-MSP1 (red) or anti-SUB1 (red) antibodies. DNA was stained with DAPI (blue); scale bar: 5 μm.

**Fig 5:**
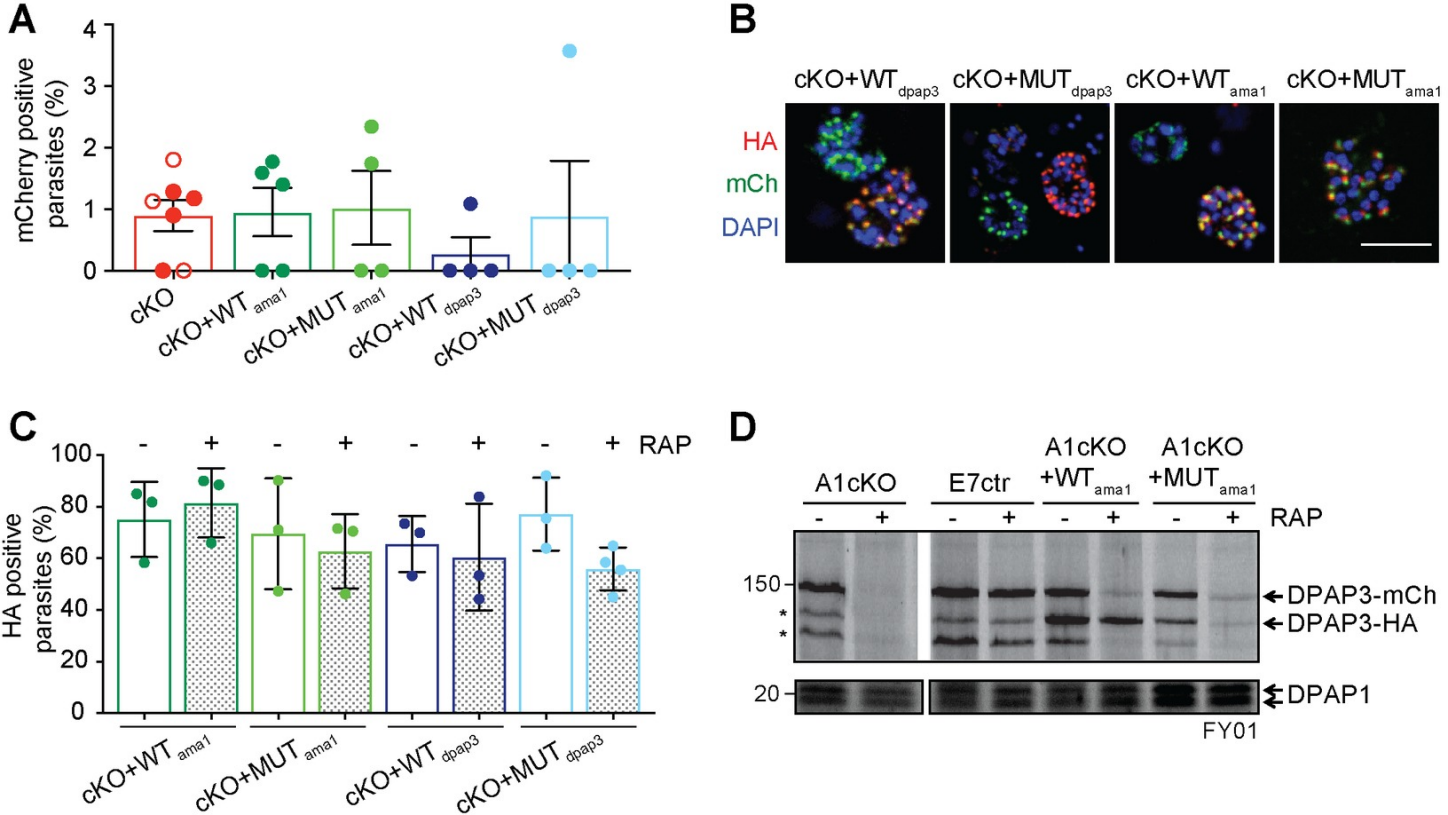
Generation of complementation lines. (**A**) Quantification of excision efficiency for DPAP3cKO and complementation lines. Schizonts collected after DMSO or RAP treatment were stained with anti-mCherry and anti-SUB1 antibodies. SUB1 staining was used as a marker of schizont maturity. The amount of mCherry positive schizonts was quantified in relation to the total amount of mature schizonts. In DMSO treated parasites, all mature schizonts were mCherry positive (100%). The amount of non-excised parasites after RAP treatment was <5% in all biological replicates for all the cKO and complementation lines tested. Each circle corresponds to a different biological replicate (>100 schizonts were analyzed per biological isolate). Filled circles correspond to F8cKO and its complementation lines, and empty ones to the A1cKO line. (**B**) IFA analysis of the complementation lines showing colocalization of chromosomal DPAP3-mCh and episomal DPAP3-HA expressed under the *dpap3* or *ama1* promoters. Mature schizonts from the F8cKO+WT_dpap3_, F8cKO+MUT_dpap3_, F8cKO+WT_ama1_, and F8cKO+MUT_ama1_ were fixed and stained with anti-HA (red) and anti-mCherry (green). DNA was stained with DAPI (blue); scale bar: 5μm. Single coloured images are shown in S6C Fig. (**C**) Quantification of the amount of DPAP3-HA positive schizonts in the complementation lines. Schizonts were fixed at 48 h.p.i. and stained with anti-HA and anti-SUB1 antibodies. Only 60–80% of mature schizonts show positive HA staining with no difference between DMSO and RAP treated parasites. More than 100 schizonts per biological replicate were analyzed. (**D**) FY01 labelling of cKO and complementation lines. After DMSO or RAP treatment at ring stage, C2-arrested schizonts were collected, lysed, and labelled with FY01. Labelling of chromosomal DPAP3-mCh is clearly visible as a band around 150kDa along with some post-lysis degradation products indicated by asterisks. The loss of this 150kDa upon RAP treatment is observed in all lines except the E7ctr. Episomal WT DPAP3-HA is labelled by FY01 independently of RAP treatment and co-migrates with one of the degradation products of DPAP3-mCh at 125kDa. No labelling of MUT DPAP3-HA was observed. DPAP1 labelling by FY01 is shown as a loading control.

Two independent strategies, which differ in how the first *loxP* site was introduced within the *dpap3* open reading frame (ORF), were used (Fig 4A and S6A Fig). In both cases, one *loxP* site was introduced downstream of the 3’-UTR. The other was inserted either within an Asn-rich region of DPAP3 predicted not to interfere with folding or catalysis, or within an artificial intron (*loxPint*), which does not alter the ORF of the targeted gene and has been shown to be well tolerated in several *P*. *falciparum* genes[12,17,36,37]. In both instances, the recodonized catalytic domain was tagged with mCherry such that RAP-induced truncation would result in the loss of mCherry signal. A control line containing only the 3’-UTR *loxP* site was also generated. After transfection of 1G5 parasites, drug selection, and cloning, three DPAP3cKO clones (F3cKO and F8cKO with *loxPint*, and A1cKO with *loxP* in Asn-stretch), and the E7ctr line (only one *loxP*) were selected for further studies. Evidence of integration by PCR is shown in S6B Fig. Analysis of genomic DNA of DMSO- or RAP-treated cKO lines by PCR showed highly efficient excision (Fig 4B) resulting in the loss of DPAP3-mCh expression in mature schizonts (Fig 4C and 4D). Although we consistently achieved more than 95–99% excision efficiency (Fig 5A), a fraction of non-excised parasites was always present after RAP treatment, which explains the presence of a non-excision DNA band after RAP treatment (Fig 4B).

To confirm that any phenotypic effect observed upon conditional truncation of DPAP3 is due to the loss of DPAP3 activity, A1cKO and F8cKO parasites were transfected with plasmid expressing WT or MUT DPAP3-HA under the control of the *dpap3* or *ama1* promoters (S6A Fig), resulting in the following complementation lines: F8cKO+WT_dpap3_, F8cKO+MUT_dpap3_, F8cKO+WT_ama1_, F8cKO+MUT_ama1_, A1cKO+WT_dpap3_, A1cKO+WT_ama1_, and A1cKO+MUT_ama1_. All complementation lines grew normally before RAP treatment and showed no apparent delay in parasite development.

IFA analysis confirmed colocalization between chromosomal DPAP3-mCh and episomal DPAP3-HA (Fig 5B and S6C Fig). Episomal expression was only high enough to be detected by IFA in 60–80% of schizonts, but was independent of RAP treatment (Fig 5C). This is probably due to different levels of episomal expression and plasmid segregation in schizonts. Efficient, but not complete, truncation of DPAP3-mCh was observed in all our complementation lines (Fig 5A). Labelling of DPAP3 with FY01 in parasite lysates from these lines show clear labelling of chromosomal DPAP3-mCh and episomal WT DPAP3-HA but not MUT DPAP3-HA (Fig 5D). As expected, RAP treatment results in a decrease of labelling of chromosomal DPAP3-mCh but not of episomal WT DPAP3-HA.

### Conditional knock out of DPAP3 reduces viability of blood stage parasites

**Fig 6:**
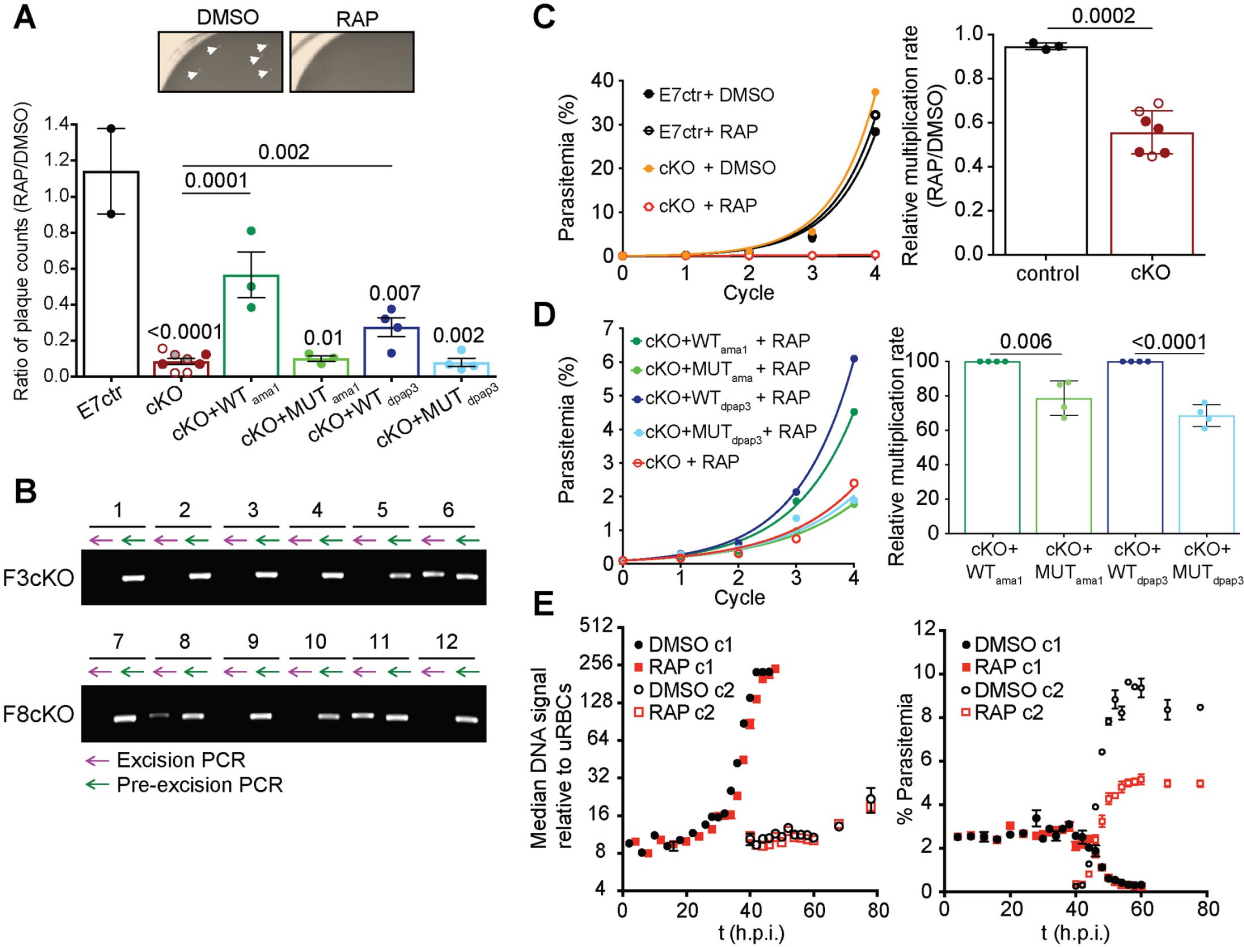
Conditional knock-out of DPAP3 leads to a severe growth defect. (**A**) Evaluation of parasite growth by plaque assay. Our different parasite lines were treated with DMSO or RAP and grown in flat bottom 96-well plates at 10 iRBC/well, and the number of plaques counted after 10–14 days (S1 Table). The ratio between RAP and DMSO treated parasites is plotted in the bar graph. Representative images of two wells from DMSO or RAP treatment of F8cKO parasites are shown (plaques are indicated by arrows). (**B**) Plaques originating from RAP-treatment of cKO lines contain non-excised parasites. Parasites from 12 wells that only contained a single plaque after RAP treatment of F3cKO and F8cKO parasites were expanded, and their genomic DNA analyzed for excision (purple arrow) and non-excision (green arrow) by PCR. (**C-D**) Effect of DPAP3 cKO on parasite proliferation. The growth of DMSO- or RAP-treated E7ctr and DPAP3cKO lines was monitored by FACS for four cycles. Cumulative percentage parasitemia was fitted to an exponential growth model. Representative growth curves for F8cKO and E7ctr parasites are shown. The bar graph shows the effect of RAP treatment on the culture multiplication rate per cycle relative to DMSO treatment. (**D**) Representative parasite proliferation curves obtained after RAP treatment of F8cKO and its complementation lines. Bar graph compares the multiplication rate after RAP treatment of DPAP3cKO lines complemented with WT or MUT DPAP3. (**A**, **C** and **D**) Each circle indicates a biological replicate: filled, F8cKO and its complementation lines; empty, A1cKO; grey, F3cKO. Error bars represent standard deviations. Only student’s t test significant values are shown. (**E**) Effect of DPAP3 KO on parasite development. Tightly synchronized A1cKO parasites pre-treated with DMSO or RAP were monitored over 76 h by FACS based on DNA content (Hoechst staining). Left Graph: No differences in DNA content was observed between DMSO and RAP treatment. Right Graph: The time-dependent decrease of parasites belonging to the 1^st^ cycle (c1) after DMSO or RAP treatment coincides with an increase of a parasite population belonging to 2^nd^ cycle (c2), thus effectively monitoring egress and invasion. Results are the mean ± standard deviation of three technical replicates.

To measure the effect of DPAP3 truncation on parasite proliferation, we used the recently published plaque assay[16] where the wells of a 96-well flat bottom plate containing a thin layer of blood were seeded with ~10 iRBCs/well. After 10–14 days, microscopic plaques resulting from RBC lysis can be detected with an inverted microscope. RAP treatment of our cKO lines resulted in 90% less plaques, an effect that could be partially rescued through episomal complementation with WT but not MUT DPAP3 (Fig 6A, S1 Table). After RAP treatment of the F3cKO and F8cKO lines, some wells contained a single plaque, suggesting that only one clonal parasite population grew in these wells. Parasites present in 12 of these wells were propagated and *dpap3* excision checked by PCR. All contained non-excised parasites but *dpap3* excision was detected in three cultures (Fig 6B). The presence of excised parasites in some of the wells suggests that DPAP3KO parasites replicate less efficiently and might not have had enough time to form a visible plaque within the 14 days of the assay. That said, the presence of non-excised parasites in all samples indicates that WT parasites quickly outcompeted DPAP3KO ones.

To test this hypothesis, we perform standard parasite multiplication assays after RAP or DMSO treatment. To prevent parasite overgrowth, cultures were diluted 10-fold in fresh blood and media whenever parasitemia reached 5%. RAP treatment of DPAP3cKO parasites results in a 10- to 15-fold decrease in parasitemia after 3–4 cycles, corresponding to an overall 50% decrease in multiplication rate per cycle compared to DMSO treatment (Fig 6C). However, these values underestimated the importance of DPAP3 on parasite replication since 5 cycles after RAP treatment, 60% of iRBC are non-excised parasites expressing DPAP3-mCh (S7A Fig). This result proves that the small fraction of non-excised parasites quickly outcompetes the excised ones. After RAP treatment, parasites complemented with WT DPAP3 grew significantly faster than those complemented with MUT DPAP3 (Fig 6D). Importantly, our multiple attempts to clone DPAP3KO parasites after RAP treatment failed, indicating that DPAP3 activity is required for parasite proliferation under our culturing conditions.

To determine which point of the erythrocytic cycle is disrupted by the loss of DPAP3, a tightly synchronized culture of A1cKO parasites at ring stage was treated with DMSO or RAP for 3 h, and the culture monitored for the following 80 h. Samples were collected every 2–4 h, fixed, stained with Hoechst, and analyzed by FACS. No significant difference in DNA staining was observed between WT and KO parasites, suggesting that DPAP3 is not required for intracellular development (Fig 6E, left graph). Quantification of iRBCs belonging to the first or second cycle after treatment shows that DPAP3KO parasites egress at the same time as WT but produce **~**50% less rings (Fig 6E, right graph). This suggests that DPAP3 is only important for RBC invasion, which is in direct contradiction with its previously suggested role in egress[18].

### PfDPAP3 does not play a role in parasite egress

Despite being expressed early during schizogony (Fig 1F), we did not observe any delay in parasite development between 36–48 h.p.i. (Fig 6E). This was confirmed by IFA by counting the number of mature schizonts at the end of the cycle after RAP treatment. Apical localization of SUB1 to the exonemes was used as a marker for schizont maturity. No significant difference was observed between DMSO and RAP treatment of cKO or complementation lines (S7B Fig). Importantly, proper localization of MSP1, SUB1, AMA1, EBA175, RON4, and RopH2 was observed in all RAP-treated cKO lines (Fig 4D and S7C and S7D Fig).

**Fig 7:**
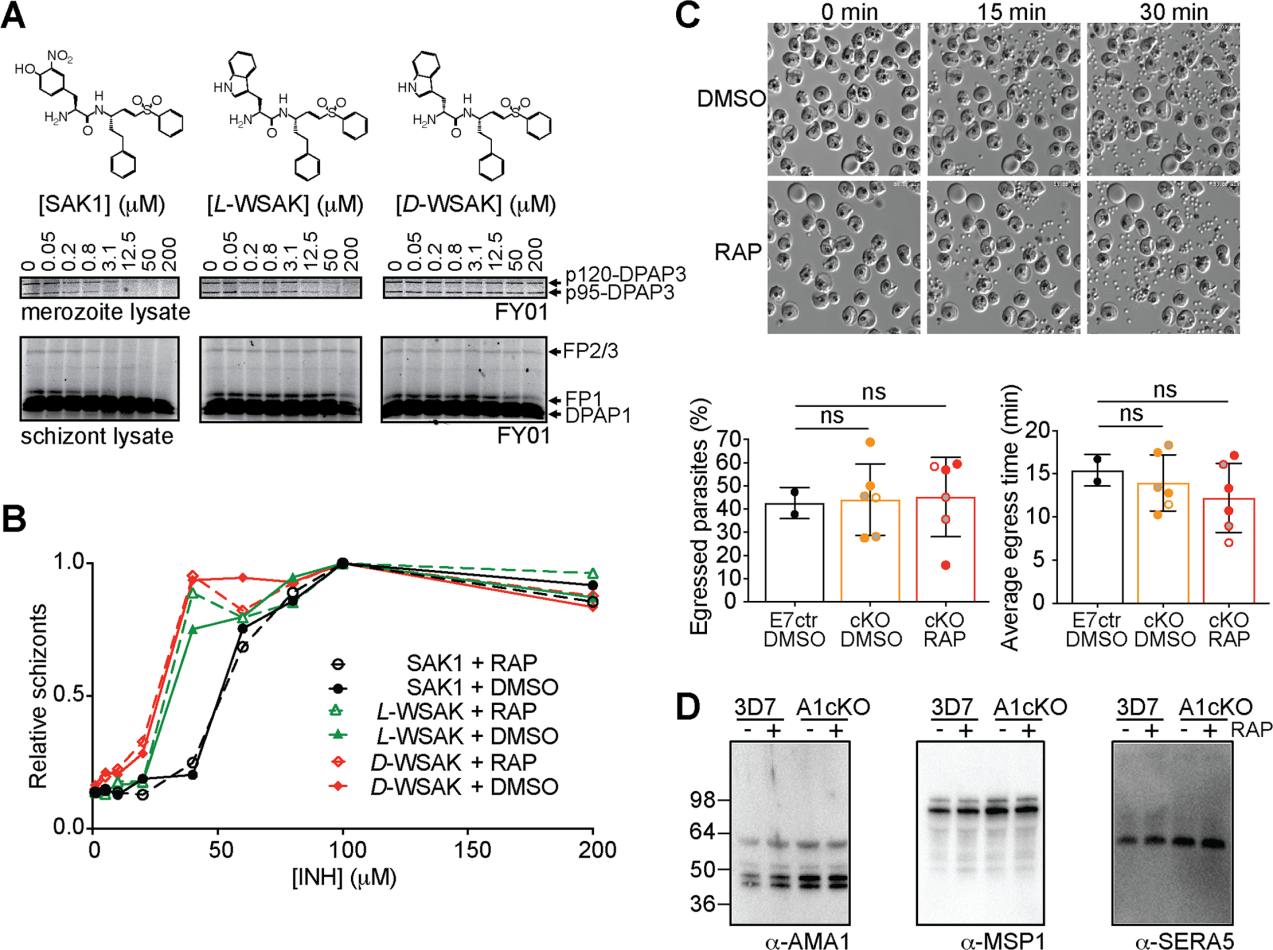
Role of DPAP3 in parasite egress. (**A**) *L*-WSAK is a more selective DPAP3 inhibitor than SAK1. The structures of SAK1, *L-*WSAK and *D*-WSAK are shown. Merozoite or schizont lysates were pre-incubated with a dose response of inhibitor for 30 min followed by FY01 labelling. Samples were run on an SDS-PAGE gel, and the gel scanned on a flatbed fluorescence scanner. Bands corresponding to each of the labelled cysteine proteases are indicated by arrows. (**B**) Effect of inhibitors on egress. DMSO or RAP treated A1cKO parasites were treated at schizont stage with a dose response of inhibitor for 24 h. The accumulation of schizonts upon inhibitor treatment was quantified by FACS. (**C**) Analysis of egress by video microscopy. C2-arrested schizonts obtained from DMSO- or RAP-treated of A1cKO, F8cKO, F3cKO or E7ctr parasite lines were monitored by time-lapse DIC microscopy for 30 min after C2 washout. Representative still images taken at 0, 15, and 30 min are shown for F8cKO parasites. The full time-lapse video can be seen in S4 Video. The percentage of schizonts that egressed during this 30 min time-lapse (left graph) and the time at which each individual schizont ruptured (right graph) are shown. Bar graphs show mean values ± standard deviation; circles show individual biological replicates (filled for F8cKO, empty for A1cKO, and grey for F3cKO). (**D**) WB analysis of culture supernatant collected after egress of 3D7 and A1cKO after DMSO or RAP treatment. No differences in the processing of AMA1, MSP1 or SERA5 was observed as a result of DPAP3 truncation.

Previously published work using the SAK1 inhibitor showed arrest of egress upstream of SUB1 activation[18]. This result suggested that DPAP3 might be important for parasite egress. Although we have been able to reproduce these results, we show that SAK1 treatment of schizonts 6 h before egress arrests schizogony upstream of SUB1 and AMA1 expression rather than parasite egress (S8 Fig). This explains why no SUB1 or AMA1 could be detected by WB in the previous study[18]. In addition to SAK1, we also synthesized a more selective DPAP3 inhibitor by replacing the nitro-tyrosine N-terminal residue of SAK1 with *L-*Trp (*L*-WSAK) and its diastereomer negative control containing *D-*Trp (*D-*WSAK). To test the specificity of these inhibitors, merozoite or schizont lysates were pre-incubated with a dose response of compound followed by 1 h labelling with FY01 (Fig 7A). Although SAK1 blocks labelling of DPAP3 at lower concentration than *L-*WSAK, it also inhibits all the falcipains (FP1, FP2, and FP3) above 5 μM. *L-*WSAK only inhibits other targets above 200 μM. As expected, the *D-*WSAK control compound is unable to inhibit any of the labelled cysteine proteases and is at least 100-fold less potent than *L-*WSAK at inhibiting DPAP3 (Fig 7A). We then compared the effect of these inhibitors in parasite egress on the A1cKO line. Surprisingly, all compounds blocked egress independently of RAP treatment (Fig 7B), and no difference in potency between *L-* and *D-*WSAK was observed. These results prove that these vinyl sulfone compounds do not act through inhibition of DPAP3 but rather through off-target or toxicity effects.

As a final proof to show that DPAP3 is not involved in parasite egress, we arrested DMSO or RAP treated DPAP3cKO schizonts with C2 and monitored egress by live microscopy after removal of the PKG inhibitor. Analysis of these videos showed no significant difference in the number of schizonts that ruptured, nor on how fast merozoites egressed after C2 wash out (Fig 7C and S4 Video). Also, we could not detect differences in the levels and/or processing of AMA1[38] or SUB1 substrates (SERA5[10] and MSP1[13]) between DMSO and RAP treated parasites (Fig 7D). These findings together with the lack of colocalization between DPAP3 and SUB1 (Fig 1H and S3 Fig) clearly demonstrate that DPAP3 is not responsible for proper processing and activation of SUB1, and that DPAP3 does not play a role in egress. However, its localization in an apical secretory organelle (Figs 1 and 2) and the time-course analysis of the A1cKO line (Fig 6E) strongly suggest a function in RBC invasion.

### PfDPAP3 is important for RBCs invasion

**Fig 8:**
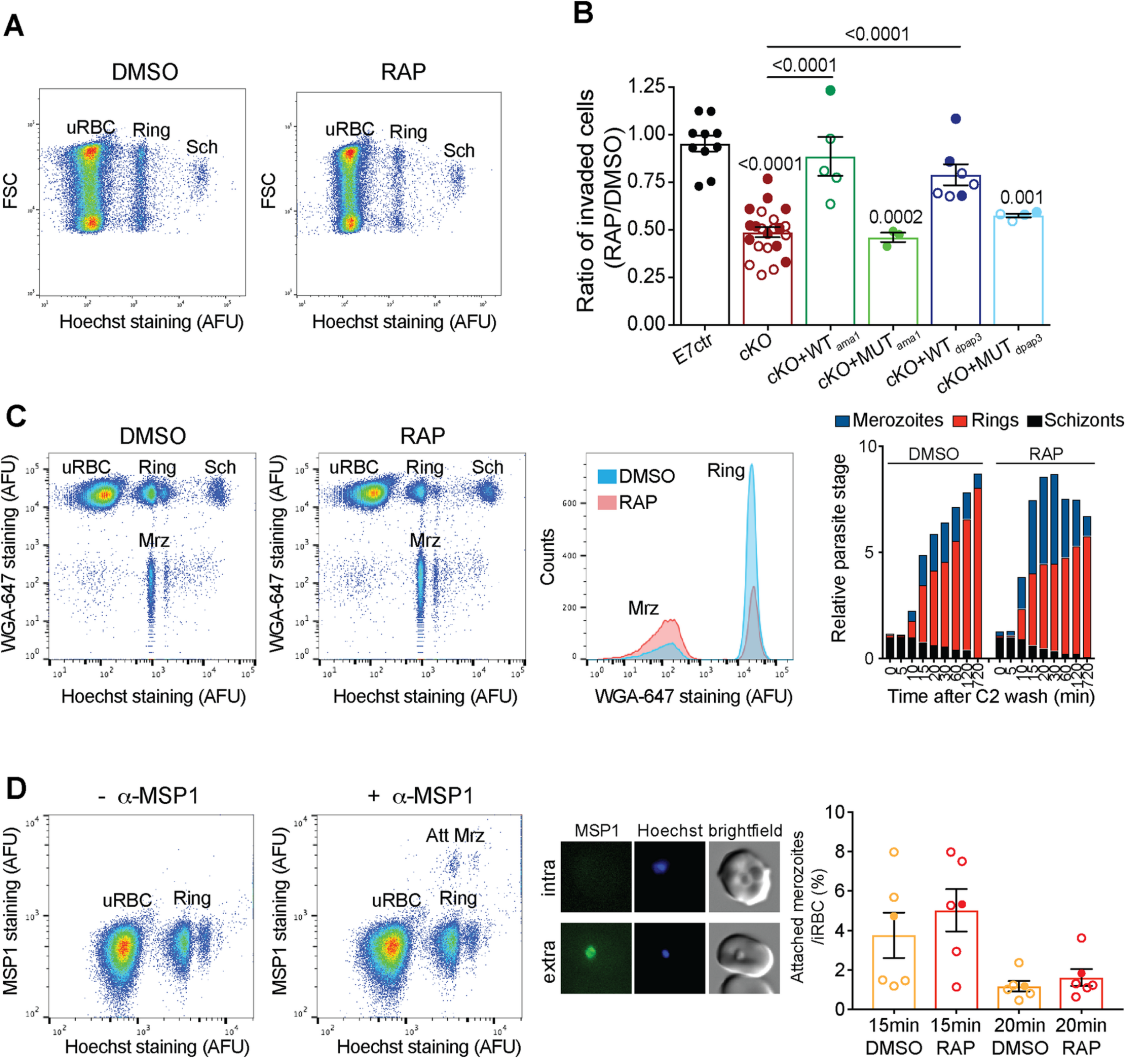
Role of DPAP3 in RBC invasion. (**A**) Representative FACS plot (forward light scattering vs. Hoechst staining) showing a decrease in invasion of the A1cKO upon RAP treatment. The populations of uRBCs, rings, and schizonts (Sch) are indicated. (**B**) Analysis of invasion efficiency of DPAP3 cKO and complementation lines. Schizonts collected 45 h after DMSO or RAP treatment were incubated with fresh erythrocytes for 8–14 h, fixed, stained with Hoechst, and analyzed by FACS. Shown is the ratio in invasion efficiency between RAP- and DMSO-treated parasites. Filled and empty circles represent individual biological replicates for the F8cKO and A1cKO, respectively and their corresponding complementation lines. Student’s t test significance values between cell lines are shown above the lines, or above each bar when comparing to the E7ctr. Only significant p-values are shown. (**C**) FACS analysis of extracellular merozoites. C2-arrested A1cKO schizonts pretreated with DMSO or RAP were incubated with fresh RBCs after C2 removal. Samples were collected at the indicated time points, fixed, and stained with Hoechst and WGA-Alexa647. The FACS plot and histogram show samples collected 20 min after C2 washout. Free merozoites (Mrz) show positive staining for DNA but negative for WGA-Alexa647. Quantification of the different parasite stage populations over time is shown on the bar graph; biological replicates are shown in S9 Fig. (**D**) Quantification of attached merozoites by flow cytometry. Samples collected 15 and 20 min after C2 washout during invasion assays (performed as in **C**), were stained with Hoechst and anti-MSP1 antibody (anti-mouse Alexa488 as secondary antibody). Because MSP1 is shed during invasion, merozoites attached to the RBCM (Att Mrz) can be differentiated from intracellular parasites as the cell population positive for DNA and MSP1 staining. FACS plots compare anti-mouse Alexa488 staining in samples treated with or without the anti-MSP1 antibody. MSP1 staining (green) of attached merozoites under these conditions was confirmed by microscopy (central panel). Quantification of the population of attached merozoites relative to the ring population is shown on the bar graph. Circles represent different biological replicates: filled for F8cKO and empty for A1cKO. No significant difference was observed between DMSO and RAP treatment.

Mature schizonts obtained after DMSO or RAP treatment of our different parasite lines were incubated with fresh RBCs for 8–14 h, fixed, and the population of schizonts and rings quantified by FACS (Fig 8A). On average, we observed a 50% reduction in the number of iRBCs after RAP treatment of our cKO lines. This invasion defect could be rescued by episomal expression of WT but not MUT DPAP3 independently of the promoter used (*ama1* or *dpap3*), thus indicating that DPAP3 activity is important for RBC invasion (Fig 8B).

To determine which invasion step is impaired by the loss of DPAP3, DMSO- or RAP-treated cKO parasites were arrested at schizont stage with C2, incubated with fresh RBCs after C2 washout, and samples collected at different time points for FACS analysis. Fluorescent wheat germ agglutinin (WGA-Alexa647) binds to lectins on the RBC surface and when combined with Hoechst staining allows us to differentiate free merozoites from iRBCs (Fig 8C). Quantification of the different parasite stage populations over time clearly shows a decrease in the number of rings upon RAP treatment, with an inversely proportional increase in the number of free merozoites (Fig 8C and S9A Fig).

The RBC population showing similar levels of DNA signal as free merozoites is mainly composed of ring stage parasites but likely contains a small proportion of extracellular merozoites tightly attached to the surface of the erythrocyte. These two populations were differentiated by staining the samples with a monoclonal MSP1 antibody (m89.1), whose epitope is within the portion of MSP1 that is shed during invasion (Fig 8D). Using this assay, we did not observe a significant difference in the number of attached merozoites relative to the number of rings between DMSO and RAP treatment of our cKO lines. This suggests that after DPAP3KO parasites tightly attach to the RBC surface they invade as efficiently as WT parasites. Therefore, DPAP3 is likely important for the initial recognition of and attachment to RBCs.

## Discussion

This study provides the first characterization of the biological function of DPAP3 in parasite development. We have shown that DPAP3 is important for efficient RBC invasion and parasite proliferation. DPAP3 is expressed early during schizogony, it localizes to small apical organelles that are closely associated with the neck of the rhoptries, and it is secreted at the time of PVM breakdown but before parasite egress. We have also demonstrated that DPAP3 has dipeptidyl aminopeptidase activity but contrary to other DPAPs, removal of the prodomain is not required for activation. Our cKO and complementation studies provide strong evidence that DPAP3 activity is only required for efficient RBC invasion, but not for intracellular parasite development or parasite egress. Importantly, we have proven that the block in egress phenotype previously reported using vinyl sulfone inhibitors is not due to DPAP3 inhibition but rather to off-target or toxicity defects. Two cysteine proteases have been shown to play an important role in egress: human calpain-1 [39] and SERA6 [17]. It is therefore possible that the block in egress phenotype might be due to inhibition of either one of these two proteases. This study illustrates the importance of using negative control compounds when trying to associate a specific phenotype to the inhibition of a particular target, as well as the need to genetically validate functional information obtained through chemical methods [6]. Interestingly, in a recently published study, the Koning-Ward lab used the *glmS* riboswitch system to conditionally knockdown DPAP3 in *P*. *falciparum* [40]. However, they did not observe any significant effect in parasite proliferation, nor in egress or RBC invasion. We think that this is likely due to the fact that they only achieved partial knockdown of DPAP3, and that the residual level of DPAP3 activity was sufficient to perform its function. That said, their localization studies are consistent with ours and confirm that DPAP3 resides in novel apical secretory organelles, and that it does not colocalize with rhoptry, microneme, or dense granule markers.

Our initial characterization of the invasion defect associated with the loss of DPAP3 suggests that this protease might play a role in the attachment of merozoites to the RBC surface. Indeed, in our invasion assays we observed a significant increase in the number of free merozoites upon cKO of DPAP3. Moreover, while DPAP3 KO results in a significant decrease in the number of rings, the ratio between merozoites attached to the RBC surface and those that have successfully invaded RBCs is the same between WT and KO parasites, suggesting that the decrease in invasion efficiency is likely upstream of merozoite attachment and tight junction formation, and that DPAP3 is likely important for the initial recognition of RBCs. We think it is unlikely that DPAP3KO parasites might be less efficient at forming a tight junction since we predict that such a defect would result in an increase of attached parasites relative to the total number of invaded RBCs. However, this is a possibility that we cannot completely rule out at this moment.

Interestingly, we observed a wide variation in the decrease of invasion efficiency upon cKO of DPAP3, ranging from 25 to 75% inhibition. This variation is much larger than the experimental variation observed with our E7 control line, and it is likely due to heterogeneity among the different batches of blood used to perform invasion assays. This observation suggests that DPAP3 activity might be important to recognize host cell receptors that are differentially expressed in the human population, and is consistent with our propose role of DPAP3 in RBC attachment.

The facts that DPAP3 is expressed early during schizogony, that it localizes in secretory apical organelles, and that it is active under mild acidic conditions (pH 5–7, maximum activity at pH 6), suggest that DPAP3 might process its substrates within the apical organelle where it resides. However, at this stage we cannot discard the possibility that DPAP3 might process its substrates at neutral pH, either during trafficking (in the ER or Golgi), extracellularly after secretion, or even during secretion of proteins from apical organelles. Micronemes are trafficked to the merozoite apex underneath the inner membrane complex before releasing their protein cargo, thus coming in very close proximity to the neck of the rhoptries [41,42]. It is therefore possible that DPAP3 might interact with proteins of other secretory organelles as they are secreted, similarly to how micronemal CyRPA (cysteine rich protective antigen) and Ripr (Rh5 interacting protein) come together with rhoptry Rh5 (reticulocyte binding protein homologue 5) at the merozoite apex after egress [43]. Finally, we think it is unlikely that DPAP3 acts extracellularly on RBC surface proteins because co-culturing equal amounts of WT and DPAP3KO parasites did not rescued the DPAP3KO invasion defect (S9B Fig).

It is difficult to speculate about the nature of DPAP3 substrates since we could not colocalize this protease with any of the tested apical markers. However, its substrates are likely to be proteins in the secretory pathway that are directly or indirectly important for invasion. Also, given that DPAPs cleave N-terminal dipeptides from protein substrates, DPAP3 likely recognizes the N-terminus of its substrates after they have been cleaved by another protease. Most proteins secreted into the PV or onto the merozoite surface are processed during traffic or after secretion, thus exposing one or multiple N-termini that might be potential DPAP3 substrates. For example, DPAP3 might trim the N-terminus of its substrates after signal peptide removal, thus potentially affecting their localization or stability. Indeed, the N-terminal sequence downstream of the signal peptide has been shown to be important for proper localization of rhoptry and micronemal proteins both in *P*. *falciparum* [44,45] and *T*. *gondii* [46,47]. Interestingly, two recent studies have shown that the aspartyl protease plasmepsin IX (PMIX) is essential for RBC invasion and that it acts as a maturase of rhoptry proteins[48,49]. In both studies, PMIX was shown to localize at the apical end of merozoites either within or, similarly to DPAP3, in close proximity to the rhoptries. This similar localization raises the possibility that DPAP3 might trim the N-terminus of rhoptry proteins after being processed by PMIX. Finally, most surface proteins that are involved in RBC invasion (EBAs, Rhs, MSPs, AMA1, RON2, etc) expose one or more extracellular N-termini[7,50], and the N-terminal domains of some of these proteins have been shown to be important in mediating protein-protein interactions and biological function. Interestingly, in *T*. *gondii* extracellular trimming of the N-terminus of surface proteins has been well documented [51,52]. *T*. *gondii* also expresses DPAPs in secretory organelles[53], and a recent proteomic study has shown evidence that the N-terminus of certain secreted proteins, such as TgSUB1 and TgMIC11, is trimmed through the removal of dipeptides[54]. It is therefore possible that DPAP3-mediated trimming of the N-terminus of certain parasite surface proteins might modulate their affinity towards host cell receptors.

Our biochemical studies have shown that DPAP3 is an unusual papain-fold protease since removal of its prodomain is not required for activation. Most proteases prodomains act as endogenous inhibitors and internal chaperones. Although the prodomain of DPAP3 might be required for proper folding of this large protein (941aa), we cannot discard the possibility that it might have other biochemical functions, such as recognizing substrates or binding to cofactors that modulate DPAP3 activity. Interestingly, the pro-form of *P*. *falciparum* DPAP1 has been shown to localize to the PV in mature schizonts[30], and processing of recombinant DPAP1 from its ‘zymogen’ form to is fully processed form only increases its activity 2–3 fold [32], suggesting that similarly to DPAP3, the ‘zymogen’ form of DPAP1 is active and might be able to process the N-terminus of PV or merozoite surface proteins. Therefore, both DPAP1 and DPAP3 are secreted before egress, raising the possibility that they might play redundant or complementary functions during RBC invasion.

## Methods

Synthesis of inhibitors and production of DPAP3 antibodies are described in S1 Text. Primers and antibodies used in this study are listed in S2 Table and S3 Table, respectively.

### Design of expression constructs

Three synthetic genes codon-optimized for insect cells were synthesized by Genewiz and cloned into the puc57 vector backbone: puc57-rDPAP3-Nt, puc57-rDPAP3-Ct-wt, and puc-rDPAP3-Ct-mut. The first one codes for the N-terminal portion of DPAP3 (Met1-Asp469) and the other two for the C-terminal portion (Lys455-Stop941) containing the catalytic domain of DPAP3 and harboring either the catalytic cysteine Cys504 or the C504S inactivating mutation. All synthetic sequences contained a C-terminal His_6_-tag and were flanked with the BamHI and HindIII restriction sites at the 5’ and 3’ end, respectively. A ClaI restriction site is present in the 45 bp overlapping sequence (Lys455-Asp469) between puc57-rDPAP3-Nt and puc57-rDPAP3-Ct-wt/mut. Digestion of these plasmids with BamHI, HindIII, and ClaI, followed by ligation of the N- and C-terminal products into the puc57 backbone yielded puc57-rDPAP3-WT and puc57-rDPAP3-MUT. The BamHI and HindIII restriction sites were used to clone full length *dpap3* into the pFastBacHT vector (Thermo Fisher Scientific) for expression of WT or MUT DPAP3 (pFB-rDPAP3wt and pFB-rDPAP3mut) in insect cells.

### Recombinant expression of DPAP3

rDPAP3 was expressed in Sf9 insect cells using the baculovirus system. *E*. *coli* DH10Bac cells (Invitrogen) were transformed with pFB-rDPAP3wt and pFB-rDPAP3mut following the manufacturer recommendation. Baculovirus DNA was extracted using the BACMAX DNA purification kit (Epicentre) and transfected into a 5 mL culture of Sf9 cells (2x10^6^ cells/mL) using Cellfectin (Thermofisher). After 3 days, the culture supernatant containing baculovirus particles was collected (P1 stock). To increase the viral load of our stocks, 1 mL of culture supernatant was serially passage twice into 25 mL of Sf9 cultures at 2x10^6^ cell/mL for 3 days to obtain P2 and P3 viral stocks, which were stored at 4°C or frozen in liquid N_2_ in the presence of 10% glycerol. Insect cells were grown in SF-900-II serum free medium (Gibco) at 27°C under shaking conditions.

For rDPAP3 expression, Sf9 cells at 2x10^6^ cells/mL were infected with 0.4 mL of P3 viral stock per liter of culture. The supernatant containing rDPAP3 was collected 72 h after infection, supplemented with protease inhibitors (1 mM PMSF, 0.5 mM EDTA, 1 μM pepstatin, 1 μM bestatin, and 10 μM E64), and its pH adjusted by adding 50 mM TrisHCl from a 1 M solution at pH 8.2; 10% glycerol was added before storage at -80°C. Note that despite being a general Cys protease inhibitor, E64 does not inhibit DPAP3.

A three steps purification consisting of ion exchange, affinity, and size exclusion chromatography was used to purify rDPAP3. First, culture supernatant was passed 3 times through 0.05 volumes of Q-sepharose (GE Healthcare) pre-equilibrated with Buffer A (50 mM Tris pH 8.2 containing the above-mentioned protease inhibitors). The resin was washed with 5 volumes of Buffer A and 2.5 volumes of 50 mM NaCl in Buffer A, and rDPAP3 eluted with 400 mM NaCl in Buffer A. Fractions containing rDPAP3 were pooled, diluted 1:1 into Buffer B (100 mM sodium acetate, 100 mM NaCl, pH6, and the protease inhibitors mentioned above), and passed through 0.05 volumes of Ni-NTA resin (Qiagen). The resin was washed with 10 volumes of Buffer B, and rDPAP3 eluted by lowering the pH of Buffer B to 5. Fractions containing rDPAP3 were pooled, concentrated using a Centricon Plus-70 Centrifugal Filter Unit (Millipore), loaded on a Superdex 200 10/300 GL size exclusion column, and run on an AKTA FPLC with Buffer A. Fractions containing rDPAP3 were pooled, concentrated, and stored at -80°C in the presence of 10% glycerol.

### DPAP3 activity assay using fluorogenic substrates or FY01

DPAP3 activity was measured either using the DPAP fluorogenic substrates VR-ACC[32] or FR-βNA (Sigma), or with the FY01 activity-based probe. When using FY01, samples (intact parasites, parasite lysates, insect cell supernatant, or rDPAP3 purification fractions) were labelled with 1 μM FY01 for 1 h, boiled in loading buffer, run on a SDS-PAGE gel, and the fluorescence signal measured on a PharosFX (Biorad) flatbed fluorescence scanner [18]. To determine the potency and specificity of inhibitors against DPAPs and the falcipains, parasite lysates diluted in acetate buffer (50 mM sodium acetate, 5 mM MgCl_2_, 5 mM DTT, pH 5.5) were pretreated for 30 min with a dose response of inhibitor followed by FY01 labelling.

When using VR-ACC (10 μM) or FR-βNA (100 μM), substrate turnover was measured on a M5e Spectramax plate reader (λ_Bex_ = 355 nm/λ_em_ = 460 nm or λ_ex_ = 315 nm/λ_em_ = 430 nm, respectively) in 50 mM sodium acetate, 20 mM NaCl, 5 mM DTT, and 5 mM MgCl_2_, pH5.5. The pH dependence of rDPAP3 was determined at 10 μM VR-ACC using a 20 mM sodium acetate, 20 mM MES and 40 mM TRIS triple buffer system containing 5 mM DTT, 0.1% CHAPS, 20 mM NaCl, and 5 mM MgCl_2_.

### Design of targeting and complementation constructs

All constructs designed to integrate into the *dpap3* locus by single-crossover recombination (tagged or cKO lines) contained either a 1065bp C-terminal homology region fused to GFP, or a 1210bp homology region upstream of the catalytic Cys504 (Asp39-Glu392) fused to a recodonized C-terminal region (Lys393-Stop941) tagged with mCherry or triple HA tag (HA_3_). The recodonized region contained either WT Cys504, or the C504S mutation. The construct designed to generate the DPAP3-HA, DPAP3-mCh, and DPAP3-GFP tagged lines we obtained as shown in S10A and S10B Fig. Briefly, the *dpap3* C-terminal homology region was inserted into the pPM2GT plasmid to generate pPM2GT-DPAP3Ct-GFP. The N-terminal region of *dpap3* in the pFB-rDPAP3-wt/mut vector was replaced with the homology region to generate pFB-chDPAP3-wt/mut. The *dpap3* ORF was then introduced into the pHH1-SERA5ΔCt-HA—obtained after removal of the C-terminal part of SERA5 in the pHH1-SERA5-loxP-DS_PbDT3’[33]—resulting in plasmids pHH1-chDPAP3-wt/mut-HA, which harbor a C-terminal HA_3_ tag and a *loxP* site downstream of the Pb3’ UTR. The HA_3_ sequence of pHH1-SERA5ΔCt-HA was replaced with mCherry—amplified from the pREST-B plasmid[55]—to generate the pHH1-SERA5ΔCt-mCh, and the ORF of WT or MUT DPAP3 introduced into this plasmid to generate the pHH1-chDPAP3-wt/mut-mCh constructs.

To conditionally truncate *dpap3* we used two different approaches. The first approach introduced a *loxP* site within the ORF of *dpap3*, in an Asn-rich region (Asn414-Asn444) upstream of the catalytic domain, resulting in replacement of Asn430-Asp434 with a *loxP* coding peptide (ITSYSIHYTKLFTG). To make the pHH1-chDPAP3_loxP-mCh construct (S10C Fig), the N- and C-terminal portions of DPAP3 were amplified from the pHH1-chDPAP3-wt-mCh, which contains a 3’UTR *loxP* site, and ligated into the plasmid backbone. A *loxP* site was introduced in the backward primer used to amplified the N-terminal region. The second approach inserted a *loxPint*[36] between the homology and recodonized regions. A synthetic 1600bp sequence (GeneWiz) containing the *loxPint* fragment flanked by targeting sequences was introduced into construct pHH1-chDPAP3-wt-mCh to generate the pHH1-chDPAP3_loxPint-mCh plasmid.

Finally, for episomal complementation of the cKO lines, plasmids pHH1-chDPAP3-wt/mut-HA (S10A Fig) were modified in order to express full-length WT or MUT DPAP3 under the control of the *dpap3* or *ama1* promoters. Firstly, to select for parasites containing the complementation plasmids after transfection, the puromycin N-acetyltransferase (pac) gene, which confers resistance to puromycin, was amplified from mPAC-TK (a kind gift of Alex Maier) and subsequently ligated into pHH1-chDPAP3-wt/mut-HA plasmids. The homology region of this plasmid was replaced with a recodonized N-terminal *dpap3* amplified from puc57-rDPAP3-Nt, resulting in pHH1-rDPAP3-wt/mut-HA plasmids (S10D Fig). The *dpap3* and *ama1* promoters (970 and 1456bp upstream of the start codon, respectively) were amplified from genomic DNA and ligated into these plasmids to generate the pHH1-*dpap3-*rDPAP3-wt/mut-HA, and pHH1-*ama1-*rDPAP3-wt/mut-HA complementation constructs.

All final construct sequences were verified by nucleotide sequencing on both strands. Primers for PCR amplification and restriction sites used to generate these plasmids are listed in S2 Table and indicated in S10 Fig, respectively.

### Generation and maintenance of transgenic parasite lines

All constructs were transfected at schizont stage using a 4D-Nucleofector electroporator (Lonza) as previously described[12]. DPAP3-tagged and DPAP3-cKO lines were obtained through multiple on and off drug selection cycles with WR99210 (Jacobus Pharmaceuticals) and cloned by limited dilution as previously described[56]. To generate the DPAP3-GFP, DPAP3-HA and DPAP3-mCh lines, pPM2GT-DPAP3Ct-GFP, pHH1-chDPAP3-wt-HA and pHH1-chDPAP3-wt-mCh were transfected into *P*. *falciparum* 3D7 parasites. Plasmid pHH1-chDPAP3-mut-mCh was transfected multiple times into 3D7 in an attempt to swap the endogenous catalytic domain of DPAP3 with one containing the inactivating C504S mutation, but no integration was observed even after five drug cycles. The A1cKO and F3cKO & F8cKO lines were obtained after transfection of pHH1-chDPAP3_loxP-mCh and pHH1-chDPAP3_loxPint-mCh, respectively, into *P*. *falciparum* 1G5 parasites that endogenously expressed DiCre. Finally, the E7ctr line containing only the 3’UTR *loxP* site was generated by transfecting 1G5 parasites with pHH1-chDPAP3-wt-mCh. Complementation lines were obtained after transfection of A1cKO or F8cKO with pHH1-*dpap3-*rDPAP3-wt-HA, pHH1-*dpap3-*rDPAP3-mut-HA, pHH1-*ama1-*rDPAP3-wt-HA, or pHH1-*ama1-*rDPAP3-wt-HA and selection with WR99210 and puromycin. All parasite lines were maintained in RPMI 1640 medium with Albumax (Invitrogen) containing WR99210 (plus puromycin for the complementation lines) and synchronized using standard procedures[57].

To conditionally truncate the catalytic domain of DPAP3, tightly synchronized ring-stage parasites were treated for 3–4 h with 100 nM RAP (Sigma) or DMSO at 37°C, washed with RPMI, and returned to culture. Schizonts purified at the end of the cycle were used to determine the excision efficiency at the DNA (PCR) or protein (IFA, WB) level.

### Biochemical fractionation to study DPAP3 secretion

To determine exactly when DPAP3 is secreted in relation to PVM and RBCM breakdown, 20 μL of purified schizonts in 4 mL of RPMI were arrested with C2 or E64, or were allowed to egress for 1 h in the presence of 1 μM FY01. These cultures were centrifuged at 3000 rpm to separate intact schizonts from free merozoites and the culture supernatant. Merozoites were isolated from the culture supernatant by centrifugation (10 min at 13000 rpm), and proteins in the culture supernatant precipitated with 10 volumes of ice cold methanol and overnight incubation at -80°C. The schizont fractions were treated with 30 μL of 0.15% saponin in PBS to lyse the PVM and RBCM, and thus separate PV and host cytosolic components (saponin soluble fraction) from the parasite pellets (saponin insoluble fraction), which were washed once with PBS. Each fraction was then dissolved into PBS to a final volume of 50 μL, boiled for 10 min after adding 17 μL of 4X loading buffers. Equal volumes of each fraction (20 μL) were run in a SDS-PAGE gel under reducing conditions for WB and FY01 labelling analysis.

### Staining and microscopy

Thin films of *P*. *falciparum* cultures were air-dried, fixed in 4% (w/v) formaldehyde (PFA) for 20 min (Agar Scientific Ltd.), permeabilized for 10 min in 0.1% (w/v) Triton X100 and blocked overnight in 3% (w/v) bovine serum albumin (BSA) or 10% (w/v) goat serum (Invitrogen) in PBS. Slides were probed with monoclonal antibodies or polyclonal sera as described previously[58] (See S3 Table for antibodies used in this study), subsequently stained with Alexa488-, Alexa594-, Alexa647-labelled secondary antibodies (Molecular Probes) and DAPI (4,6-diamidino-2-phenylindole), and mounted in ProLong Gold Antifade (Molecular Probes). Images were collected using AxioVision 3.1 software on an Axioplan 2 Imaging system (Zeiss) using a Plan-APOCHROMAT 100x/1.4 oil immersion objective or LAS AF software on an SP5 confocal laser scanning microscope (Leica) using a HCX PL APO lamda blue 63x/1.4 oil immersion objective. Super-resolution microscopy was performed using a DeltaVision OMX 3D structured illumination (3D-SIM) microscope (Applied Precision). Images were analyzed with ImageJ (NIH), Adobe Photoshop CS4 (Adobe Systems) and Imaris x64 9.0.0 (Bitplane) software.

### Immunoelectron microscopy

For IEM, mature schizonts from the DPAP3-GFP and 3D7 control lines were concentrated using a magnetic activated cell sorting (MACS) LD separation column (Miltenyi Biotec). Briefly, iRBCs were loaded onto an LD column attached to Midi MACS pre-equilibrated with media. The column was washed twice with media and schizonts eluted with media after detaching the column from the magnet. Parasites were then fixed in 4% paraformaldehyde/0.1% glutaraldehyde (Polysciences) in 100 mM PIPES and 0.5 mM MgCl_2_, pH 7.2, for 1 h at 4°C. Samples were embedded in 10% gelatine and infiltrated overnight with 2.3 M sucrose/20% polyvinyl pyrrolidone in PIPES/MgCl_2_ at 4°C. Samples were trimmed, frozen in liquid nitrogen, and sectioned with a Leica Ultracut UCT cryo-ultramicrotome (Leica Microsystems). Sections of 70 nm were blocked with 5% FBS and 5% NGS for 30 min and subsequently incubated with rabbit anti-GFP antibody 6556 (Abcam) at 1:750 overnight at 4°C. Colloidal gold conjugated anti rabbit (12 nm) IgG (Jack Imm Res Lab) was used as secondary antibody.

### Plaque assays

Plaque assays were performed as previously described[16]. Briefly, the 60 internal wells of a flat-bottom 96 well plate were filled with 200μL of DMSO- or RAP-treated parasite culture at 10 iRBCs/well and 0.75% hematocrit, incubated for 12–14 days at 37°C, and the number of microscopic plaques counted using an inverted microscope.

### Flow cytometry-based replication, time course, and invasion assays

For all FACS-based assays, samples were fixed for 1 h at RT with 4% PFA and 0.02% glutaraldehyde, washed with PBS, and stored at 4°C. Samples were stained with SYBR Green (1:5000) or Hoechst (2 μg/mL), run on a FACScalibur or FortessaX20 flow cytometers (Becton-Dickinson Bioscience), and the data analyzed with CellQuest Pro or FlowJo.

For replication assays, cultures at 0.1% parasitemia (ring stage) and 2% hematocrit were grown for 4 cycles after DMSO or RAP treatment. Aliquots were fixed every 48 h at trophozoite stage, stained with SYBR Green, and parasitemia quantified by flow cytometry. To avoid parasite overgrowth, cultures were diluted 10-times whenever they reached 5% parasitemia. The cumulative percentage parasitemia (CP) over 4 cycles was fitted to an exponential growth model: CP = P_t0_^.^MR^N^, where P_t0_ is the initial parasitemia, MR the multiplication rate per cycle, and N the number of cycles after treatment.

To look at the effect of DPAP3 truncation on the full erythrocytic cycle, A1cKO parasites were synchronized within a 2 h window, treated with DMSO or RAP for 3 h, and put back in culture for 76 h. Sample were collected every 2–4 h, fixed, stained with Hoechst, and analyzed by flow cytometry. DNA levels were quantified as the median fluorescence signal of iRBC divided by the background signal for uninfected RBCs (uRBCs).

For standard invasion assays, schizonts purified from DMSO or RAP treated cultures were incubated with fresh RBCs for 8–14 h, fixed, and stained with Hoechst. The population of uRBC, rings and schizonts was quantified based on DNA content. The invasion rate was determined as the ratio between the final population of rings and the initial population of schizonts. Invasion time courses were performed by arresting purified schizonts with 1 μM C2 for 4 h, washing twice with warm media, and culturing with fresh RBCs under shaking conditions. Samples were collected at different time points, fixed, and split into two aliquots: One was stained with Hoechst and WGA-Alexa647 and run on a flow cytometer at a high forward scattering voltage in order to detect free merozoites. The populations of uRBCs, free merozoites, rings, and schizonts were quantified with FlowJo. The other aliquot was blocked with 3% BSA in PBS overnight at 4°C, and stained without permeabilization with the MSP1 monoclonal antibody 89.1 (1:100), and subsequently with anti-mouse Alexa488 (1:3000). The population of uRBCs, schizonts, rings, and merozoites attached to the RBC surface were quantified using FlowJo.

### Live microscopy imaging of egress and DPAP3 secretion

Time lapse video microscopy of egress was performed as previously described[9]. Briefly, tightly synchronized schizonts were percoll-enriched and arrested with 1 μM C2 for 4 h. After C2 wash out, DIC and mCherry images were collected every 5 and 25 s, respectively, for 30 min using a Nikon Eclipse Ni-E wide field microscope fitted with a Hamamatsu C11440 digital camera and a Nikon N Plan Apo λ 100x/1.45NA oil immersion objective. For each experiment, videos of the RAP- and DMSO-treated parasites were taken alternately to ensure that possible differences in the rate of egress were not a result of variation in the maturity of the parasite populations. The images were then annotated using Axiovision 3.1 software and exported as AVI movie or TIFF files. Individual egress events were annotated by detailed visual analysis of the movies, and the delay to the time of egress was recorded for each schizont for subsequent statistical analysis. Mean fluorescence intensity values of individual mCherry-expressing schizonts right before and after PVM breakdown were determined from exported raw image files (TIFF format) as described previously[9] and using the elliptical selection tool and ‘Histogram’ options of ImageJ/Fiji V1.0. DPAP3KO parasites were analyzed to determine the residual background fluorescence derived from the hemozoin.

### Western blots

Saponin pellets of parasites from different erythrocytic stages as well as samples from culture supernatant harvested during egress and invasion were syringe filtered (Minisart, 0,2 μm, Sartorius), boiled in SDS-PAGE loading buffer under reducing conditions, run on a SDS-PAGE gel, and transferred to Hybond-C extra nitrocellulose membranes (GE Healthcare). The membranes were blocked with 5% (w/v) nonfat milk in PBS, probed with monoclonal or polyclonal antibodies (see S3 Table for antibodies used in this study), and followed by application of horseradish peroxidase-conjugated secondary antibodies (Pierce). The signal was detected using SuperSignal West Pico chemiluminescent substrate (Thermo Scientific) and a ChemiDoc MP imager (BioRad).

### Diagnostic PCR

PCR was performed using GoTag (Promega), Advantage 2 (Clontech), or Q5 High-Fidelity (NEB) polymerases. Diagnostic PCR to detect integration of targeting constructs was performed using extracted genomic DNA as template. Primer pairs specific for detection of integration, namely P21 and P22 for integration of pHH1-chDPAP3-mCh and pHH1-chDPAP3-HA, and II-inte_F and II-wt_R for integration of pPM2GT-DPAP3Ct-GFP, were designed such that the forward primer hybridized in a genomic region upstream of the plasmid homology region, and the second in a region unique to the introduced plasmid. Primer pairs P21 and P23 were designed to detect presence of the unmodified *dpap3* locus. To assess whether *dpap3-mCh* had been excised after RAP treatment, diagnostic PCR was performed using extracted genomic DNA as template. Primers P24 and M13 were used to detect non-excision *dpap3* at the genomic locus and hybridize upstream and downstream of the second *loxP* site, respectively. Primers P25 and SP6 were used to detect presence of excision and hybridize upstream and downstream of the first and second *loxP* sites, respectively.

## Supporting information

S1 Text Supplementary methods and discussion. (DOCX)

S1 Table Data from all plaque assay experiments. (DOCX)

S2 Table List of primers used in this study. (DOCX)

S3 Table List of all primary antibodies used for WB and IFA studies. (DOCX)

S1 Fig Strategy to GFP-tag the endogenous *dpap3* locus and time course of DPAP3-HA and DPAP3-mCh expression. (Related to Fig 1) (**A**) Schematic representation of the *dpap3* recombinant genetic locus. The endogenous locus (original locus) harbours two exons (EX1 and EX2 grey boxes), an intron (pink box; IN) and the 3’ regulatory sequence (black circle). The plasmid used to GFP-tag DPAP3 is made of a C-terminal homology region containing part of EX2 (1 kb), followed by a *gfp* sequence (0.7 kb, green box; GFP), a *hsp86* 3’ regulatory sequence (0.9kb, white circle), and the *hdhfr* resistance cassette (2 kb, black box). After single homologous recombination, the mutated locus harbours the *dpap3-GFP* sequence, and the truncated endogenous EX2 locus is displaced. (**B**) Integration efficiency at the *dpap3* locus assessed by PCR on genomic DNA using the II-inte_F forward primer (black arrows) and the II-wt_R (orange arrows) or II-inte_R (blue arrow) reverse primers for the endogenous or modified locus, respectively. Primer binding sites are indicated in **A**. (**C**) IFA of parasites collected 9–48 h.p.i. from DPAP3-HA and DPAP3-mCh were fixed and stained with mouse anti-GAP45 (red) and rat anti-HA (left panel) or rabbit anti-mCherry (right panel) antibodies (both green). DNA was stained with DAPI (blue). IFA was analysed by confocal microscopy. Scale bar: 10 μm (9–43 h.p.i.) and 5 μm (48 h.p.i.). (**D**) WB analysis of DPAP3-HA parasite lysates harvested at different h.p.i. DPAP3-HA was detected using an anti-HA antibody. An MSP1 antibody was used to confirm that the low level of DPAP3 observed at ring and trophozoite stages is not due to schizont contamination in our samples. (**E**) Quantification of DPAP3-mCh parasites showing negative (white bar), cytoplasmic (grey bar) or apical (black bar) DPAP3 staining during schizogony (36 to 48h.p.i.). Schizont maturity was assigned based on the number of nuclei per iRBC. Quantification of DPAP3-HA parasites is shown in Fig 1G.
(TIF)

S2 Fig IFA of DPAP3 expression and localization. (Related to Fig 1F) (**A**) DPAP3-HA parasites collected 39–48 h.p.i. were fixed and stained with mouse anti-SUB1, rabbit anti-AMA1, mouse anti-RopH2, mouse anti-RON4 (all red), and rat anti-HA (green). For the 39 h.p.i. time point, we also show images of iRBCs that were lagging behind in development, i.e. containing only one nucleus. These images were collected from the same slides as the one of schizonts shown underneath and indicate that the diffuse staining observed in early schizonts is not due to background fluorescent signal. (**B**) IFA of DPAP3-mCh C2-arrested (upper panel) or rupturing (DMSO, lower panel) schizonts that were fixed 48 h.p.i. and stained with mouse anti-Exp2 (red) and rat anti-mCherry (green). (**C**) IFA of DPAP3-HA schizonts fixed 48 h.p.i. and stained with rat anti-EBA175 (red) and mouse anti-HA (green). (**A-C**) DNA was stained with DAPI (blue); scale bar: 5 μm. All IFAs were analysed by confocal microscopy.
(TIF)

S3 Fig DPAP3 localization studies by SIM and IEM. (Related to Figs 1H and 2D) (**A**) IFA of DPAP3-HA C2-arrested schizonts stained with rat anti-HA (green) and mouse anti-SUB1, mouse anti-RON4 and rat anti-EBA175 (all red). (**B**) Same IFA as in A but for DPAP3-GFP parasites. For this line staining with mouse anti-RopH2 (red) is also shown. DPAP3-GFP as well as DPAP3-HA forms small dot like structures at the apical pole that do not colocalize with any of the used apical marker proteins. (**C**) IFA of a late schizont from a SUB1-HA line (3D7SUB1-HA3)[10] was used as a control for colocalization at the apical pole using SIM. Parasite was fixed and stained with mouse anti-SUB1(red) and rat anti-HA (green). (**A-C**) DNA was stained with DAPI (blue); scale bar: 5 μm. All IFAs were analysed by SIM. Overlay of the staining is shown. (**D**) IEM sections of 3D7 schizonts stained with rabbit anti-GFP and colloidal gold-conjugated anti-rabbit antibodies. No significant unspecific labelling was observed on the 3D7 control line. (**E**) IEM sections corresponding to the uncropped images shown in Fig 2D. Dotted rectangles delineate the cropped images shown in Fig 2D. (**D-E**) Scale bar: 200 nm.
(TIF)

S4 Fig Biochemical fractionation of parasite cultures showing DPAP3 secretion at the time of egress. (Related to Fig 2) (**A**) WB analysis showing that the FY01-labelled band at 130kDa correspond to DPAP3-HA. C2-arrested schizonts were either left on C2, treated with E64 after C2 wash out, or allowed to egress for 1h in the presence of FY01 (Same samples as in Fig 2A). Parasite pellets from free merozoites and schizonts (insoluble fraction obtained after saponin lysis), proteins precipitated from the culture supernatant, and PV and RBC cytosol components (soluble saponin fraction), were run on a SDS-PAGE. DPAP3 labelling by FY01 can be observed as a fluorescent band at 130kDa, which correspond to the band identified by WB using an anti-HA antibody. Note that FY01 is also able to label other papain-fold cysteine proteases such as the falcipains (FP1-3) or DPAP1 (indicated by arrowheads). (**B**) Rupturing (DMSO) and C2- or E64-arrested 3D7 schizonts were labelled under intact conditions with FY01 in the presence or absence of the DPAP3 inhibitor SAK1. Proteins secreted in the culture supernatant, free merozoites, and the soluble and insoluble fractions of saponin lysed schizonts, were run on an SDS-PAGE gel. Fluorescent bands at 130 and 100 kDa that disappear in the presence of SAK1 correspond to the p120 and p95 forms of DPAP3-HA. The small proportion of p95 DPAP3 is likely produced after parasite lysis. (**C**) C2-arrested schizonts (DPAP3-HA line) were either left on C2 or allowed to egress for 30 min after C2 wash out. Unruptured schizonts, free merzoites, and proteins secreted in the culture supernatant were collected, and the presence of DPAP3-HA in each fraction visualized by WB using an anti-HA antibody.
(TIF)

S5 Fig Removal of the prodomain of DPAP3 is not required for activation. (Related for Fig 3) (**A**) Schematic representation of DPAPs processing. The signal peptide (SP), exclusion domain (ED), prodomain (PD), and the N- and C-terminal portions of the catalytic domain (Nt-CD and Ct-CD) are shown in different colours. Cleavage sites for removal of the SP and PD, and for processing of the CD are indicated by chevrons. A representation of fully processed monomeric DPAP1 and tetrameric CatC are shown. The predominant p120 DPAP3 active form is produced after removal of the SP. We propose that the p95 DPAP3 active form is produced after cleavage of the ED. The regions recognized by anti-Nt-DPAP3 and anti-Ct-DPAP3 antibodies, and the position of the catalytic Cys covalently modified by FY01 are shown above the full-length protein scheme. (**B**) Western blot analysis of DPAP3 processing in schizonts. Supernatant of insect cell cultures expressing rDPAP3, lysates of DPAP3-HA schizonts, and 3D7 mature schizonts directly boiled into loading buffer without previous lysis were analysed by WB using the α-DPAP3Ct, and with α-HA after stripping the same blot. Lysis of schizonts before addition of loading buffer results in processing of DPAP3 from the p120 form to the p95 and p40 forms (N- and C-terminal portion of the CD, respectively). However, if schizonts are directly boiled in loading buffer, only the physiologically relevant p120 form is observed (see also Fig 1). Note that the p120 and p95 forms obtained from rDPAP3 are very similar to the ones that are observed in parasite lysates. Asterisks indicate non-specific labelling by α-DPAP3Ct. (**C**) Representative data showing DPAP3 processing is an artefact of parasite lysis. Merozoite lysates were incubated for several hours under neutral (PBS, pH 7.2) or acidic (acetate buffer, pH 5.5) conditions before adding 1 μM of FY01 for 1 h. Under acidic conditions, we observed a time dependent processing of the p120 form to the p95 and p42 forms. Coomassie staining of the gel is shown as a loading control. This experiment was repeated four time: 2 biological replicates with two technical replicates each. (**D**) Quantification and normalization of the fluorescent signal for the different labelled forms of DPAP3. The fluorescent intensity of each band was quantified using ImageJ and normalized to the sum of fluorescence intensities measured at time zero. Error bars represent standard error of the mean (N = 4). For all replicates, only the p120 band was observed at neutral pH. At acidic pH we see a time dependent decrease and increase of the p120 and p42 forms, respectively, and an accumulation of the p95 form at intermediate time points. (**E**) Michaelis Menten plots showing VR-ACC turnover rate for two consecutive Ni-NTA elution fractions (E2 and E3). The gel image showing FY01 labelling indicates that E2 contains predominantly the p120 form, and E3 a mixture of the p95 and p120 forms. VR-ACC turnover was normalized to the sum of p120 and p95 labelling shown in the gel image. The identical Michaelis Menten plots indicate that the p120 and p95 forms are equally active, thus showing that removal of the signal peptide is sufficient to activate DPAP3.
(TIF)

S6 Fig Integration of *dpap3* cKO constructs and localization of DPAP3-HA in complementation lines. (Related to Figs 4 and 5) (**A**) Schematic representation of the *dpap3* recombinant genetic locus. The endogenous locus is represented as described in S1A Fig. The plasmid used to integrate two *loxP* sites into the ORF is made of a homology region containing the DPAP3 intron and part of its two exons (0.2 kb of EX1 and 0.8 kb of EX2) and the rest of EX2 as a recodonized sequence (1.8 kb, sc EX2; green box) containing a *loxP* site (yellow arrow) at its 5’ end, either as a *loxPint* (0.1 kb, box with white-pink stripes and yellow arrow, F8cKO and F3ckO) or integrated in the ORF for A1cKO (not shown here but represented in Fig 4A). This is followed by an *mCherry* sequence (0.7 kb, red box; mCh), a *P*. *berghei* 3’ regulatory sequence (0.8 kb, white circle), the 2^nd^ *loxP* site (yellow arrow, clone E7ctr only harbours this *loxP* site), and the *hdhfr* resistance cassette (2 kb, black box). After single homologous recombination, the mutated locus harbours the chimeric *dpap3-loxP-mCherry* sequence, and the truncated endogenous WT *dpap3* locus is displaced. The constructs used for episomal complementation of DPAP3 contain a promoter region (dark grey box; prom), which was either the 5’ regulatory sequence of *ama1* (1.5 kb) or *dpap3* (0.9 kb). This was followed by a recodonized version of the *dpap3* gene (green box, sc*dpap3*) fused to a HA_3_ tag (purple box), a *P*. *berghei* 3’ regulatory sequence, and a pyromycin resistance cassette (2.2 kb, black box; *pyro*), which is driven by the *cam* 5’ regulatory sequence. (**B**) Integration efficiency at the *dpap3* locus in transgenic parasites was assessed by PCR on genomic DNA using primers P21 and P23 for the endogenous locus, and P21 and P22 for mutated locus. P21, P22, and P23 binding sites are indicated in (**A**) by black, blue and orange arrows, respectively. PCR was performed on genomic DNA purified from the E7ctr, A1cKO, F3cKO, and F8cKO parasite lines. 1G5 parasites were used as a negative control for integration. The predicted size of the DNA fragment amplified from the *dpap3*-wild type locus is 1.6 kb, and those for integration of A1cKO, 1.91 kb; E7ctr, 1.8 kb; and F8cKO or F3cKO, 1.85 kb. Asterisks indicate non-specific PCR products. (**C**) IFA of schizonts collected 48 h.p.i. from the F8cKO+WT_dpap3/ama1_ or F8cKO+MUT_dpap3/ama1_ parasite lines. Parasites were fixed and stained with rat anti-HA (red) and mouse anti-mCherry (green). DNA was stained with DAPI (blue). IFAs were analysed by confocal microscopy. Scale bar: 5 μm.
(TIF)

S7 Fig Effect of RAP treatment on parasite development. (Related to Fig 6) (**A**) F8cKO and A1cKO parasites were either treated with DMSO or RAP, and schizonts purified on cycle 5 (c5) after treatment, fixed, and stained with rat anti-mCherry (green), mouse anti-SUB1 (red), and DAPI (blue). The percentage of mature schizonts that were positive for mCherry staining was calculated. SUB1 staining was used as a marker of schizont maturity. As expected all mature schizonts in DMSO treated parasites were mCherry positive, but only 40% were mCherry negative five cycles after RAP treatment, indicating that non-excised parasites grow faster than excised ones. Each circle indicates a biological replicate with filled and empty circles corresponding to the F8cKO and A1cKO lines, respectively. The significance p-value calculated with a Student’s t test is show. (**B**) Quantification of late schizont development in cKO and complementation lines. After DMSO or RAP treatment of the E7ctr, F8cKO (full circles), F3cKO (grey circles), A1cKO (empty circles), or F8cKO+WT/MUT_ama1/dpap3_ (full circles) lines at ring stage, schizonts were collected at 48 h.p.i., fixed, and stained with anti-mCherry and anti-SUB1 antibody as a marker of schizont maturity. The amount of mature schizonts was quantified and plotted as the ratio between RAP and DMSO treatment. Each circle corresponds to a different biological replicate. The E7ctr lines was used as a negative control of excision. No significant differences between RAP and DMSO treatment was observed for any of the parasite lines using a Student’s t test. (**A-B**) A minimum of 100 parasites was analysed per biological replicate. (**C**) AMA1 and RopH2 are expressed and localize to the apical pole in DPAP3-KO parasites. F8cKO and E7ctr parasites were treated with RAP or DMSO, fixed at 48 h.p.i., and stained with rat anti-mCherry (green) and rabbit anti-AMA1 or mouse anti-RopH2 (both red). No differences in the localization or appearance of both apical markers could be found between RAP- and DMSO-treated parasites. (**D**) Rhoptry and exoneme proteins do not misslocalize with micronemal proteins in DPAP3KO parasites. A1cKO parasites were either RAP or DMSO treated, fixed at 48 h.p.i., and stained with rat anti-EBA175 (green) and mouse anti-RON4, mouse anti-RopH2, or mouse anti-SUB1 (all red). No differences in the localization or appearance of apical markers could be found between DMSO and RAP treated parasites. (**B-D**) DNA was stained with DAPI (blue). IFAs were analysed by confocal microscopy. Scale bar: 5μm.
(TIF)

S8 Fig SAK1 and WSAK inhibit schizont development. (Related to Fig 7) (**A**) 3D7 parasites were treated for 6 h with 1 μM C2, 50 μM SAK1, or 50–100 μM WSAK starting at 42 h.p.i. (T0). Schizonts were fixed before and after treatment and stained with rabbit anti-AMA1 (red) and human anti-MSP1 (green). DNA was stained with DAPI (blue). IFAs were analysed by confocal microscopy. Scale bar: 10 μm. (**B**) The amounts of segmented (black) or non-segmented (grey) schizonts was quantified based on the MSP1 staining pattern. Representative images for each category are shown. Results are the means ± standard deviation from three independent biological replicates (Note that treatment at 100 μM WSAK was only performed once). Differences between T0 and the differently treated parasite populations were compared using a Student’s t test, with all significant results indicated. (**C**) Quantification of the number of nuclei per iRBC. The number of nuclei per iRBC was quantified from the IFA and is shown as a dot plot. Average number of nuclei/iRBC is indicated below the graph. Results are the means ± standard deviation from one representative experiment. Differences between T0 and the differently treated parasite populations were compared using a Student’s t test, with all significant results indicated.
(TIF)

S9 Fig FACS analysis of extracellular merozoites. (Related to Fig 8C) (**A**) **Left:** C2-arrested A1cKO schizonts pretreated with DMSO or RAP were incubated with fresh RBCs after C2 removal. Samples were collected at the indicated time points, fixed, and stained with Hoechst and WGA-Alexa647. The populations of schizonts, rings, and free merozoites were quantified by FACS and are shown in the bar graph. **Right:** Same type of staining and FACS analysis was performed on the samples collected at the indicated time points (h.p.i.) after DMSO or RAP treatment. The fixed samples used for this experiment were the same as the ones shown in Fig 6E. (**B**) Effect of mixing WT and DPAP3KO parasites on RBC invasion. To determine whether secretion of DPAP3 in the culture supernatant could rescue the invasion defect of DPAP3KO parasites, we mixed equal amounts of E7ctrl and DPAP3KO schizonts (obtained after RAP treatment of A1cKO) with fresh RBCs and compared the invasion efficiency of this mixed culture with that of E7ctrl or A1cKO after DMSO or RAP treatment. All cultures were setup at 2% parasitemia and incubated overnight under shaking conditions. The percentage of rings obtained for each culture is shown. Each circle represents a different biological replicate. The dotted line marks the expected invasion rate for the 1:1 mixture of E7ctr and A1cKO after RAP treatment assuming independent effects between the two lines, i.e. no rescue effect. This value (10.1%) was calculated as the average between the E7ctr and A1cKO+RAP invasion rates. Differences between the different parasite populations were compared using a Student’s t test, with all significant results indicated.
(TIF)

S10 Fig Cloning strategies for cKO and tagging of DPAP3. (**A**) Constructs pHH1-chDPAP3-mCh, pHH1-chDPAP3-HA were designed to integrate by single-crossover homologous recombination into the 3D7 *dpap3* locus, reconstituting the coding sequence of the endogenous gene with a gene that expresses a chimeric (ch) DPAP3 protein fused to either mCherry or a triple HA tag. To reliably introduce a mutation at the catalytic cysteine residue, the DNA sequence in the entire C-terminal region (1268 bp) was recodonized in the resultant plasmid (*syn_dpap3*). Recodonized regions (RR) are shown as grey boxes. Upstream of the recodonized sequence was a 1210 bp endogenous 3D7 sequence (homology region, HR, that excludes the 58 bp sequence coding for the signal peptide) to drive single cross-over homologous recombination at the *dpap3* locus. White block arrows in the scheme indicate PCR amplification and black block arrows ligation after digest with the indicated restriction enzymes. The 1210 bp targeting fragment (HR, white box) was created by amplifying *P*. *falciparum* 3D7 genomic DNA using primers P5 (forward, has BamHI site) and P6 (reverse, has PstI site) and ligated into pFB-rDPAP3 or pFB-rDPAP3mut after restriction digest with BamHI and PstI resulting in plasmids pFB-chDPAP3 and pFB-chDPAP3mut, respectively. Plasmid pHH1-SERA5-loxP-DS_PbDT3’, which harbors the coding sequence for a C-terminal HA_3_ tag (light blue box), a *loxP* site (orange arrow) downstream of the Pb3’ UTR, and a *hdhfr* resistance cassette (black box)[33], was digested with BglII and XhoI (deleting the C-terminal part of SERA5 in this plasmid), blunted, and re-ligated in order to create a BglII restriction site at the 3’ end (resulting in plasmid pHH1-SERA5ΔCt-HA). The sequence for mCherry (red box) was amplified from plasmid pREST-B mCherry[55] with primer P7 (forward, has SalI and XhoI sites) and P8 (reverse, has SpeI and NotI sites) and ligated into plasmid pHH1-SERA5ΔCt-HA after digestion with XhoI and NotI, resulting in exchange of HA_3_ with *mCherry* and plasmid pHH1-SERA5ΔCt-mCh. The chimera DPAP3 and DPAP3mut fragments were subsequently amplified from pFB-chDPAP3 and pFB-chDPAP3mut plasmids using primers P9 (forward, has KpnI and HpaI sites) and P10 (reverse, has XmaI and BglII sites) and ligated into plasmids pHH1-SERA5ΔCt-HA or pHH1-SERA5ΔCt-mCh after restriction digest with HpaI and BglII, resulting in plasmids pHH1-chDPAP3-HA, pHH1-chDPAP3mut-HA, pHH1-chDPAP3-mCh and pHH1-chDPAP3mut-mCh. (**B**) Constructs pPM2GT-DPAP3Ct-GFP was designed to integrate by single-crossover homologous recombination into the 3D7 *dpap3* locus, reconstituting the coding sequence of the endogenous gene with a gene that expresses a wildtype DPAP3 protein fused to GFP (green box). A 1065bp endogenous 3D7 sequence piece, excluding the STOP codon (white box, HR) was used to drive single cross-over homologous recombination at the *dpap3* locus. The HR fragment was amplified from *P*. *falciparum* 3D7 genomic DNA using primers II-DPAP3Ct_F (forward, has XhoI site) and II-DPAP3Ct_R (reverse, has AvrII site) and ligated into pPM2GT [58] after restriction digest with XhoI and AvrII, resulting in plasmid pPM2GT-DPAP3Ct-GFP. (**A-B**) The constructs were transfected into 3D7 parasites. (**C**) To obtain conditional truncation of the *dpap3* gene, we used two different approaches. One introduced a *loxP* site (orange arrow) into the ORF of the *dpap3* gene within a sequence coding for an asparagine-rich region upstream of the catalytic domain, resulting in a partial replacement of the endogenous sequence (left side). The second approach made use of a silent *loxP* site within a heterologous *P*. *falciparum* intron (called *loxPint*, orange arrow within yellow box), which was inserted in the sequence **AGAT** (see below) 50 bp before the sequence coding for the above-mentioned Asn-stretch (right side). The *loxPint* system has been described in detail elsewhere[36]. Using these two approaches, we created two slightly different truncations of DPAP3 (F8cKO and F3cKO, *loxPint*; and A1cKO, *loxP*; see Fig 4A and S6A Fig). For the first approach (left side), two PCR fragments coding for the N- and C-terminal part of the protein were amplified using primers P11 and P12 (including KpnI and AgeI+loxP, respectively; N-term), and P13 and P14 (including AgeI and SmaI, respectively; C-term), and introduced into construct pHH1-chDPAP3-mCh using restriction sites KpnI, AgeI and SmaI to generate plasmid construct pHH1-chDPAP3_loxP-mCh. For the second approach (right side), a ~1600 bp sequence corresponding to the *loxPint* fragment and flanked by targeting sequence at the 5ˊ and 3ˊ ends **(**…AAAGAAGA**AG**-loxPint-**AT**CATCAACA…) was synthesized (GeneWiz) and introduced into construct pHH1-chDPAP3-mCh using restriction sites KpnI and ClaI to generate plasmid construct pHH1-chDPAP3_loxPint-mCh. All constructs were transfected into 1G5-DiCre parasites[33]. (**D**) For episomal complementation of the cKO lines, plasmids pHH1-chDPAP3-HA and pHH1-chDPAP3mut-HA (**A**) were modified in order to express full length WT or MUT *dpap3* under the control of either *dpap3* or *ama1* promoters. Firstly, the puromycin N-acetyltransferase drug selection marker (pac, purple box) was amplified from mPAC-TK (a kind gift of Alex Maier) using primers CVO140 and CVO141, and ligated into pHH1-chDPAP3-HA and pHH1-chDPAP3mut-HA after digestion with BamHI and HindIII, thereby replacing the *hdhfr* gene. To avoid integration at the mutated *dpap3* locus in our cKO lines, the DNA sequence of the entire gene was recodonized in the resultant plasmid. To do that, the sequence for rDPAP3-Nt was amplified by PCR from plasmid puc57-rDPAP3-Nt (see material and methods for details about this plasmid) using primers P15 and P16, and ligated into the pHH1-chDPAP3-HA and pHH1-chDPAP3mut-HA vectors after restriction digestion with KpnI and ClaI, resulting in plasmids pHH1-rDPAP3-HA and pHH1-rDPAP3mut-HA. The *ama1* and *dpap3* promoter regions were amplified from *P*. *falciparum* 3D7 genomic DNA using primers P17 and P18 (*ama1*) and P19 and P20 (*dpap3*), and ligated into pHH1-rDPAP3-HA and pHH1-rDPAP3mut-HA after digestion with HpaI and KpnI, resulting in transfection plasmids pHH1-*ama1*-rDPAP3-HA (parasite line cKO+WT_ama1_), pHH1-*ama1*-rDPAP3mut-HA (parasite line cKO+MUT_ama1_), pHH1-*dpap3*-rDPAP3-HA (parasite line cKO+WT_dpap3_) and pHH1-*dpap3*-rDPAP3mut-HA (parasite line cKO+MUT_dpap3_). All constructs were transfected into A1cKO or F8cKO parasites. All final construct sequences were verified by nucleotide sequencing on both strands.
(TIF)

S11 Fig DPAP inhibitors purity. (Related to Fig 7A) LCMS UV trace showing the purity of SAK1, *L*-WSAK, and *D*-WSAK.
(TIF)

S1 Video DPAP3-mCh is secreted upon PVM breakdown. (Related to Fig 2B and 2C) Synchronized E7ctr parasites at schizont stage were Percoll-enriched, returned to culture, and allowed to mature for 4–5 h in the presence of C2, then washed in warm medium without C2, and immediately observed by time-lapse DIC and fluorescent microscopy, taking images at 5 and 25 s intervals in the DIC (left video) and mCherry (right video) channels, respectively. Imaging started 6 min after C2 wash out. In the DIC channel breakdown of the PVM and subsequently of the RBCM can be observed. The intensity of the mCherry signal drops at the same time as PVM breakdown but remains equally strong in parasites that do not undergo PVM breakdown. Scale bar 10 μm. DPAP3 secretion can also be clearly observed in the egress S4 Video for DMSO-treated F8cKO parasites.
(AVI)

S2 Video DPAP3-mCh is secreted upon PVM breakdown, second representative replicate. (Related to Fig 2B and 2C) Parasite egress was monitored by live microscopy as described for S1 Video. Scale bar 10 μm.
(AVI)

S3 Video DPAP3-mCh is secreted upon PVM breakdown, third representative replicate. (Related to Fig 2B and 2C) Parasite egress was monitored by live microscopy as described for S1 Video. Scale bar 10 μm.
(AVI)

S4 Video DPAP3 knock-out parasites egress from erythrocytes. (Related to Fig 7C) F8cKO parasites were treated with DMSO (upper panel) or RAP (lower panel) at ring stage and cultured for 44 h to allow schizont development. The schizonts were Percoll-enriched, returned to culture and allowed to mature for 4–5 h in the presence of C2, then washed in warm medium without C2, and immediately observed by time-lapse DIC and fluorescent microscopy, taking images at 5 and 25 s intervals in the DIC (left videos) and mCherry (right videos) channels, respectively. Imaging started 6 min following C2 removal. As can be seen one parasite on the RAP treated population has not gone through excision and expresses DPAP3-mCherry. Time after start of microscopy is indicated on the top right corner of the video. No differences were noticed in the number of parasites that egressed, nor on the timing of egress between DMSO and RAP treated parasites. Identical results were observed for the A1cKO lines (videos not shown).
(AVI)
