# Supplementary material for: Plasmodium falciparum dipeptidyl aminopeptidase 3 activity is important for efficient erythrocyte invasion by the malaria parasite
Source: PLoS Pathog. 2018 May 16;14(5):e1007031. doi: 10.1371/journal.ppat.1007031 (PMC5973627; doi:10.1371/journal.ppat.1007031)
Supplement: S1 Text — (DOCX) [file ppat.1007031.s001.docx]

***Plasmodium falciparum* dipeptidyl aminopeptidase 3**

**activity is important for efficient erythrocyte**

**invasion by the malaria parasite.**

# Supplementary Materials

# Supplementary Results and Discussion

**Removal of the prodomain is not required for DPAP3 activation.** DPAPs are composed of a signal peptide, an exclusion domain, a prodomain, and a C-terminal catalytic domain. Processing of DPAPs has mainly been studied for the mammalian homologue cathepsin C (CatC) and starts with removal of the signal peptide in the ER upstream of the exclusion domain N-terminal aspartate. This residue is critical for activity as it directly interacts with the free N-terminal amine of DPAP substrates [1]. Further processing consists of removal of the inhibitory prodomain and cleavage of the catalytic domain downstream of the catalytic Cys, which for CatC has been shown to be mediated by lysosomal cysteine cathepsins (S5A Fig). Thus, each DPAP proteolytic unit is generally composed of three oligopeptides (exclusion domain and the N- and C-terminal portions of the catalytic domain), and CatC is composed of four proteolytic units [2]. Although *Plasmodium* DPAP1 seems to be processed in a similar way [3], its final form is composed of a single proteolytic unit [3], thus showing that tetramerization is not required for DPAP activity (Fig S5A).

Three different isoforms of DPAP3 consistent with the canonical processing of DPAPs were previously shown to be labelled by FY01 in merozoite lysates under acidic conditions [4] (p120, p95, and p42 forms). However, when intact schizonts or merozoites are directly boiled in SDS-PAGE loading buffer, we predominantly detect the p120 form either by WB or FY01 labelling (Figs 1-2 and S5B Fig), suggesting that DPAP3 processing might be an artefact of parasite lysis. To confirm this, we show that incubation of merozoite lysates under acidic conditions results in a time-dependent processing of DPAP3, which is not observed at neutral pH (Fig S5C-D).

Although FY01-labelling of the p120 ‘zymogen’ form indicates that its active site is accessible and contains a nucleophilic cysteine, it does not demonstrate that it can turnover substrates. To address this point, we expressed full-length rDPAP3 (rDPAP3) in insect cells. Our purification from the culture supernatant yielded predominantly the p120 and p95 forms (Fig 3A), which are able to cleave the VR-ACC fluorogenic DPAP substrate [5] (Fig 3B). The detection of the p95 in lysates from our different DPAP3 tagged lines indicates that the p120 form is likely cleaved between the exclusion domain and the prodomain to yield the p95 form (Fig 3A). Given their molecular weights, both p120 and p95 forms must contain the prodomain, indicating that its removal is not required for DPAP3 activation.

In one of our purifications we were able to separate the p120 form from a fraction containing a mixture of the p95 and p120 forms (Fig S5E). Although rDPAP3 was more abundant in the latter fraction, when VR-ACC turnover was normalized to the total amount of FY01 labelling (i.e. p120+p95 labelling), both fractions showed identical *V*_max_ and *K*_m_ values, suggesting that these two forms are equally active, and therefore, that removal of the signal peptide is sufficient to activate DPAP3. The facts that the p120 form of rDPAP3 is active, and that this is the predominant form found in live parasites, strongly suggest that this “zymogen” form is the one performing a biological function in parasites.

# Supplementary Methods

**Synthesis and characterization of DPAP inhibitors:**

(S)-3-benzenesulfonyl-1-phenethylallylamine trifluoroacetate was synthesized as previously described [6]. LCMS traces of each purified inhibitors are shown in S11 Fig.

**SAK1:**((2S)-2-amino-3-(4-hydroxy-3-nitrophenyl)-N-((S,E)-5-phenyl-1-(phenylsulfonyl)pent-1-en-3-yl)propanamide.

To a solution of (S)-N-Boc-Piperidine-2-carboxylic acid (211 mg, 0.92 mmol) in DMF (3 mL) was added EDC/HCl (177 mg, 0.92 mmol), HOBt (125 mg,0.92 mmol) and DIEA (0.6 mL, 3.1 mmol). After 10 min, (S)-3-benzenesulfonyl-1-phenethylallylamine trifluoroacetate (TFA•HphVSPh) (320 mg, 0.77 mmol) in DMF (3 mL) was added drop-wise. The reaction was stirred at room temperature (RT) for 12 h. The resulting solution was evaporated in vacuo to give a light yellow oil, which was diluted with aqueous NH_4_Cl (10 wt%) and extracted with EtOAc (3 x 50 mL). The combined organic extracts were dried over Na_2_SO_4_, filtered and concentrated in vacuo. Purification by flash column chromatography (silica gel; using 40% EtOAc in hexanes) provided the protected product as a white foam.

To a solution of this intermediate in CH_2_Cl_2_ (5 mL) at 0 °C was added trifluoroacetic acid (5 mL). The solution was stirred at RT overnight, then evaporated to dryness in vacuo and finally freeze-dried yielding a white solid that was identified as the trifluoroacetic salt of the desired product (216 mg, 0.41 mmol, 52%).

**^1^H NMR (250 MHz, *MeOD-d_4_ δ*)**: 1.80 (m, 2H), 2.57-2.65 (m, 2H), 3.12 (dq, *J =* 13.9, 7.3 Hz, 2H), 4.09 (t, *J =* 7.3 Hz, 1H), 4.58 (dd, *J =* 12.8, 6.3 Hz, 1H), 6.26 (dd, *J =* 15.1, 1.2 Hz, 1H), 6.76 (dd, *J =* 15.1, 5.8 Hz, 1H), 7.11-7.26 (m, 6H), 7.46 (dd, *J =* 8.6, 2.2 Hz, 1H), 7.57-7.72 (m, 3H), 7.84-7.87 (m, 2H), 8.01 (d, *J =* 1.9 Hz, 1H).

**^13^C NMR (*MeOD-d_4_ δ*)**: 32.8 (CH_2_), 36.2 (CH_2_), 37.2 (CH_2_), 50.9 (CH), 55.2 (CH), 121.7 (CH), 126.9 (CH), 127.2 (CH), 127.3 (C), 128.7 (CH), 129.3 (CH), 129.5 (CH), 130.6 (CH), 132.4 (CH), 134.8 (CH), 135.7 (C), 138.8 (CH), 141.5 (C), 141.9 (C), 146.3 (CH), 155.0 (C), 168.8 (C).

**ESI-MS**: [M+H]^+^ calcd. for C_26_H_28_N_3_O_6_S = 510.1693 found 510.1701. (M.W. 606.5901)

***L-*WSAK:** (2S)-2-amino-3-(1H-indol-3-yl)-N-((S,E)-5-phenyl-1-(phenylsulfonyl)pent-1-en-3-yl)propanamide.

To a solution of (S)-N-Boc-Piperidine-2-carboxylic acid (211 mg, 0.92 mmol) in DMF (3 mL) was added EDC/HCl (177 mg, 0.92 mmol), HOBt (125 mg, 0.92 mmol) and DIEA (0.6 mL, 3.1 mmol). After 10 min, (S)-3-benzenesulfonyl-1-phenethylallylamine trifluoroacetate (TFA•HphVSPh) (320 mg, 0.77 mmol) in DMF (3 mL) was added drop-wise. The reaction was stirred at RT for 12 h. The resulting solution was evaporated in vacuo to give a light-yellow oil, which was diluted with aqueous NH_4_Cl (10 wt%) and extracted with EtOAc (3 x 50 mL). The combined organic extracts were dried over Na_2_SO_4_, filtered and concentrated in vacuo. Purification by flash column chromatography (silica gel; using 40% EtOAc in hexanes) provided the protected product as a white foam.

To a solution of this intermediate in CH_2_Cl_2_ (5 mL) at 0 °C was added trifluoroacetic acid (5 mL). The solution was stirred at RT overnight, then evaporated to dryness in vacuo and finally and freeze-dried yielding a white solid that was identified as the trifluoroacetic salt of the desired product (216 mg, 0.41 mmol, 52%).

**^1^H NMR (250 MHz, MeOD-*d_4_***): 1.78-1.93(m, 2H), 2.54-2.62 (m, 2H), 3.15-3.24(m, 2H), 4.11 (t, *J* = 7.4 Hz, 1H), 4.55 (dd, *J* = 12.5, 6.1 Hz, 1H), 6.13 (dd, *J* = 15.0, 0.9 Hz, 1H), 6.77 (dd, *J* = 15.2, 5.5 Hz, 1H), 7.03-7.26 (m, 8H), 7.41 (d, *J* = 7.9 Hz, 1H), 7.54-7.67 (m, 4H), 7.80-7.83 (m, 2H).

**^13^C NMR (MeOD-*d_4_*):** 29.1 (CH_2_), 32.9 (CH_2_), 36.3 (CH_2_), 51.0 (CH), 54.9 (CH), 108.0 (C), 113.0 (CH), 119.1 (CH), 120.4 (CH), 123.1 (CH), 125.5 (CH), 127.2 (CH), 128.1 (C), 128.8 (CH), 129.4 (CH), 129.6 (CH), 130.6 (CH), 132.4 (CH), 134.8 (CH), 138.2 (C), 141.6 (C),142.1 (C), 146.5 (CH), 169.8 (C).

**ESI-MS**: [M+H]^+^ calcd. for C_28_H_30_N_3_O_3_S = 488.2002 found 488.2003. (M.W. 715.6599)

***D*-WSAK:** (2R)-2-amino-3-(1H-indol-2-yl)-N-((S,E)-5-phenyl-1-(phenylsulfonyl)pent-1-en-3-yl)propanamide**.**

To a solution of (S)-N-Boc-Piperidine-2-carboxylic acid (211 mg, 0.92 mmol) in DMF (3 mL) was added EDC/HCl (177 mg, 0.92 mmol), HOBt (125 mg,0.92 mmol) and DIEA (0.6 mL, 3.1 mmol). After 10 min, (S)-3-benzenesulfonyl-1-phenethylallylamine trifluoroacetate (TFA•HphVSPh) (320 mg, 0.77 mmol) in DMF (3 mL) was added drop-wise. The reaction was stirred at RT for 12 h. The resulting solution was evaporated in vacuo to give a light yellow oil, which was diluted with aqueous NH_4_Cl (10 wt%) and extracted with EtOAc (3 x 50 mL). The combined organic extracts were dried over Na_2_SO_4_, filtered and concentrated in vacuo. Purification by flash column chromatography (silica gel; using 40% EtOAc in hexanes) provided the protected product as a white foam.

To a solution of this intermediate in CH_2_Cl_2_ (5 mL) at 0 °C was added trifluoroacetic acid (5 mL). The solution was stirred at RT overnight, then evaporated to dryness in vacuo and finally and freeze-dried yielding a white solid that was identified as the trifluoroacetic salt of the desired product (216 mg, 0.41 mmol, 52%).

**^1^H NMR (250 MHz, MeOD-*d_4_***): 1.39-1.49 (m, 1H), 1.58-1.66 (m, 1H), 2.13 (dt, *J* = 9.1, 6.6 Hz, 2H), 3.21-3.37 (m, 2H partial overlapping with the MeOD4 signal), 4.07 (t, *J* = 7.8 Hz, 1H), 4.39 (dd, *J* = 13.2, 5.6 Hz, 1H), 6.63 (dd, *J* = 15.1, 0.8 Hz, 1H), 6.83 (dd, *J* = 15.1, 5.4 Hz, 1H), 6.94 (d, *J* = 7.3 Hz, 2H), 7.03-7.15 (m, 4H), 7.19-7.22 (m, 3H), 7.30 (d, *J* = 8.0 Hz, 1H), 7.58-7.61 (m, 2H), 7.68 (t, *J* = 7.2 Hz, 1H), 7.86 (d, *J* = 7.6 Hz, 2H).

**^13^C NMR (MeOD-*d_4_*):** 29.0 (CH_2_), 32.6 (CH_2_), 35.8 (CH_2_), 51.2 (CH), 55.5 (CH), 108.2 (C), 112.8 (CH), 119.1 (CH), 120.3 (CH), 122.9 (CH), 125.4 (CH), 127.1 (CH), 128.4 (C), 128.7 (CH), 129.4 (CH), 129.5 (CH), 130.6 (CH), 132.3 (CH), 134.9 (CH), 138.2 (C), 141.7 (C), 141.9 (C), 146.7 (CH), 169.9 (C).

**ESI-MS**: [M+H]^+^ calcd. for C_28_H_30_N_3_O_3_S = 488.2002 found 488.1994. (M.W. 715.6599)

**Production of polyclonal DPAP3 antibodies:**

To raise antibodies against the N-terminal half of DPAP3, the sequence of rDPAP3-Nt was amplified by PCR using primer P1 (forward, has BamHI site) and P2 (reverse, has XhoI site) from pUC57-rDPAP3-Nt (Genewiz), which excludes the signal peptide coding region, and was ligated into the pGex-4T1 backbone (GE Healthcare Life Science) using BamHI and XhoI restriction enzymes resulting in the pGex-rDPAP3Nt plasmid for expression of GST-rDPAP3-Nt-His fusion protein in *E. coli*. To raise antibodies against the C-terminal half of DPAP3, the sequence or rDPAP3-Ct was amplified by PCR using primers P3 (forward, has BamHI site) and P4 (reverse, has NotI-site) from pUC57-rDPAP3-Ct (Genewiz) and ligated into the backbone after restriction digest with BamHI and NotI, resulting in the pGex-rDPAP3Ct plasmid for expression of GST-rDPAP3-Ct-His fusion protein in *E. coli*.

## Recombinant DPAP3 expression and purification for antibody production.

GST-rDPAP3-Nt-His (83,5 kDa) and GST-rDPAP3-Ct-His (83,7 kDa) were transfected into *E. coli* strain BL21 (DE3), where the expression of the target gene is controlled via isopropyl-1-thio-β-D-galactopyranoside. GST-rDPAP3-Nt-His and GST-rDPAP3-Ct-His fragments were purified from inclusion bodies under denaturing conditions. Inclusion bodies of the respective proteins were solubilized in BugBuster (Merck Millipore) and subsequently in lysis buffer (100 mM NaH_2_PO_4_∙H_2_O, 10 mM TrisHCl, 0.3 % SDS, 8 M Urea, pH 8.0) for 30 min at RT. Cleared supernatant was then dialyzed against lysis buffer without SDS using Slide-A-Lyzer Dialysis Cassettes (20 k MWCO, Thermo Scientific) and subsequently mixed with Ni-NTA agarose beads (Quiagen) in Buffer 1 (100 mM NaH_2_PO_4_, 10 mM TrisHCl, 20 mM imidazole, 8 M Urea, pH 8.0) for 2 h at RT. After washing with Buffer 1, proteins were eluted with Buffer 1 supplemented with 250 mM imidazole, and dialyzed against 100 mM NaH_2_PO_4_∙H_2_O, 10 mM TrisHCl, 200 mM NaCl, 4 M Urea, pH 8.0. Antibodies were raised in rat against purified GST-rDPAP3Nt-His and rabbit against GST-rDPAP3Ct-His by CovaLab (France).

**Supplementary References**

1. Mølgaard A, Arnau J, Lauritzen C, Larsen S, Petersen G, Pedersen J. The crystal structure of human dipeptidyl peptidase I (cathepsin C) in complex with the inhibitor Gly-Phe-CHN2. Biochem J. 2007;401: 645–650. doi: 10.1042/BJ20061389.

2. Turk D, Janjić V, Stern I, Podobnik M, Lamba D, Dahl SW, et al. Structure of human dipeptidyl peptidase I (cathepsin C): exclusion domain added to an endopeptidase framework creates the machine for activation of granular serine proteases. EMBO J. 2001;20: 6570–6582. doi: 10.1093/emboj/20.23.6570.

3. Klemba M, Gluzman I, Goldberg DE. A *Plasmodium* *falciparum* dipeptidyl aminopeptidase I participates in vacuolar hemoglobin degradation. J Biol Chem. 2004;279: 43000–43007. doi: 10.1074/jbc.M408123200.

4. Arastu-Kapur S, Ponder EL, Fonović UP, Yeoh S, Yuan F, Fonović M, et al. Identification of proteases that regulate erythrocyte rupture by the malaria parasite *Plasmodium* *falciparum*. Nat Chem Biol. 2008;4: 203–213. doi: 10.1038/nchembio.70.

5. Wang F, Krai P, Deu E, Bibb B, Lauritzen C, Pedersen J, et al. Biochemical characterization of *Plasmodium* *falciparum* dipeptidyl aminopeptidase 1. Mol Biochem Parasitol. 2011;175: 10–20. doi: 10.1016/j.molbiopara.2010.08.004.

6. Yang P-Y, Wang M, He CY, Yao SQ. Proteomic profiling and potential cellular target identification of K11777, a clinical cysteine protease inhibitor, in *Trypanosoma brucei*. Chem Commun (Camb). 2012;48: 835–7. doi: 10.1039/c1cc16178d.
