# Supplementary material for: Plasmodium falciparum dipeptidyl aminopeptidase 3 activity is important for efficient erythrocyte invasion by the malaria parasite
Source: PLoS Pathog. 2018 May 16;14(5):e1007031. doi: 10.1371/journal.ppat.1007031 (PMC5973627; doi:10.1371/journal.ppat.1007031)
Supplement: S1 Table — (DOCX) [file ppat.1007031.s002.docx]

| S1 Table. Data from all plaque assay experiments. | | | | | |
| --- | --- | --- | --- | --- | --- |
| **assay** | **clone/parasite line** | **RAP** | **Plaques total** | **plaques/well** | **ratio RAP/DMSO** |
| 1 | F8cKO | + | 62 | 0.517 | 0.069 |
| 1 | F8cKO | - | 902 | 7.517 |  |
| 1 | F3cKO | + | 105 | 0.875 | 0.121 |
| 1 | F3cKO | - | 871 | 7.258 |  |
| 2 | F8cKO | + | 69 | 0.575 | 0.069 |
| 2 | F8cKO | - | 1001 | 8.312 |  |
| 2 | F3cKO | + | 103 | 0.858 | 0.097 |
| 2 | F3cKO | - | 1063 | 8.858 |  |
| 2 | E7ctr | + | 1024 | 8.533 | 1.38 |
| 2 | E7ctr | - | 743 | 6.192 |  |
| 3 | F8cKO | + | 39 | 0.65 | 0.122 |
| 3 | F8cKO | - | 319 | 5.31 |  |
| 3 | A1cKO | + | 6 | 0.1 | 0.0214 |
| 3 | A1cKO | - | 281 | 4.683 |  |
| 3 | E7ctr | + | 217 | 3.616 | 0.904 |
| 3 | E7ctr | - | 240 | 4 |  |
| 3 | F8cKO+Mut_AMA1_ | + | 14 | 0.233 | 0.070 |
| 3 | F8cKO+Mut_AMA1_ | - | 200 | 3.333 |  |
| 3 | F8cKO+WT_AMA1_ | + | 64 | 1.066 | 0.810 |
| 3 | F8cKO+WT_AMA1_ | - | 79 | 1.316 |  |
| 4 | A1cKO | + | 6 | 0.1 | 0.0203 |
| 4 | A1cKO | - | 295 | 4.916 |  |
| 4 | F8cKO+Mut_DPAP3_ | + | 17 | 0.283 | 0.059 |
| 4 | F8cKO+Mut_DPAP3_ | - | 289 | 4.816 |  |
| 4 | F8cKO+WT_DPAP3_ | + | 24 | 0.4 | 0.320 |
| 4 | F8cKO+WT_DPAP3_ | - | 75 | 1.25 |  |
| 5 | A1cKO | + | 73 | 1.216 | 0.155 |
| 5 | A1cKO | - | 470 | 7.833 |  |
| 5 | F8cKO+WT_DPAP3_ | + | 51 | 0.85 | 0.273 |
| 5 | F8cKO+WT_DPAP3_ | - | 187 | 3.116 |  |
| 5 | F8cKO+Mut_DPAP3_ | + | 18 | 0.3 | 0.066 |
| 5 | F8cKO+Mut_DPAP3_ | - | 271 | 4.516 |  |
| 5 | F8cKO+WT_AMA1_ | + | 76 | 1.266 | 0.384 |
| 5 | F8cKO+WT_AMA1_ | - | 198 | 3.3 |  |
| 5 | F8cKO+Mut_AMA1_ | + | 27 | 0.45 | 0.104 |
| 5 | F8cKO+Mut_AMA1_ | - | 259 | 4.316 |  |
| 6 | A1cKO+WT_AMA1_ | + | 243 | 4.05 | 0.500 |
| 6 | A1cKO+WT_AMA1_ | - | 486 | 8.1 |  |
| 6 | A1cKO+Mut_AMA1_ | + | 71 | 1.183 | 0.125 |
| 6 | A1cKO+Mut_AMA1_ | - | 566 | 9.433 |  |
| 6 | A1cKO+WT_DPAP3_ | + | 49 | 0.816 | 0.130 |
| 6 | A1cKO+WT_DPAP3_ | - | 376 | 6.266 |  |
| 6 | A1cKO+Mut_DPAP3_ | + | 21 | 0.35 | 0.0405 |
| 6 | A1cKO+Mut_DPAP3_ | - | 519 | 8.65 |  |
| Related to Fig. 6A. | | | | | |
