# Supplementary material for: Plasmodium falciparum dipeptidyl aminopeptidase 3 activity is important for efficient erythrocyte invasion by the malaria parasite
Source: PLoS Pathog. 2018 May 16;14(5):e1007031. doi: 10.1371/journal.ppat.1007031 (PMC5973627; doi:10.1371/journal.ppat.1007031)
Supplement: S2 Table — (DOCX) [file ppat.1007031.s003.docx]

| S2 Table. List of primers used in this study. | | | |
| --- | --- | --- | --- |
| Primer ID | **Sequence** | **Descriptive name** | **Purpose of use** |
| P1 | GCGCGGATCCATCCCCGTGCACTGCCTGTCCCGT | 14_scDPAP3-Nterm-BamHI_F | Forward primer to amplify recodonized *dpap3* N-terminus with P2 excluding the signal peptide region (63nt), has BamHI site, to ligate into pGex4T1 backbone. |
| P2 | GCGCCTCGAGCCAAGCTCGGCGCGCCA | 15_scDPAP3-Nterm-XhoI_R | Reverse primer to amplify recodonized *dpap3* N-terminus with P1, has XhoI site to ligate into pGex4T1 backbone. |
| P3 | GCGCGGATCCGCCACCATGAAGCGC | 6_dsDPAP3C-BamHI_F | Forward primer to amplify recodonized *dpap3* C-terminal region with P4 to ligate into pGex-4T1 backbone, has BamHI site. |
| P4 | GCGCGCGGCCGCTTAGTGGTGGTGATGGTGATGACG | 7_dsDPAP3C-NotI_R | Reverse primer to amplify recodonized *dpap3* C-terminal region with P3 to ligate into pGex-4T1 backbone, has NotI site and stop-codon binds within 3xHis-repeat |
| P5 | GCGCGGATCCGTGATATTCCTGTTCACTGC | 10_cDPAP3Nterm-BamHI_F | Forward primer to amplify *dpap3* N-terminal region from gDNA 59nt downstream of start-codon with P6, has BamHI site, to ligate into pHH1 backbone for chimeric and tagged DPAP3. |
| P6 | GCGCCTGCAGGTTGTTGATGATCTTCTTCTTTTCATCATATAATTTATTATATTCATCTGCG | 11_cDPAP3Nterm-PstI_R | Reverse primer to amplify *dpap3* N-terminal region from gDNA 59nt downstream of start-codon with P5, has PstI site to ligate into pHH1 backbone for chimeric and tagged DPAP3. |
| P7 | GCGCCTCGAGGTCGACATGGTGAGCAAGGGCGAGGAGGAT | 20_mCherry-XhoI_F | Forward primer to amplify *mCherry* and 3’UTR with primer P8 to ligate into plasmid pHH1 for chimeric and tagged DPAP3 or exchange HA_3_ tag with *mCherry*, has XhoI and SalI site. |
| P8 | GCGCGCGGCCGCACTAGTCTACCCTGAAGAAGAAAAGTCC | 22_Pf3'UTR-NotI_R | Reverse primer to amplify *mCherry* and 3’UTR with primer P7 to ligate into plasmid pHH1 for chimeric and tagged DPAP3 or exchange HA_3_ tag with *mCherry*, has NotI and SpeI site. |
| P9 | GCGCGTTAACGGTACCGTGATATTCCTGTTCACTGCT | 16_cDNADPAP3-HpaI_F | Forward primer to amplify full length chimera *dpap3* with P10, has HpaI site to ligate into pHH1 backbone for chimeric and tagged DPAP3. |
| P10 | GCGCAGATCTCCCGGGCGGGTTTCCTTCTGCTTCACGAACA | 17_cDPAP3-BglII_R | Reverse primer to amplify full length chimera *dpap3* with P9, has BglII site to ligate into pHH1 backbone for chimeric and tagged DPAP3. |
| P11 | ACAAAATGGTTAACGGTACCGTGATATTCCTGTTCACTGCT | 48_INFUcDNANterm_F | Forward primer to amplify WT *dpap3* N-terminus with P12, has KpnI site to ligate into pHH1 for chimeric and tagged DPAP3 with *loxP* within region coding of Asn-stretch. |
| P12 | TGTCACCGGTAAataacttcgtataatgtatgctatacgaagttatGTTGTTATCGTTGTTGTCGTTG | 53_loxPinNNN-AgeI_R | Reverse primer to amplify WT *dpap3* N-terminus with P11, has AgeI site and *loxP* recognition region (lowercase), to ligate into pHH1 for chimeric and tagged DPAP3 with *loxP* within region coding of Asn-stretch. |
| P13 | ATTTACCGGTGACAACAACAACAACGTGC | 52_loxPinNNN-AgeI_F | Forward primer to amplify recodonized *dpap3* C-terminus with P14, has AgeI site to ligate into pHH1 for chimeric and tagged DPAP3 with *loxP* within region coding the Asn-stretch. |
| P14 | CTCGAGATCTCCCGGGCGGGTTTCCTTCTGCTTCACGAACA | 49_INFUscCterm_R | Forward primer to amplify recodonized *dpap3* C-terminus with P13, has SmaI site to ligate into pHH1 for chimeric and tagged DPAP3 with *loxP* within region coding the Asn-stretch. |
| P15 | GCGCGGTACCATGATCCTGATCTTCCAGCTGTTC | 28_scDPAP3Nt_F-KpnI | Forward primer to amplify recodonized *dpap3* N-terminus including coding region for signal peptide with primer P16, has KpnI site to ligate into pHH1 backbone for recodonized full length *dpap3.* |
| P16 | GCGCATCGATGTACTTGTAGAACAGGGA | 34_scDPAP3_OL_R-ClaI | Reverse primer to amplify recodonized *dpap3* N-terminus including coding region for signal peptide with primer P15, has ClaI site to ligate into pHH1 backbone for recodonized full length *dpap3.* |
| P17 | GCGCGTTAACATTAATGAGGTGTGTTGGGAAACAG | 30_AMA1-5’UTR_F-HpaI | Forward primer to amplify *ama1* promoter region with primer P18, has HpaI site to ligate into pHH1 backbone for episomal expression of recodonized DPAP3-HA. |
| P18 | GCGCGGTACCTTTTGTACAATTTATAACAAGTACA | 31_AMA1-5’UTR_R-KpnI | Reverse primer to amplify *ama1* promoter region with primer P17, has KpnI site to ligate into pHH1 backbone for episomal expression of recodonized DPAP3-HA. |
| P19 | GCGCGTTAACGACAAGGAAAGCTGCAGAGGAAG | 86_5'dpap3-HpaI_F | Forward primer to amplify *dpap3* promoter region (990 nt) with primer P20, has HpaI site to ligate into pHH1 backbone for episomal expression of recodonized DPAP3-HA. |
| P20 | GCGCGGTACCATCAGGGAAATTTATTTA | 87_5'dpap3-KpnI_R | Forward primer to amplify *dpap3* promoter region (990 nt) with primer P19, has KpnI site to ligate into pHH1 backbone for episomal expression of recodonized DPAP3-HA. |
| P21 | ATGATCCGTTGATACGTGTTCTT | 27_5'UTRPfDPAP3_F | Forward primer to test for integration or non-integration of chimeric pHH1-*dpap3* constructs with primer P22 (integration) or P23 (pre-integration), binds 278nt before *dpap3* start-codon. |
| P22 | GTAGAACAGGGAGTTGCGCTT | 2_scDPAP3C_R | Reverse primer to test for integration of chimeric pHH1-*dpap3* constructs with P21, binds 5’ end of recodonized *dpap3* C-terminus. |
| P23 | GAATGTGAAAGTTGATCATCTTGAGAC | 25_gDNA-DPAP3_R | Reverse primer to test for non-integration of chimeric pHH1-*dpap3* constructs with P21, binds 5’ end of WT *dpap3* C-terminus (30nt behind SSC region). |
| P24 | GTCCACAACATCATCGGAC | 60_Pb3UTR_F | Forward primer to test for non-excision with M13, binds within Pb3’UTR region. |
| P25 | CGCAGATGAATATAATAAATTATATGATGAA | 83_gDPAP3Nt_R | Forward primer to test for excision with SP6, binds prior of first *loxP* site. |
| SP6 | ATTTAGGTGACACTATAG | SP6 | Reverse primer to test for excision with P25, binds downstream of Pb3’UTR and the second loxP site |
| M13 | AGCGGATAACAATTTCACAC | M13_R | Reverse primer to test for non-excision with P24, binds downstream of Pb3’UTR and the second loxP site |
| CVO140 | GGACGGATCCATGACTGAATACAAACCAACTGTTCGCC | CVO140 | Forward primer to amplify puromycin N-acetyltransferase (pac), has BamHI site to ligate into pHH1 and exchange with *hdhfr,* kind gift from Christiaan van Ooij. |
| CVO141 | GGACAAGCTTTTAAGCACCTGGTTTGCGAGTCATGC | CVO141 | Reverse primer to amplify puromycin N-acetyltransferase (pac), has HindIII site to ligate into pHH1 and exchange with *hdhfr,* kind gift from Christiaan van Ooij. |
| II-DPAP3Ct-F | GCACGCTCGAGAAAGTTCATTAATGAGTCAAGATG | II-DPAP3Ct-F | Forward primer to amplify DPAP3-Ct, has XhoI site to ligate into pPM2GT. |
| II-DPAP3Ct_R | GCACGCCTAGGTGTTTCTTTTTGTTTAACAAACAAGT | II-DPAP3Ct_R | Reverse primer to amplify DPAP3-Ct, has AvrII site to ligate into pPM2GT. |
| II-inte_F | CAATTAATACTATGTGATATATTCAACC | II-inte_F | Forward primer to test for integration of DPAP3-GFP construct or WT *dpap3* locus, binds upstream of HR. |
| II-inte_R | CCTCTCCACTGACAGAAAATTCGTGCC | II-inte_R | Reverse primer to test for integration of DPAP3-GFP construct (1.4kb), binds within GFP sequence. |
| II-wt_R | ATTACTATCACCATTATCCTTATTAT | II-wt_R | Reverse primer to test for WT *dpap3* locus (0.95kb), binds within HR. |
