# Supplementary material for: Plasmodium falciparum dipeptidyl aminopeptidase 3 activity is important for efficient erythrocyte invasion by the malaria parasite
Source: PLoS Pathog. 2018 May 16;14(5):e1007031. doi: 10.1371/journal.ppat.1007031 (PMC5973627; doi:10.1371/journal.ppat.1007031)
Supplement: S3 Table — (DOCX) [file ppat.1007031.s004.docx]

| S3 Table. List of all primary antibodies used for WB and IFA studies. | | | | |
| --- | --- | --- | --- | --- |
| **Name** | **Species** | **WB / IFA** | **Dilution Factor** | **Comments** |
| mAb X509 | human | IFA | culture supernatant | Antibody recognizes epitope within MSP1_42_ fragment [1]. |
| mAb 89.1 | mouse | WB | 1:2000 | Antibody recognizes epitope in MSP1_83_ fragment [2]. |
| AMA1 | mouse | WB | 1:3000 | Polyclonal antiserum raised against recombinant PfAMA1 [3]. |
| SUB1 | rabbit | WB | 1:1000 | Polyclonal antiserum raised against recombinant PfSUB1 [4]. |
| DPAP3-Nt | rat | WB | 1:2000 | Polyclonal antiserum raised against recombinant PfDPAP3_18-454_.  Not suitable for IFA studies. |
| DPAP3-Ct | rabbit | WB | 1:4000 | Polyclonal antiserum raised against recombinant PfDPAP3_454-946_.  Not suitable for IFA studies. |
| mCherry | mouse | WB / IFA | 1:1000 | Purchased from abcam, clone 1C51. |
| mCherry | rat | WB/ IFA | 1:1000 | Purchased from Invitrogen, clone 16D7. |
| HSP70 | rat | WB | 1:1000 | Purchased from Cell Signaling technology, clone 6B3. |
| HA | rat | WB / IFA | 1:2000 | Purchased from Roche, clone 3F10. |
| SUB1 | mouse | IFA | Culture supernatant | Monoclonal, clone NIMP-M7 [5]. |
| SERA5 | rabbit | WB | 1:2000 | Polyclonal [6]. |
| AMA1 | mouse | IFA | 1:1000 | Clone 4G2, kindly provided by Dr. Jean-Francois Dubrenetz and Dr Alan Thomas [7,8]. |
| AMA1 | rabbit | IFA | 1:500 | 1DI-III, polyclonal antiserum raised against recombinant AMA1 fragment [3]. |
| RON4 | mouse | IFA | 1:1000 | Monoclonal 24C6, kindly provided by Dr Jean Francoise Dubremetz [9]. |
| RopH2 | mouse | IFA | 1:500 | Monoclonal 61.3, kindly provided by Dr Anthony Holder [10]. |
| EBA175 | rat | IFA | 1:500 | Kindly provided by Dr Anthony Holder (MR4). |
| Gap45 | rabbit | IFA | 1:1000 | Kindly provided by Dr Anthony Holder [11]. |
| GFP | rabbit | IFA | 1:1000 | Purchased from Invitrogen, polyclonal. |
| HA | mouse | IFA | 1:500 | Purchased from Covance, clone 16B12. |
| BiP | Rat | WB | 1:3000 | Kindly provided by Dr Anthony Holder ]. |
| References:  1. Blackman MJ, Ling IT, Nicholls SC, Holder AA. Proteolytic processing of the *Plasmodium falciparum* merozoite surface protein-1 produces a membrane-bound fragment containing two epidermal growth factor-like domains. Mol Biochem Parasitol. 1991;49: 29–33.  2. Holder AA, Freeman RR. Biosynthesis and processing of a *Plasmodium falciparum* schizont antigen recognized by immune serum and a monoclonal antibody. J Exp Med. 1982;156: 1528–38. doi: 10.1084/jem.156.5.1528.  3. Collins CR, Withers-Martinez C, Hackett F, Blackman MJ. An inhibitory antibody blocks interactions between components of the malarial invasion machinery. PLoS Pathog. 2009;5: e1000273. doi: 10.1371/journal.ppat.1000273.  4. Blackman MJ, Fujioka H, Stafford WH, Sajid M, Clough B, Fleck SL, et al. A subtilisin-like protein in secretory organelles of *Plasmodium falciparum* merozoites. J Biol Chem. 1998;273: 23398–409. doi: 10.1074/jbc.273.36.23398.  5. Withers-Martinez C, Saldanha JW, Ely B, Hackett F, O'Connor T, Blackman MJ. Expression of recombinant *Plasmodium* *falciparum* subtilisin-like protease-1 in insect cells. Characterization, comparison with the parasite protease, and homology modeling. J Biol Chem. 2002;277: 29698–29709. doi: 10.1074/jbc.M203088200  6. Stallmach R, Kavishwar M, Withers-Martinez C, Hackett F, Collins CR, Howell SA, et al. *Plasmodium* *falciparum* SERA5 plays a non-enzymatic role in the malarial asexual blood-stage lifecycle. Mol Microbiol. 2015;96: 368–87. doi: 10.1111/mmi.12941.  7. Kocken CHM, Withers-Martinez C, Dubbeld MA, van der Wel A, Hackett F, Valderrama A, et al. High-level expression of the malaria blood-stage vaccine candidate *Plasmodium* *falciparum* apical membrane antigen 1 and induction of antibodies that inhibit erythrocyte invasion. Infect Immun. 2002;70: 4471–6. doi: 10.1128/IAI.70.8.4471-4476.2002.  8. Kocken CH, van der Wel AM, Dubbeld MA, Narum DL, van de Rijke FM, van Gemert GJ, et al. Precise timing of expression of a Plasmodium falciparum-derived transgene in *Plasmodium* *berghei* is a critical determinant of subsequent subcellular localization. J Biol Chem. 1998;273: 15119–24. doi: 10.1074/jbc.273.24.15119.  9. Roger N, Dubremetz JF, Delplace P, Fortier B, Tronchin G, Vernes A. Characterization of a 225 kilodalton rhoptry protein of *Plasmodium* *falciparum*. Mol Biochem Parasitol. 1988;27: 135–41. doi: 10.1016/0166-6851(88)90033-3.  10. Holder AA, Freeman RR, Uni S, Aikawa M. Isolation of a *Plasmodium* *falciparum* rhoptry protein. Mol Biochem Parasitol. 1985; 14: 293–303. doi: 10.1016/0166-6851(85)90057-X.  11. Green JL, Rees-Channer RR, Howell SA, Martin SR, Knuepfer E, Taylor HM, et al. The motor complex of *Plasmodium* *falciparum*: phosphorylation by a calcium-dependent protein kinase. J Biol Chem. 2008;283: 30980–9. doi: 10.1074/jbc.M803129200.  12. Kuepfer E, Suleyman O, Dluzewski AR, Strachil U, O’Keefe AH, Ogun SA, et.al. RON12, a novel *Plasmodium*-specific rhoptry neck protein important for parasite proliferation. Cell Microbiol. 2014;16: 657-72. doi: 10.111/cmi.12181. | | | | |
