# Supplementary figures and images for: Plasmodium falciparum dipeptidyl aminopeptidase 3 activity is important for efficient erythrocyte invasion by the malaria parasite
Source: PLoS Pathog. 2018 May 16;14(5):e1007031. doi: 10.1371/journal.ppat.1007031 (PMC5973627; doi:10.1371/journal.ppat.1007031)

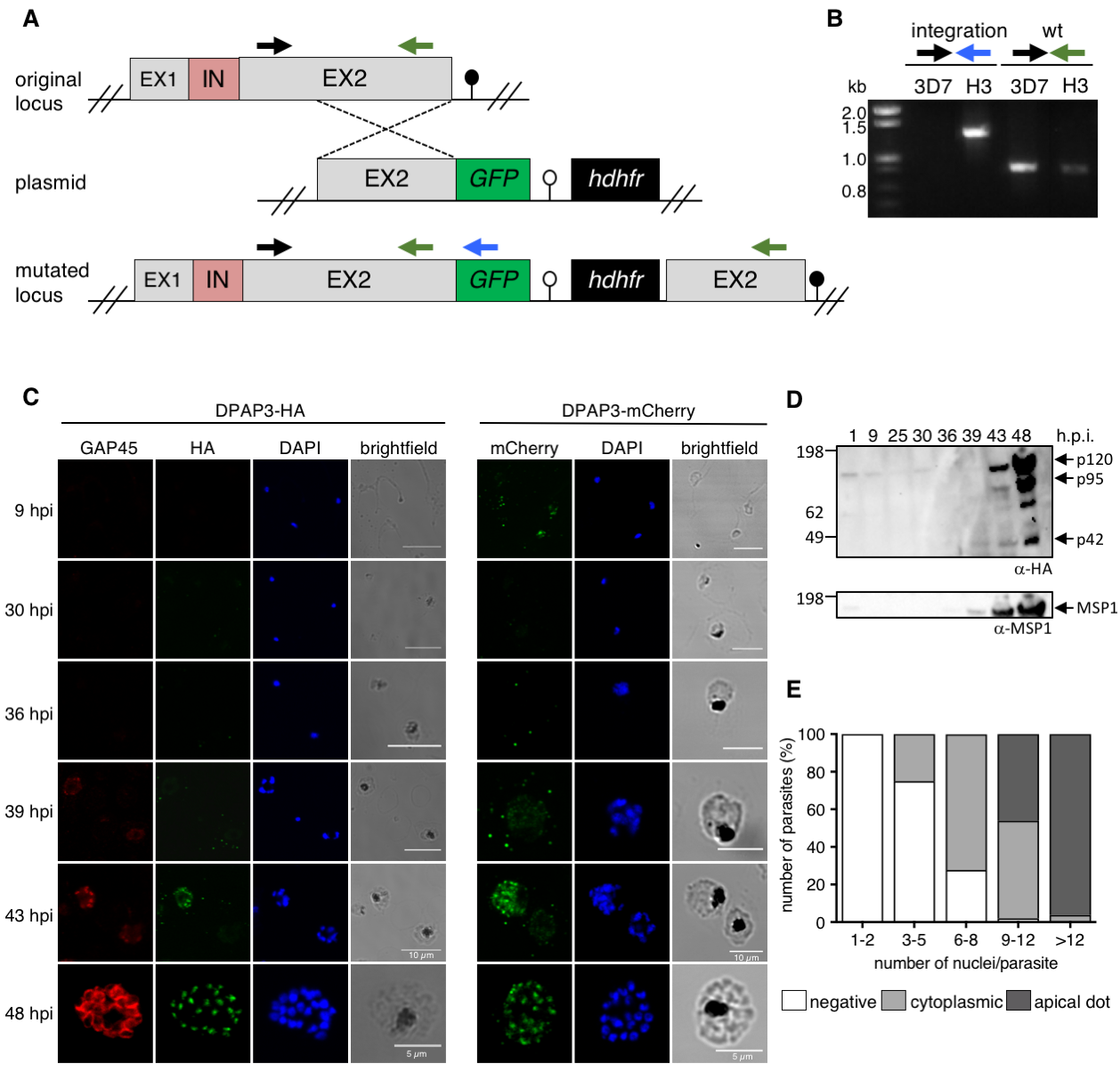

Supplement: S1 Fig — (Related to Fig 1) (A) Schematic representation of the dpap3 recombinant genetic locus. The endogenous locus (original locus) harbours two exons (EX1 and EX2 grey boxes), an intron (pink box; IN) and the 3’ regulatory sequence (black circle). The plasmid used to GFP-tag DPAP3 is made of a C-terminal homology region containing part of EX2 (1 kb), followed by a gfp sequence (0.7 kb, green box; GFP), a hsp86 3’ regulatory sequence (0.9kb, white circle), and the hdhfr resistance cassette (2 kb, black box). After single homologous recombination, the mutated locus harbours the dpap3-GFP sequence, and the truncated endogenous EX2 locus is displaced. (B) Integration efficiency at the dpap3 locus assessed by PCR on genomic DNA using the II-inte_F forward primer (black arrows) and the II-wt_R (orange arrows) or II-inte_R (blue arrow) reverse primers for the endogenous or modified locus, respectively. Primer binding sites are indicated in A. (C) IFA of parasites collected 9–48 h.p.i. from DPAP3-HA and DPAP3-mCh were fixed and stained with mouse anti-GAP45 (red) and rat anti-HA (left panel) or rabbit anti-mCherry (right panel) antibodies (both green). DNA was stained with DAPI (blue). IFA was analysed by confocal microscopy. Scale bar: 10 μm (9–43 h.p.i.) and 5 μm (48 h.p.i.). (D) WB analysis of DPAP3-HA parasite lysates harvested at different h.p.i. DPAP3-HA was detected using an anti-HA antibody. An MSP1 antibody was used to confirm that the low level of DPAP3 observed at ring and trophozoite stages is not due to schizont contamination in our samples. (E) Quantification of DPAP3-mCh parasites showing negative (white bar), cytoplasmic (grey bar) or apical (black bar) DPAP3 staining during schizogony (36 to 48h.p.i.). Schizont maturity was assigned based on the number of nuclei per iRBC. Quantification of DPAP3-HA parasites is shown in Fig 1G. (TIF) [file ppat.1007031.s005.tif]

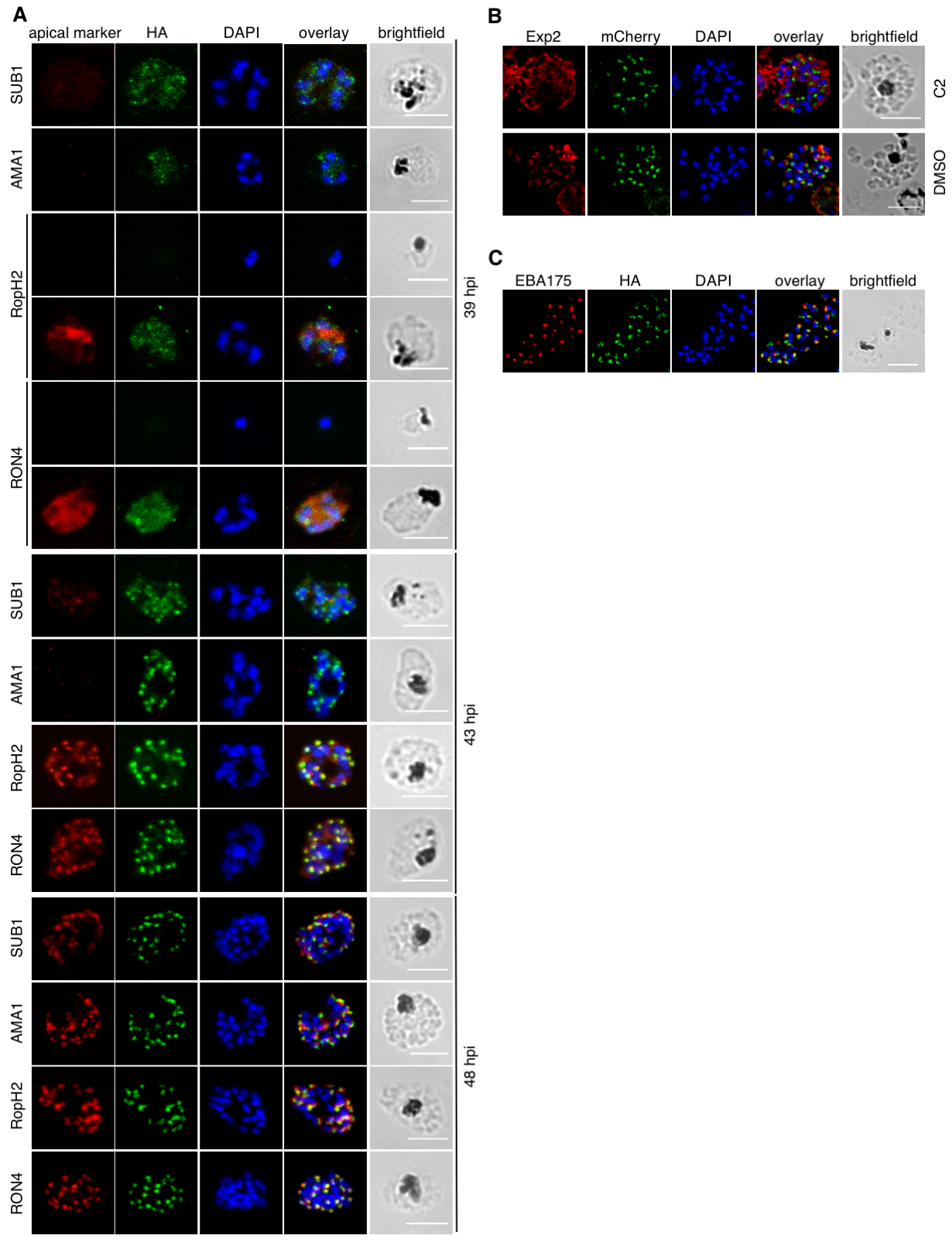

Supplement: S2 Fig — (Related to Fig 1F) (A) DPAP3-HA parasites collected 39–48 h.p.i. were fixed and stained with mouse anti-SUB1, rabbit anti-AMA1, mouse anti-RopH2, mouse anti-RON4 (all red), and rat anti-HA (green). For the 39 h.p.i. time point, we also show images of iRBCs that were lagging behind in development, i.e. containing only one nucleus. These images were collected from the same slides as the one of schizonts shown underneath and indicate that the diffuse staining observed in early schizonts is not due to background fluorescent signal. (B) IFA of DPAP3-mCh C2-arrested (upper panel) or rupturing (DMSO, lower panel) schizonts that were fixed 48 h.p.i. and stained with mouse anti-Exp2 (red) and rat anti-mCherry (green). (C) IFA of DPAP3-HA schizonts fixed 48 h.p.i. and stained with rat anti-EBA175 (red) and mouse anti-HA (green). (A-C) DNA was stained with DAPI (blue); scale bar: 5 μm. All IFAs were analysed by confocal microscopy. (TIF) [file ppat.1007031.s006.tif]

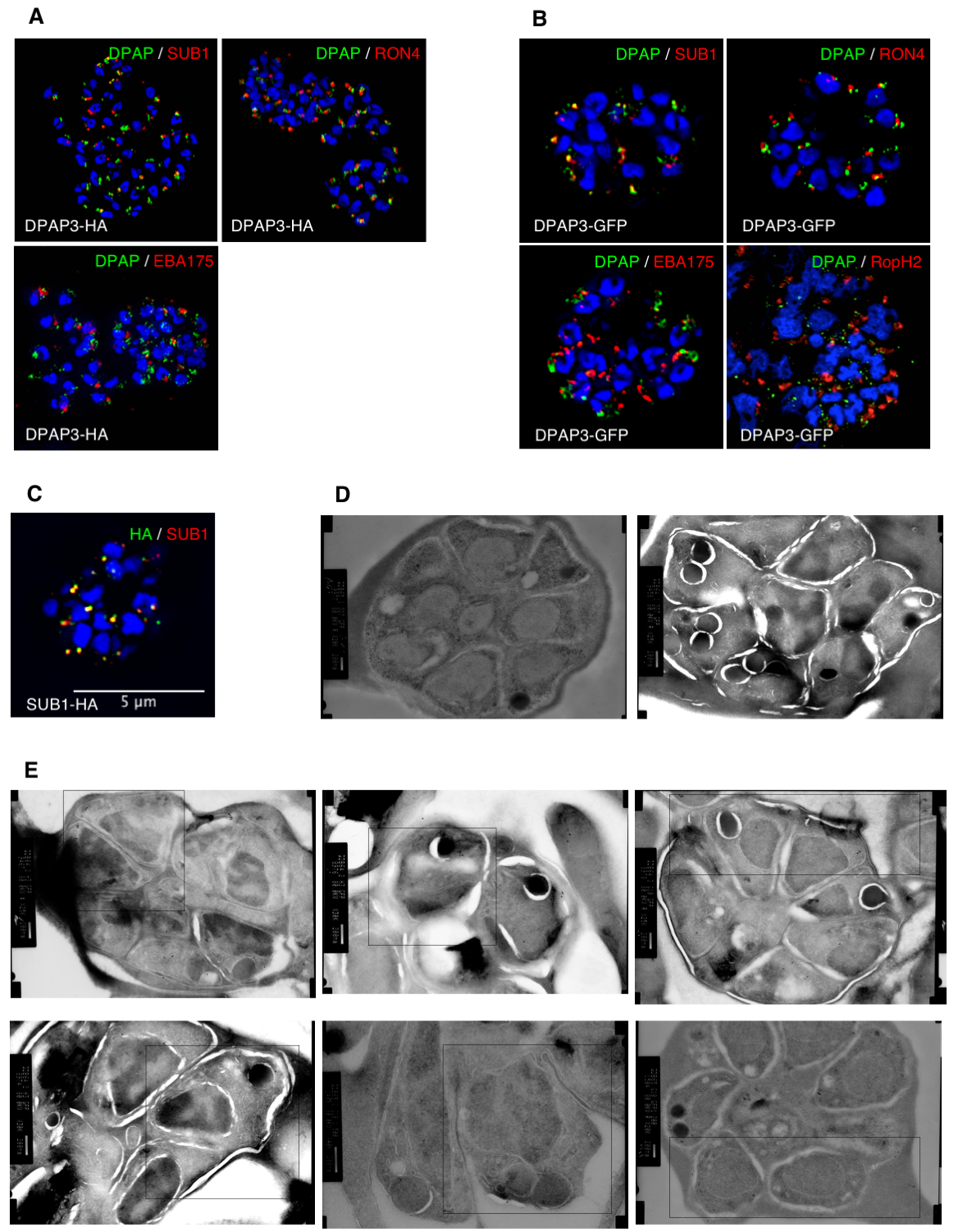

Supplement: S3 Fig — (Related to Figs 1H and 2D) (A) IFA of DPAP3-HA C2-arrested schizonts stained with rat anti-HA (green) and mouse anti-SUB1, mouse anti-RON4 and rat anti-EBA175 (all red). (B) Same IFA as in A but for DPAP3-GFP parasites. For this line staining with mouse anti-RopH2 (red) is also shown. DPAP3-GFP as well as DPAP3-HA forms small dot like structures at the apical pole that do not colocalize with any of the used apical marker proteins. (C) IFA of a late schizont from a SUB1-HA line (3D7SUB1-HA3)[10] was used as a control for colocalization at the apical pole using SIM. Parasite was fixed and stained with mouse anti-SUB1(red) and rat anti-HA (green). (A-C) DNA was stained with DAPI (blue); scale bar: 5 μm. All IFAs were analysed by SIM. Overlay of the staining is shown. (D) IEM sections of 3D7 schizonts stained with rabbit anti-GFP and colloidal gold-conjugated anti-rabbit antibodies. No significant unspecific labelling was observed on the 3D7 control line. (E) IEM sections corresponding to the uncropped images shown in Fig 2D. Dotted rectangles delineate the cropped images shown in Fig 2D. (D-E) Scale bar: 200 nm. (TIF) [file ppat.1007031.s007.tif]

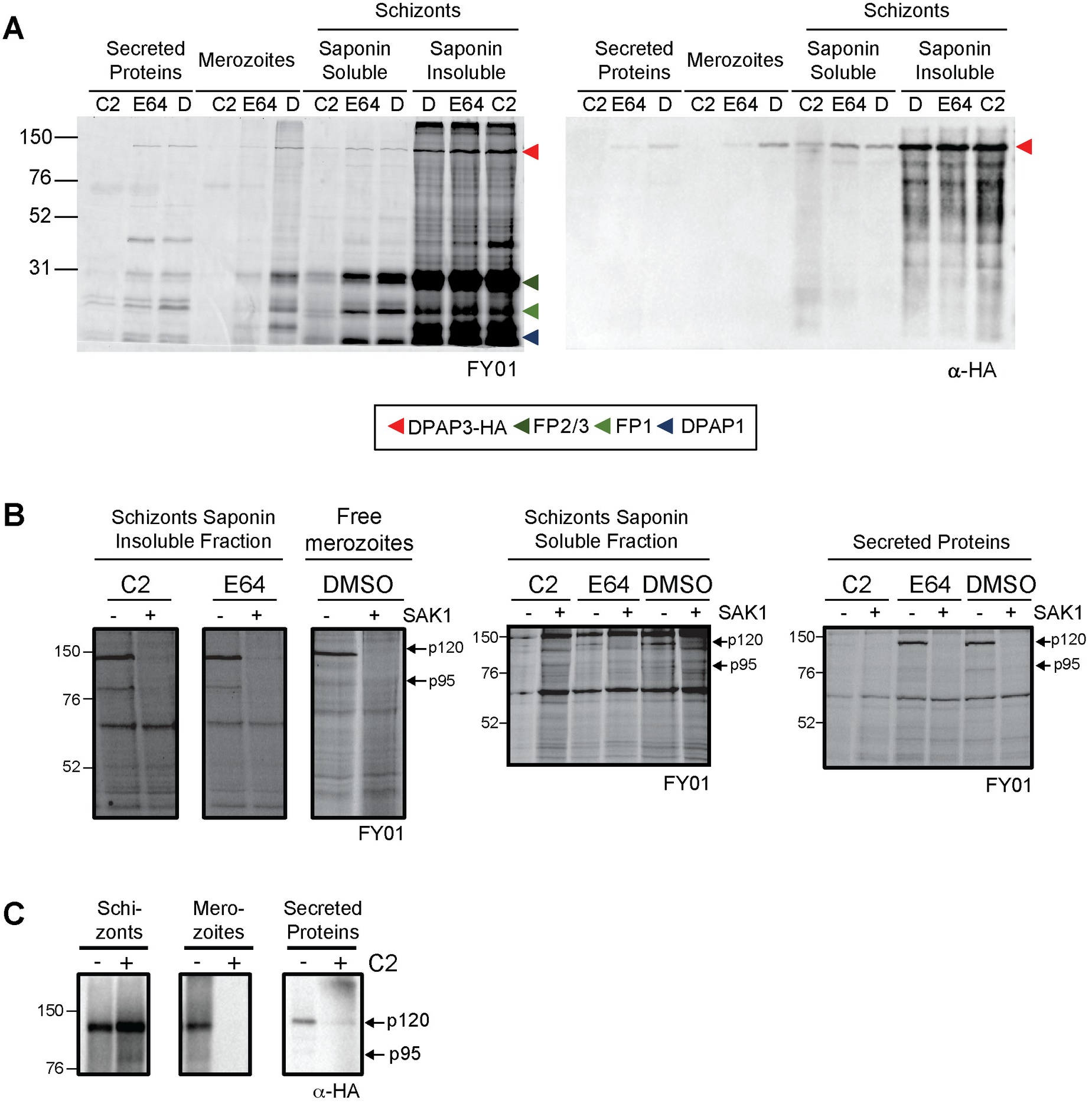

Supplement: S4 Fig — (Related to Fig 2) (A) WB analysis showing that the FY01-labelled band at 130kDa correspond to DPAP3-HA. C2-arrested schizonts were either left on C2, treated with E64 after C2 wash out, or allowed to egress for 1h in the presence of FY01 (Same samples as in Fig 2A). Parasite pellets from free merozoites and schizonts (insoluble fraction obtained after saponin lysis), proteins precipitated from the culture supernatant, and PV and RBC cytosol components (soluble saponin fraction), were run on a SDS-PAGE. DPAP3 labelling by FY01 can be observed as a fluorescent band at 130kDa, which correspond to the band identified by WB using an anti-HA antibody. Note that FY01 is also able to label other papain-fold cysteine proteases such as the falcipains (FP1-3) or DPAP1 (indicated by arrowheads). (B) Rupturing (DMSO) and C2- or E64-arrested 3D7 schizonts were labelled under intact conditions with FY01 in the presence or absence of the DPAP3 inhibitor SAK1. Proteins secreted in the culture supernatant, free merozoites, and the soluble and insoluble fractions of saponin lysed schizonts, were run on an SDS-PAGE gel. Fluorescent bands at 130 and 100 kDa that disappear in the presence of SAK1 correspond to the p120 and p95 forms of DPAP3-HA. The small proportion of p95 DPAP3 is likely produced after parasite lysis. (C) C2-arrested schizonts (DPAP3-HA line) were either left on C2 or allowed to egress for 30 min after C2 wash out. Unruptured schizonts, free merzoites, and proteins secreted in the culture supernatant were collected, and the presence of DPAP3-HA in each fraction visualized by WB using an anti-HA antibody. (TIF) [file ppat.1007031.s008.tif]

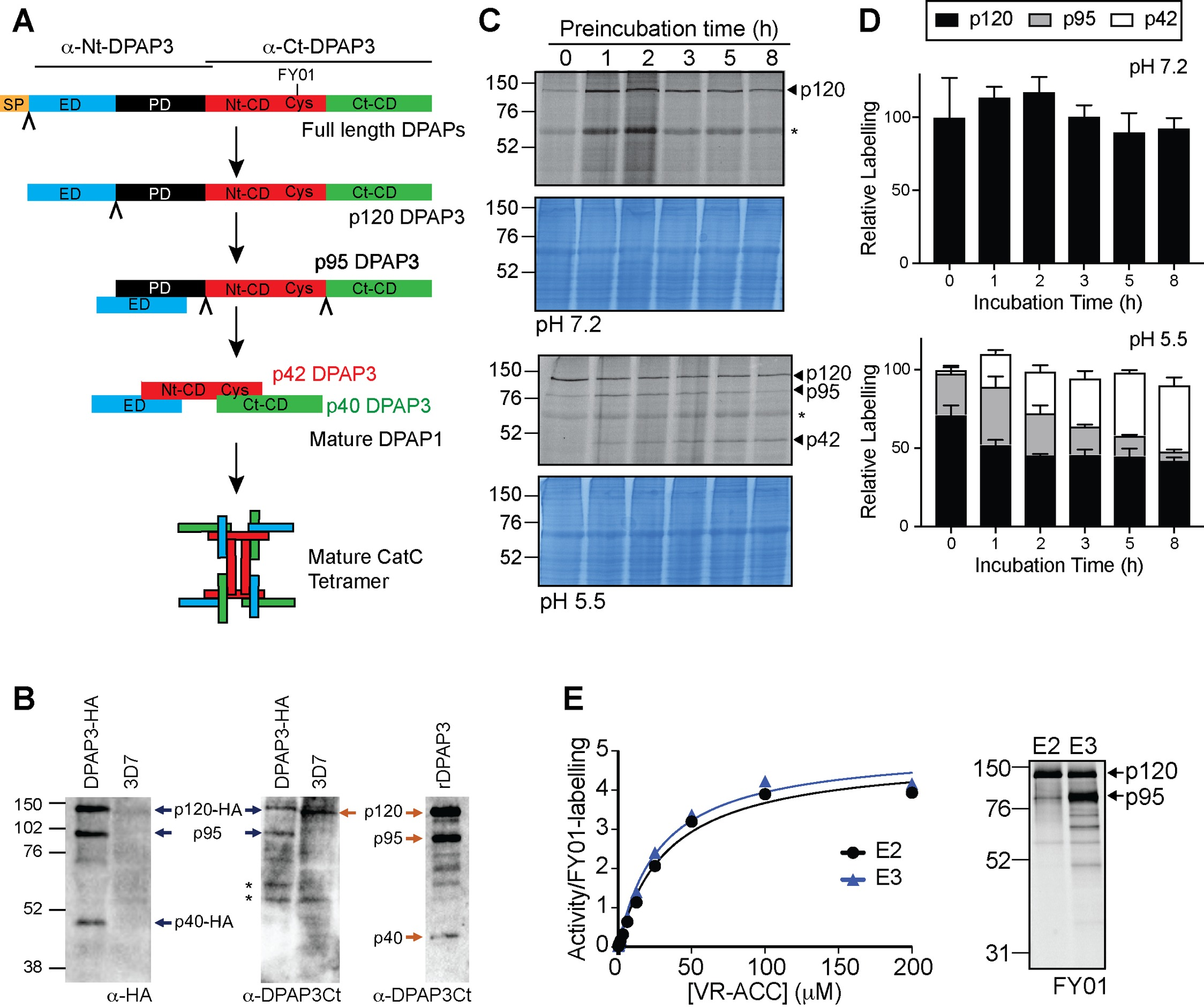

Supplement: S5 Fig — (Related for Fig 3) (A) Schematic representation of DPAPs processing. The signal peptide (SP), exclusion domain (ED), prodomain (PD), and the N- and C-terminal portions of the catalytic domain (Nt-CD and Ct-CD) are shown in different colours. Cleavage sites for removal of the SP and PD, and for processing of the CD are indicated by chevrons. A representation of fully processed monomeric DPAP1 and tetrameric CatC are shown. The predominant p120 DPAP3 active form is produced after removal of the SP. We propose that the p95 DPAP3 active form is produced after cleavage of the ED. The regions recognized by anti-Nt-DPAP3 and anti-Ct-DPAP3 antibodies, and the position of the catalytic Cys covalently modified by FY01 are shown above the full-length protein scheme. (B) Western blot analysis of DPAP3 processing in schizonts. Supernatant of insect cell cultures expressing rDPAP3, lysates of DPAP3-HA schizonts, and 3D7 mature schizonts directly boiled into loading buffer without previous lysis were analysed by WB using the α-DPAP3Ct, and with α-HA after stripping the same blot. Lysis of schizonts before addition of loading buffer results in processing of DPAP3 from the p120 form to the p95 and p40 forms (N- and C-terminal portion of the CD, respectively). However, if schizonts are directly boiled in loading buffer, only the physiologically relevant p120 form is observed (see also Fig 1). Note that the p120 and p95 forms obtained from rDPAP3 are very similar to the ones that are observed in parasite lysates. Asterisks indicate non-specific labelling by α-DPAP3Ct. (C) Representative data showing DPAP3 processing is an artefact of parasite lysis. Merozoite lysates were incubated for several hours under neutral (PBS, pH 7.2) or acidic (acetate buffer, pH 5.5) conditions before adding 1 μM of FY01 for 1 h. Under acidic conditions, we observed a time dependent processing of the p120 form to the p95 and p42 forms. Coomassie staining of the gel is shown as a loading control. This exper [file ppat.1007031.s009.tif]

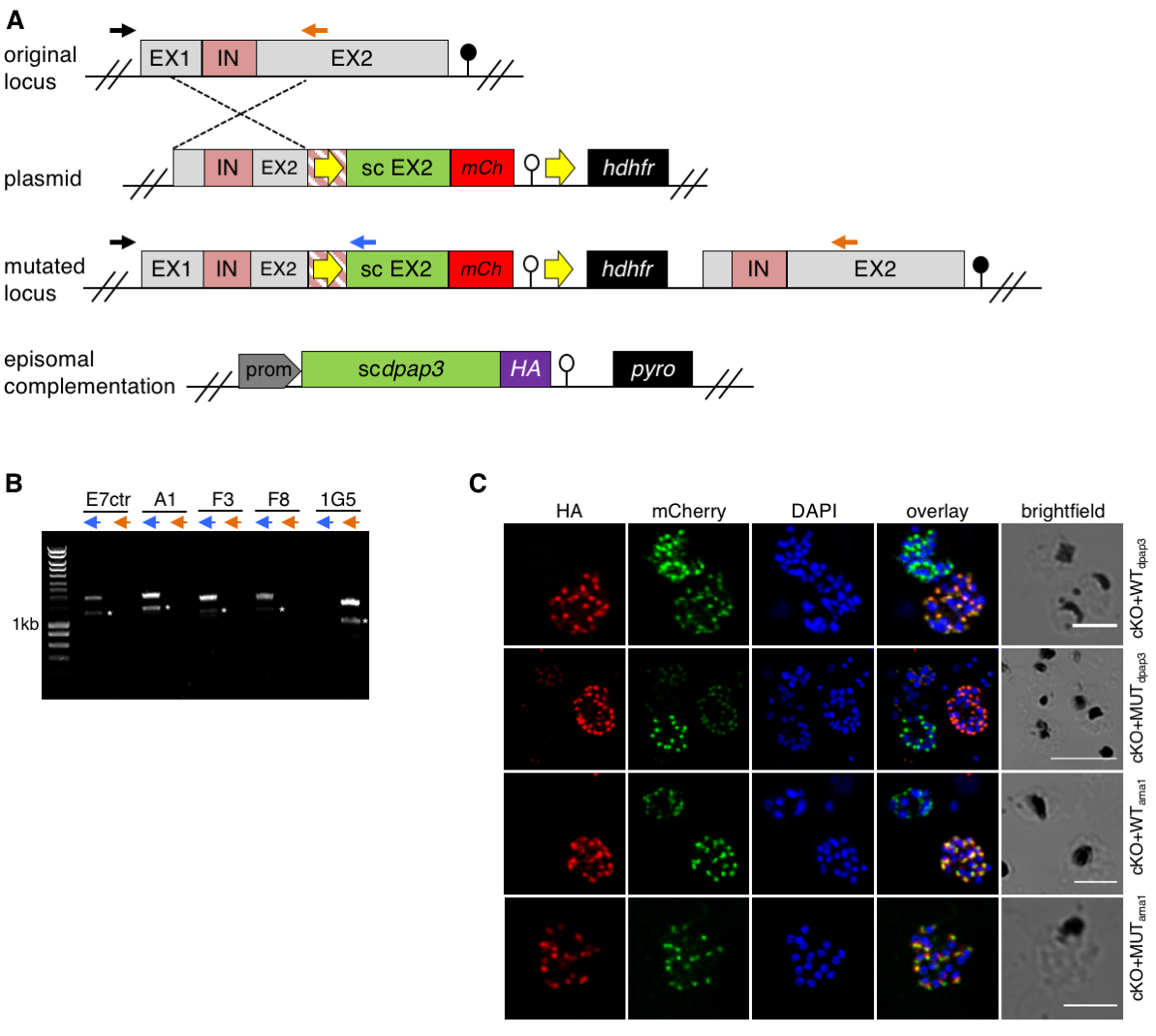

Supplement: S6 Fig — (Related to Figs 4 and 5) (A) Schematic representation of the dpap3 recombinant genetic locus. The endogenous locus is represented as described in S1A Fig. The plasmid used to integrate two loxP sites into the ORF is made of a homology region containing the DPAP3 intron and part of its two exons (0.2 kb of EX1 and 0.8 kb of EX2) and the rest of EX2 as a recodonized sequence (1.8 kb, sc EX2; green box) containing a loxP site (yellow arrow) at its 5’ end, either as a loxPint (0.1 kb, box with white-pink stripes and yellow arrow, F8cKO and F3ckO) or integrated in the ORF for A1cKO (not shown here but represented in Fig 4A). This is followed by an mCherry sequence (0.7 kb, red box; mCh), a P. berghei 3’ regulatory sequence (0.8 kb, white circle), the 2nd loxP site (yellow arrow, clone E7ctr only harbours this loxP site), and the hdhfr resistance cassette (2 kb, black box). After single homologous recombination, the mutated locus harbours the chimeric dpap3-loxP-mCherry sequence, and the truncated endogenous WT dpap3 locus is displaced. The constructs used for episomal complementation of DPAP3 contain a promoter region (dark grey box; prom), which was either the 5’ regulatory sequence of ama1 (1.5 kb) or dpap3 (0.9 kb). This was followed by a recodonized version of the dpap3 gene (green box, scdpap3) fused to a HA3 tag (purple box), a P. berghei 3’ regulatory sequence, and a pyromycin resistance cassette (2.2 kb, black box; pyro), which is driven by the cam 5’ regulatory sequence. (B) Integration efficiency at the dpap3 locus in transgenic parasites was assessed by PCR on genomic DNA using primers P21 and P23 for the endogenous locus, and P21 and P22 for mutated locus. P21, P22, and P23 binding sites are indicated in (A) by black, blue and orange arrows, respectively. PCR was performed on genomic DNA purified from the E7ctr, A1cKO, F3cKO, and F8cKO parasite lines. 1G5 parasites were used as a negative control for integration. The predicted size of the DNA fragment amplif [file ppat.1007031.s010.tif]

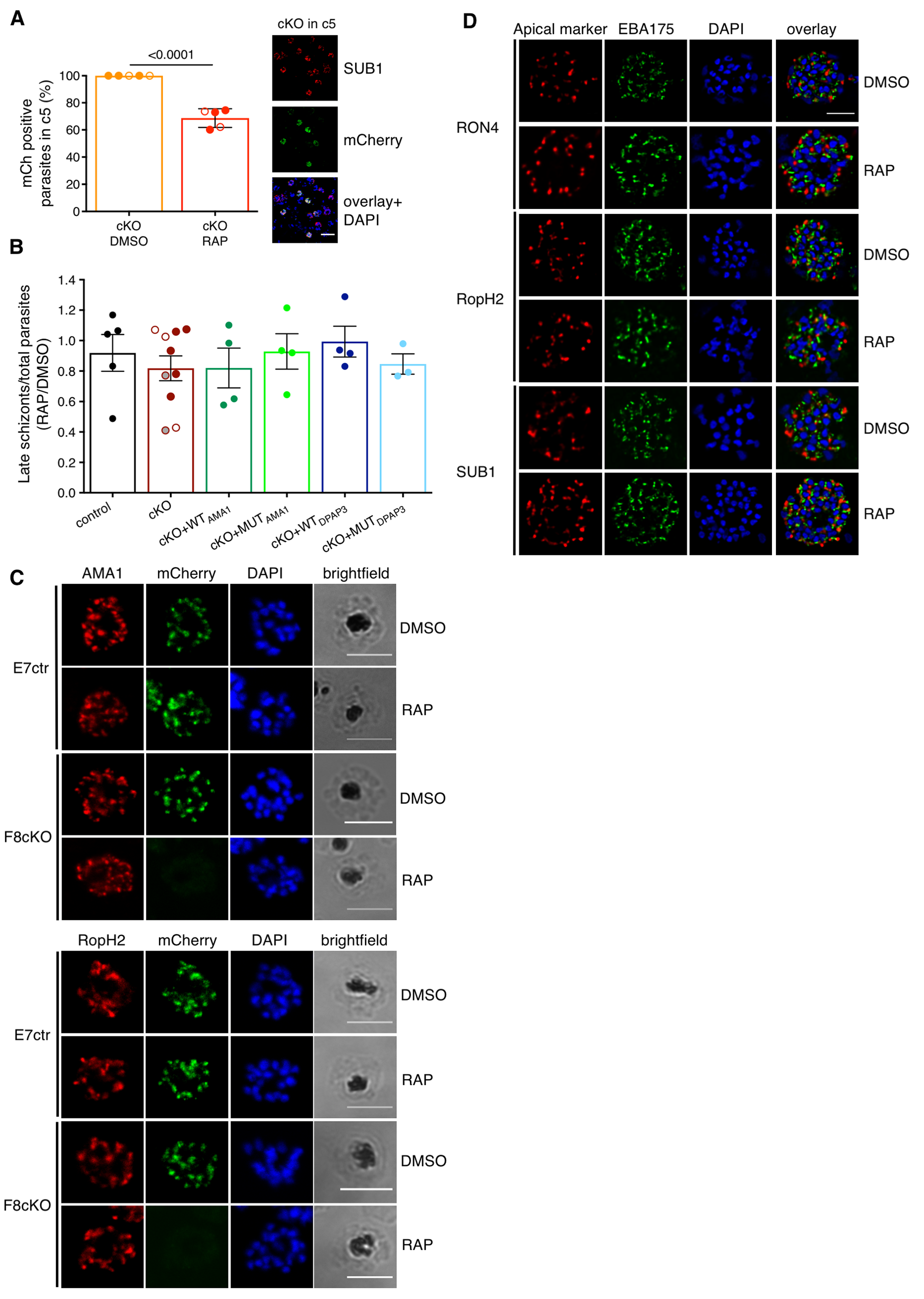

Supplement: S7 Fig — (Related to Fig 6) (A) F8cKO and A1cKO parasites were either treated with DMSO or RAP, and schizonts purified on cycle 5 (c5) after treatment, fixed, and stained with rat anti-mCherry (green), mouse anti-SUB1 (red), and DAPI (blue). The percentage of mature schizonts that were positive for mCherry staining was calculated. SUB1 staining was used as a marker of schizont maturity. As expected all mature schizonts in DMSO treated parasites were mCherry positive, but only 40% were mCherry negative five cycles after RAP treatment, indicating that non-excised parasites grow faster than excised ones. Each circle indicates a biological replicate with filled and empty circles corresponding to the F8cKO and A1cKO lines, respectively. The significance p-value calculated with a Student’s t test is show. (B) Quantification of late schizont development in cKO and complementation lines. After DMSO or RAP treatment of the E7ctr, F8cKO (full circles), F3cKO (grey circles), A1cKO (empty circles), or F8cKO+WT/MUTama1/dpap3 (full circles) lines at ring stage, schizonts were collected at 48 h.p.i., fixed, and stained with anti-mCherry and anti-SUB1 antibody as a marker of schizont maturity. The amount of mature schizonts was quantified and plotted as the ratio between RAP and DMSO treatment. Each circle corresponds to a different biological replicate. The E7ctr lines was used as a negative control of excision. No significant differences between RAP and DMSO treatment was observed for any of the parasite lines using a Student’s t test. (A-B) A minimum of 100 parasites was analysed per biological replicate. (C) AMA1 and RopH2 are expressed and localize to the apical pole in DPAP3-KO parasites. F8cKO and E7ctr parasites were treated with RAP or DMSO, fixed at 48 h.p.i., and stained with rat anti-mCherry (green) and rabbit anti-AMA1 or mouse anti-RopH2 (both red). No differences in the localization or appearance of both apical markers could be found between RAP- and DMSO-treated parasites. ( [file ppat.1007031.s011.tif]

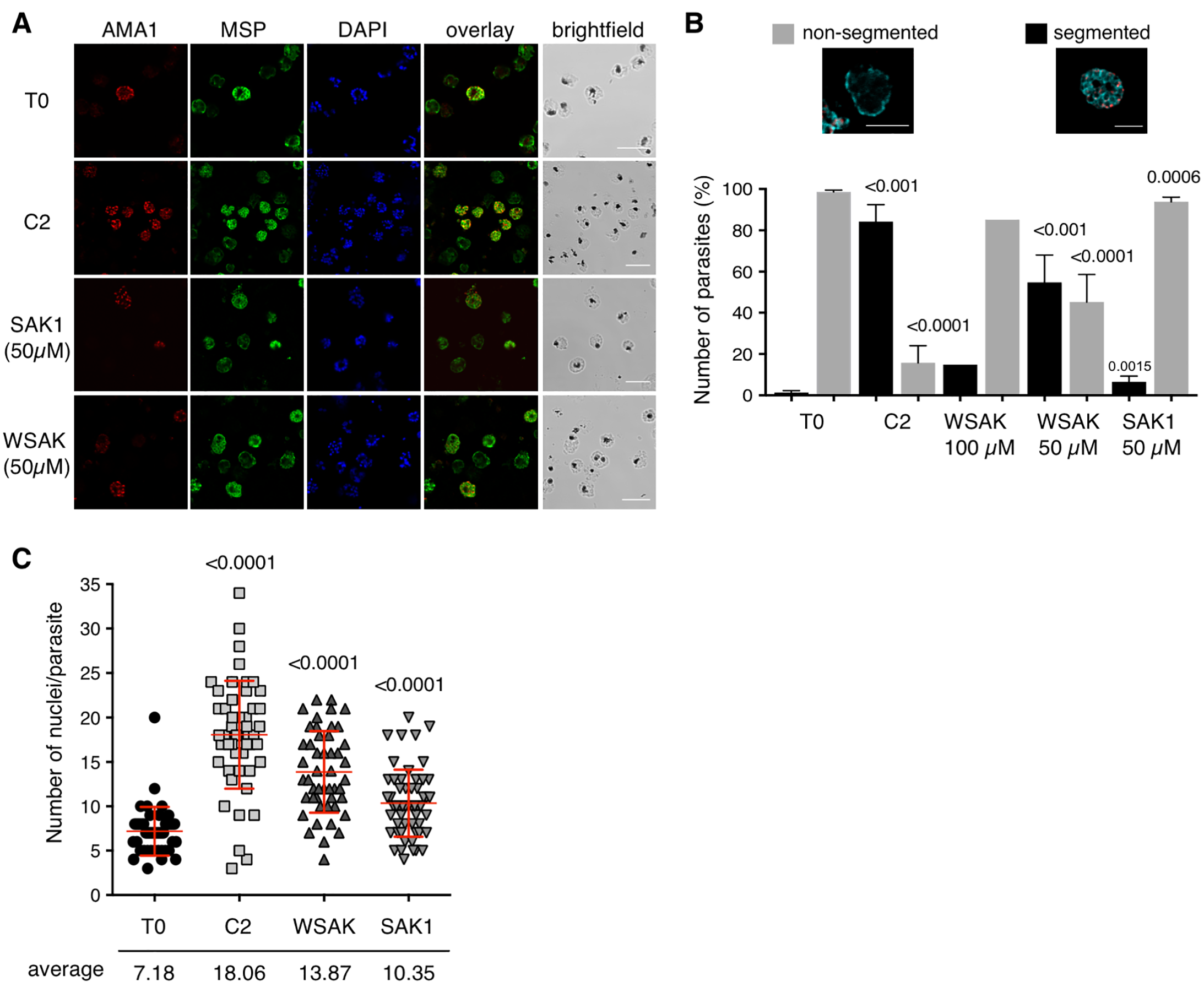

Supplement: S8 Fig — (Related to Fig 7) (A) 3D7 parasites were treated for 6 h with 1 μM C2, 50 μM SAK1, or 50–100 μM WSAK starting at 42 h.p.i. (T0). Schizonts were fixed before and after treatment and stained with rabbit anti-AMA1 (red) and human anti-MSP1 (green). DNA was stained with DAPI (blue). IFAs were analysed by confocal microscopy. Scale bar: 10 μm. (B) The amounts of segmented (black) or non-segmented (grey) schizonts was quantified based on the MSP1 staining pattern. Representative images for each category are shown. Results are the means ± standard deviation from three independent biological replicates (Note that treatment at 100 μM WSAK was only performed once). Differences between T0 and the differently treated parasite populations were compared using a Student’s t test, with all significant results indicated. (C) Quantification of the number of nuclei per iRBC. The number of nuclei per iRBC was quantified from the IFA and is shown as a dot plot. Average number of nuclei/iRBC is indicated below the graph. Results are the means ± standard deviation from one representative experiment. Differences between T0 and the differently treated parasite populations were compared using a Student’s t test, with all significant results indicated. (TIF) [file ppat.1007031.s012.tif]

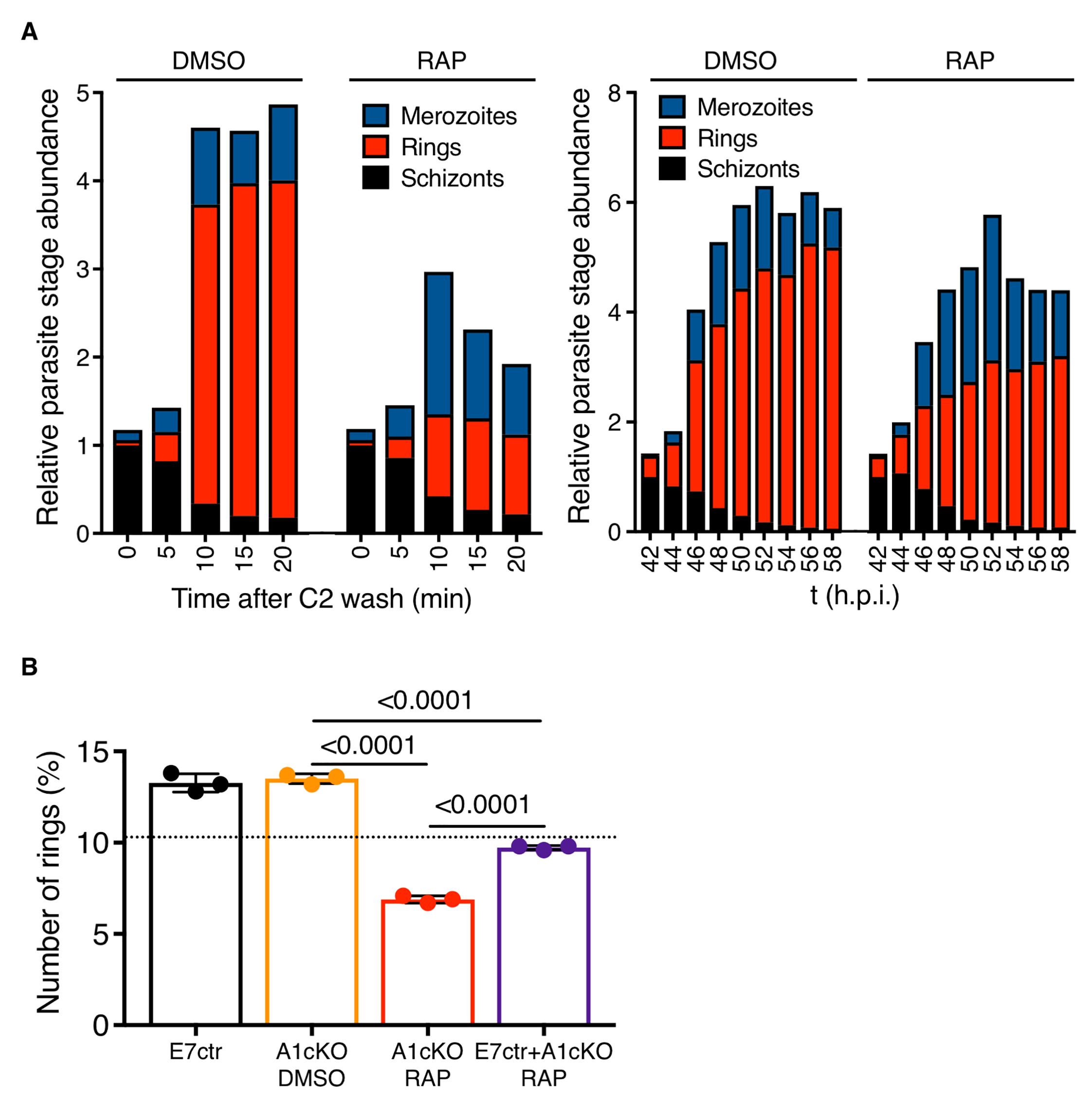

Supplement: S9 Fig — (Related to Fig 8C) (A) Left: C2-arrested A1cKO schizonts pretreated with DMSO or RAP were incubated with fresh RBCs after C2 removal. Samples were collected at the indicated time points, fixed, and stained with Hoechst and WGA-Alexa647. The populations of schizonts, rings, and free merozoites were quantified by FACS and are shown in the bar graph. Right: Same type of staining and FACS analysis was performed on the samples collected at the indicated time points (h.p.i.) after DMSO or RAP treatment. The fixed samples used for this experiment were the same as the ones shown in Fig 6E. (B) Effect of mixing WT and DPAP3KO parasites on RBC invasion. To determine whether secretion of DPAP3 in the culture supernatant could rescue the invasion defect of DPAP3KO parasites, we mixed equal amounts of E7ctrl and DPAP3KO schizonts (obtained after RAP treatment of A1cKO) with fresh RBCs and compared the invasion efficiency of this mixed culture with that of E7ctrl or A1cKO after DMSO or RAP treatment. All cultures were setup at 2% parasitemia and incubated overnight under shaking conditions. The percentage of rings obtained for each culture is shown. Each circle represents a different biological replicate. The dotted line marks the expected invasion rate for the 1:1 mixture of E7ctr and A1cKO after RAP treatment assuming independent effects between the two lines, i.e. no rescue effect. This value (10.1%) was calculated as the average between the E7ctr and A1cKO+RAP invasion rates. Differences between the different parasite populations were compared using a Student’s t test, with all significant results indicated. (TIF) [file ppat.1007031.s013.tif]

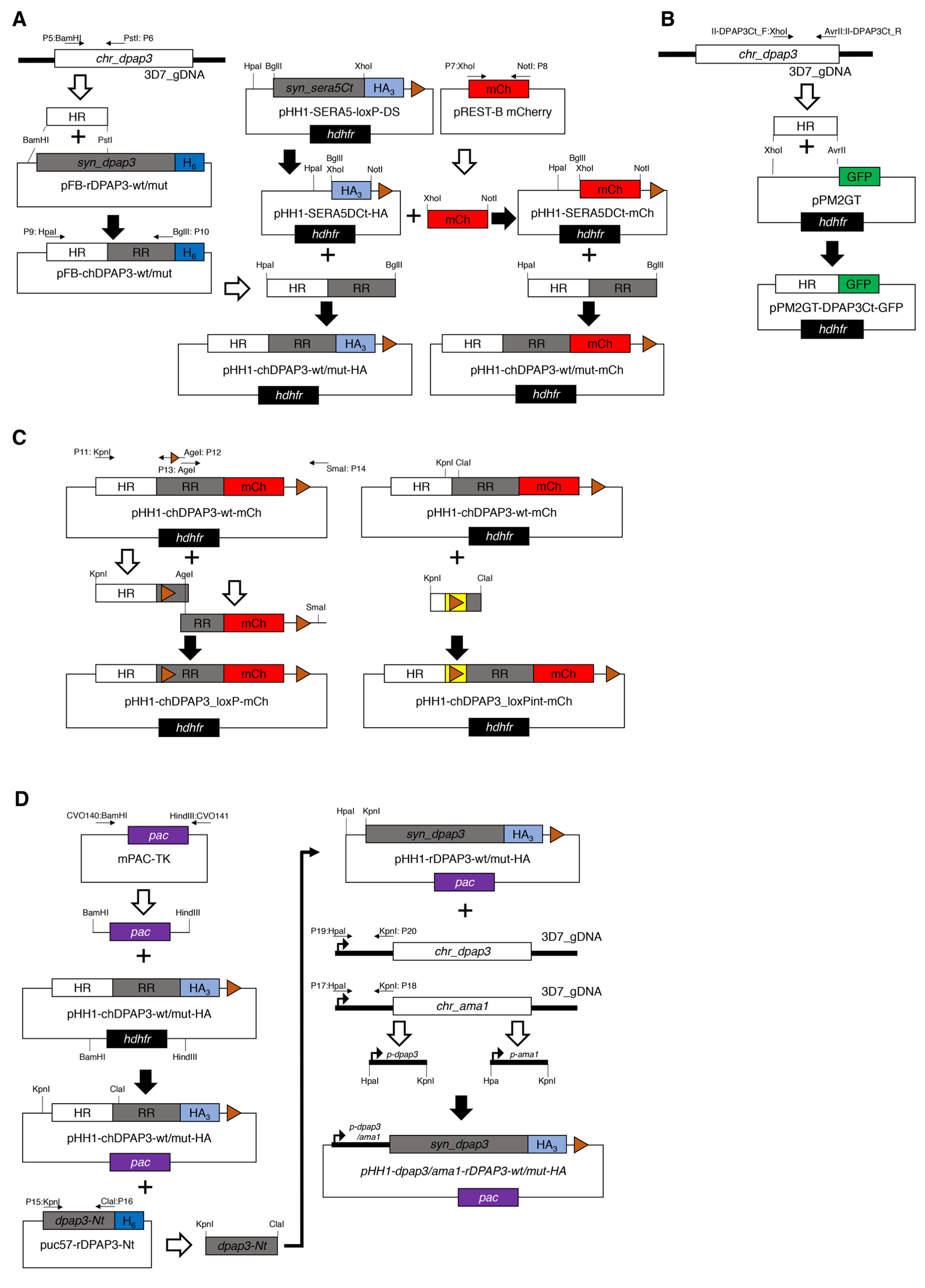

Supplement: S10 Fig — (A) Constructs pHH1-chDPAP3-mCh, pHH1-chDPAP3-HA were designed to integrate by single-crossover homologous recombination into the 3D7 dpap3 locus, reconstituting the coding sequence of the endogenous gene with a gene that expresses a chimeric (ch) DPAP3 protein fused to either mCherry or a triple HA tag. To reliably introduce a mutation at the catalytic cysteine residue, the DNA sequence in the entire C-terminal region (1268 bp) was recodonized in the resultant plasmid (syn_dpap3). Recodonized regions (RR) are shown as grey boxes. Upstream of the recodonized sequence was a 1210 bp endogenous 3D7 sequence (homology region, HR, that excludes the 58 bp sequence coding for the signal peptide) to drive single cross-over homologous recombination at the dpap3 locus. White block arrows in the scheme indicate PCR amplification and black block arrows ligation after digest with the indicated restriction enzymes. The 1210 bp targeting fragment (HR, white box) was created by amplifying P. falciparum 3D7 genomic DNA using primers P5 (forward, has BamHI site) and P6 (reverse, has PstI site) and ligated into pFB-rDPAP3 or pFB-rDPAP3mut after restriction digest with BamHI and PstI resulting in plasmids pFB-chDPAP3 and pFB-chDPAP3mut, respectively. Plasmid pHH1-SERA5-loxP-DS_PbDT3’, which harbors the coding sequence for a C-terminal HA3 tag (light blue box), a loxP site (orange arrow) downstream of the Pb3’ UTR, and a hdhfr resistance cassette (black box)[33], was digested with BglII and XhoI (deleting the C-terminal part of SERA5 in this plasmid), blunted, and re-ligated in order to create a BglII restriction site at the 3’ end (resulting in plasmid pHH1-SERA5ΔCt-HA). The sequence for mCherry (red box) was amplified from plasmid pREST-B mCherry[55] with primer P7 (forward, has SalI and XhoI sites) and P8 (reverse, has SpeI and NotI sites) and ligated into plasmid pHH1-SERA5ΔCt-HA after digestion with XhoI and NotI, resulting in exchange of HA3 with mCherry and plasmid pHH1-SERA5ΔCt- [file ppat.1007031.s014.tif]

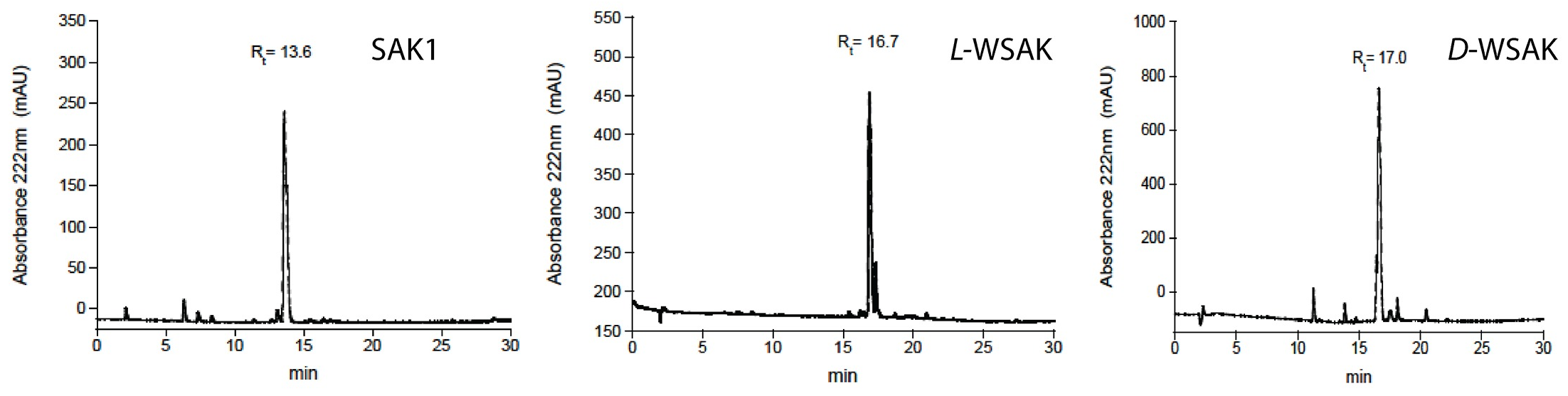

Supplement: S11 Fig — (Related to Fig 7A) LCMS UV trace showing the purity of SAK1, L-WSAK, and D-WSAK. (TIF) [file ppat.1007031.s015.tif]
